# Supplementary material for: Synthesis and preclinical testing of a selective beta-subtype agonist of thyroid hormone receptor ZTA-261
Source: Commun Med (Lond). 2024 Aug 6;4:152. doi: 10.1038/s43856-024-00574-z (PMC11303563; doi:10.1038/s43856-024-00574-z)
Supplement: Supplementary file 2 — Supplementary Information [file 43856_2024_574_MOESM2_ESM.pdf]

# - *Supplementary Information* -

Synthesis and preclinical testing of a selective β-subtype agonist of thyroid hormone receptor ZTA-261

Masakazu Nambo[1,2†*], Taeko Nishiwaki-Ohkawa[1,3†*], Akihiro Ito[3†], Zachary T. Ariki[1†], Yuka Ito[3], Yuuki Kato[3], Muhammad Yar[1‡], Jacky C.-H. Yim[1], Emily Kim[3], Elizabeth Sharkey[3], Keiko Kano[1], Emi Mishiro-Sato[1], Kosuke Okimura[3], Michiyo Maruyama[3], Wataru Ota[3], Yuko Furukawa[1], Tomoya Nakayama[3] Misato Kobayashi[4§], Fumihiko Horio[4¶], Ayato Sato[1,6*], Cathleen M. Crudden[1,5*], Takashi Yoshimura[1,3,6*]

1. Institute of Transformative Bio-Molecules (WPI-ITbM), Nagoya University, Furo-cho, Chikusa-ku, Nagoya 464-8601, Japan
2. Department of Chemistry, Graduate School of Science, Nagoya University, Furo-cho, Chikusa-ku, Nagoya, 464-8601, Japan
3. Laboratory of Animal Integrative Physiology, Department of Animal Sciences, Graduate School of Bioagricultural Sciences, Nagoya University, Furo-cho, Chikusa-ku, Nagoya 464-8601, Japan
4. Laboratory of Animal Nutrition, Department of Animal Sciences, Graduate School of Bioagricultural Sciences, Nagoya University, Furo-cho, Chikusa-ku, Nagoya 464-8601, Japan
5. Department of Chemistry, Queen's University, Chernoff Hall, Kingston, Ontario, K7L 3N6, Canada
6. Center for One Medicine Innovative Translational Research (COMIT), Nagoya University, Nagoya 464-8601, Japan.

[†] These authors contributed equally to this work.
[‡]Present address: Interdisciplinary Research Center in Biomedical Materials, COMSATS, University Islamabad Lahore Campus, Lahore 54000, Pakistan
[§] Present address: Department of Nutritional Sciences, Nagoya University of Arts and Sciences, Nisshin, Aichi 470-0196, Japan
[¶]Present address: Department of Life Studies and Environmental Science, Nagoya Women's University, Nagoya 467-8610, Japan
*Corresponding author
Masakazu Nambo: mnambo@itbm.nagoya-u.ac.jp
Taeko Nishiwaki-Ohkawa: tohkawa@agr.nagoya-u.ac.jp
Ayato Sato: ayato-sato@itbm.nagoya-u.ac.jp
Cathleen M. Crudden: cruddenc@chem.queensu.ca
Takashi Yoshimura: takashiy@agr.nagoya-u.ac.jp

Table of Contents

Supplementary Methods	S2-S14
Supplementary Figure 1	S15
Supplementary Figure 2	S16
Supplementary Figure 3	S17
Supplementary Figure 4	S18
Supplementary Figure 4	S19
Supplementary Tables	S20
^{1}H, ^{13}C, and ^{19}F NMR Spectra of Products	S21-S58
Supplementary References	S59

Supplementary Methods

General for synthesis of compounds

Unless otherwise noted, all materials including dry solvents were obtained from commercial suppliers and used without further purification. 2,6-dimethyl-4-triisopropylsilyloxybenzaldehyde[1], methyl 4-bromo-2,6-bis(trifluoromethyl)benzoate[2], and 4-methoxy-1-naphthaleneboronic acid[3] were prepared according to procedures reported in the literature. Unless otherwise noted, all reactions were performed with dry solvents under an atmosphere of argon in flame-dried glassware with standard vacuum-line techniques. All work-up and purification procedures were carried out with reagent-grade solvents in the air.

Analytical thin-layer chromatography (TLC) was performed using E. Merck silica gel 60 F_{254} precoated plates (0.25 mm) visualizing with UV light (254 nm) and ethanolic phosphomolybdic acid. Preparative thin-layer chromatography (PTLC) was performed using Wakogel B5-F silica coated plates (0.75 mm) prepared in our laboratory. Reverse phase HPLC was performed with Biotage Isolera instrument equipped with a Biotage SNAP Ultra C18 Cartridge (12g) using MeCN/H_2O as eluent. Gas chromatographic (GC) analysis was conducted on a Shimadzu GC-2010 instrument equipped with an HP-5 column (30 m × 0.25 mm, Hewlett-Packard). GCMS analysis was conducted on a Shimadzu GCMS-QP2010 instrument equipped with an HP-5 column (30 m × 0.25 mm, Hewlett-Packard). Column chromatography was performed using flash grade silica (silica gel 60, spherical, particle size 40-50 μm).

High-resolution mass spectra (HRMS) were obtained from a Thermo Fisher Scientific Exactive (ESI) and a JMS-T100TD instrument (DART). Nuclear magnetic resonance (NMR) spectra were recorded on a JEOL ECA600II (^{1}H 600 MHz, ^{13}C 150 MHz) and a JEOL ECS-400 (^{1}H 400 MHz, ^{13}C 100 MHz, ^{19}F 376 MHz) spectrometers. Chemical shifts for ^{1}H NMR are expressed in parts per million (ppm) relative to tetramethylsilane (δ 0.00 ppm) or residual proton signal in CD_3OD (δ 3.31 ppm). Chemical shifts for ^{13}C NMR are expressed in ppm relative to $CDCl_3$ (δ 77.0 ppm) or residual proton signal in CD_3OD (δ 49.0 ppm). Data are reported as follows: chemical shift, multiplicity (s = singlet, d = doublet, dd = double doublet, t = triplet, dt = double triplet, q = quartet, hep =heptet, m = multiplet, br = broad signal), coupling constant (Hz), and integration.

Synthesis of MY-53

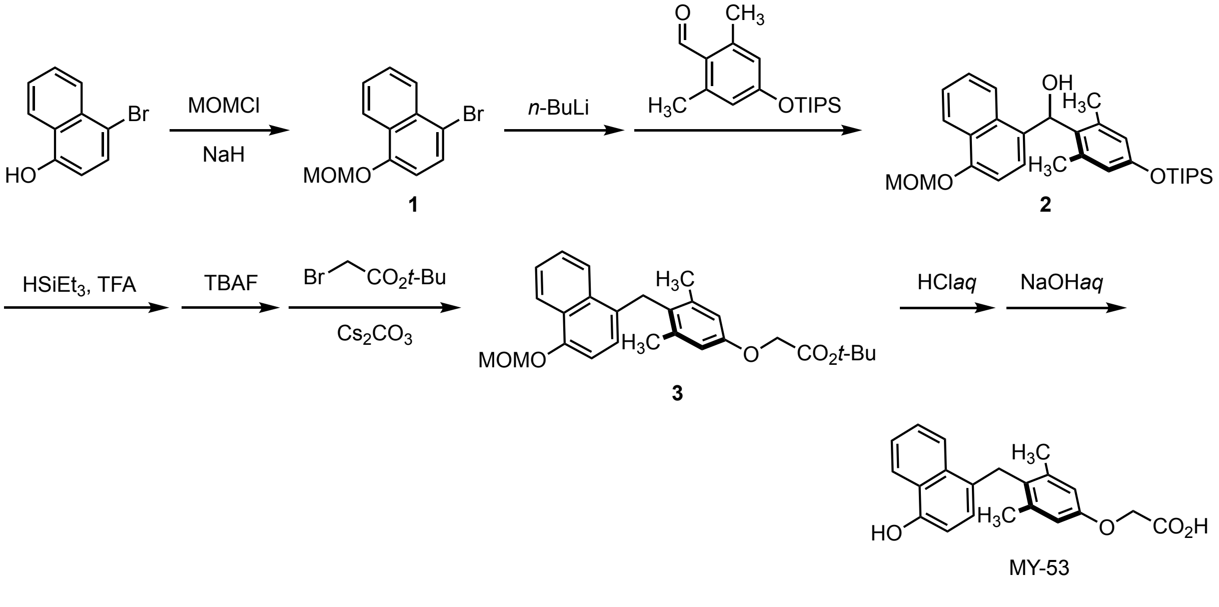

Preparation of 1

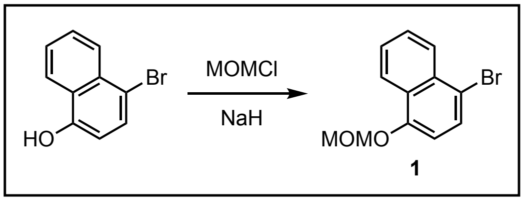

A 100-mL flask containing a magnetic stirring bar was flame-dried under vacuum and filled with argon after cooling to room temperature. To the flask were added 4-bromonaphthol (5.57 g, 24.9 mmol) and dry DMF (20 mL) under a stream of argon. NaH (60% dispersion in mineral oil, 719 mg, 30.0 mmol) was added to this mixture at 0 ºC. After stirring at 0 ºC for 10 min, chloromethyl methyl ether (1.9 mL, 24.9 mmol) was slowly added at this temperature and the mixture was stirred at room temperature for 16 h. The mixture was quenched with cold water and extracted with ether (3 times). The combined extracts were dried over Na_2SO_4 and the solvent was evaporated under reduced pressure. The crude material was purified by column chromatography (Hex/EtOAc = 20:1) to give 1-bromo-4-(methoxymethoxy)naphthalene **1** (5.00 g, 75%) as a colorless oil.

^{1}H NMR (400 MHz, $CDCl_3$) δ 3.54 (s, 3H), 5.38 (s, 2H), 6.98 (d, *J* = 8.4 Hz, 1H), 7.52-7.56 (m, 1H), 7.59-7.63 (m, 1H), 7.66 (d, *J* = 8.0 Hz, 1H), 8.18 (d, *J* = 9.2 Hz, 1H), 8.29 (d, *J* = 8.0 Hz, 1H). ^{13}C NMR (400 MHz, $CDCl_3$) δ 56.3, 94.7, 108.4, 114.5, 122.3, 126.1, 126.9, 127.1, 127.6, 129.5, 132.5, 152.6. HRMS (ESI) *m/z* calcd for $C_{12}H_{11}O_2Br$ [M]+: 265.9937, found 265.9938.

Preparation of 2

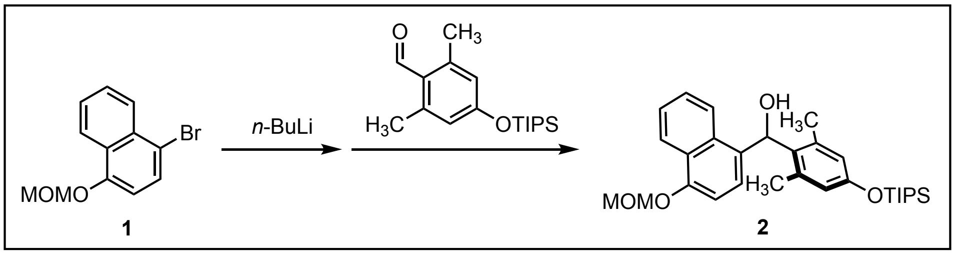

A 100-mL flask containing a magnetic stirring bar was flame-dried under vacuum and filled with argon after cooling to room temperature. To the flask was added 1-bromo-4-(methoxymethoxy)naphthalene (870 mg, 3.26 mmol) and dry THF (10 mL) under a stream of argon. *n*-BuLi (1.6 M in hexane, 2.3 mL, 3.6 mmol) was slowly added to this solution at -78 ºC. After stirring at -78 ºC for 1 h, a solution of 2,6-dimethyl-4-triisopropylsilyloxybenzaldehyde (1.00 g, 3.26 mmol) in THF (15 mL) was added at this temperature and the mixture was stirred at room temperature for 16 h. The mixture was quenched with sat. NH_4Cl*aq* and extracted with ether (3 times). The combined extracts were dried over Na_2SO_4 and the solvent was evaporated under reduced pressure. The crude material was purified by column chromatography (Hex/EtOAc = 5:1) to give (2,6-dimethyl-4-triisopropylsilyloxyphenyl)[4'-(methoxymethoxy)naphthyl]methanol **2** (835 mg, 52%) as colorless oil.

^{1}H NMR (600 MHz, $CDCl_3$) δ 1.12 (d, *J* = 7.8 Hz, 18H), 1.26 (hep, *J* = 7.8 Hz, 3H), 2.24 (s, 6H), 3.52 (s, 3H), 5.35 (s, 2H), 6.60 (s, 2H), 6.74 (s, 1H), 6.92 (d, *J* = 7.8 Hz, 1H), 7.11 (d, *J* = 7.8 Hz, 1H), 7.48-7.52 (m, 2H), 8.20 (d, *J* = 7.8 Hz, 1H), 8.33 (d, *J* = 7.8 Hz, 1H). ^{13}C NMR (150 MHz, $CDCl_3$) δ 12.7, 18.0, 21.4, 56.2, 70.5, 94.6, 106.6, 120.6, 122.5, 124.3, 125.1, 125.3, 126.4, 126.6, 130.1, 131.1, 132.6, 138.4, 152.8, 154.9. HRMS (ESI) *m/z* calcd for $C_{30}H_{42}O_4SiNa$ [M+Na]+: 517.2745, found 517.2736.

Preparation of 3

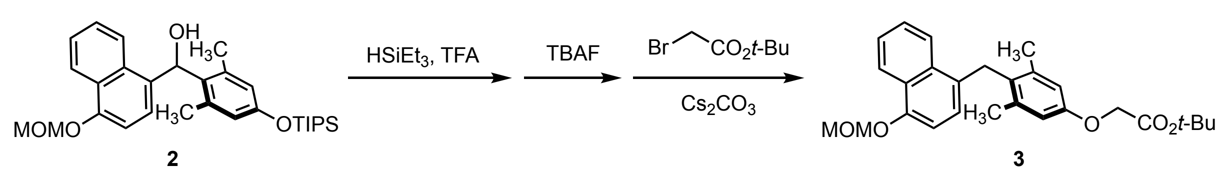

A 50-mL flask containing a magnetic stirring bar was flame-dried under vacuum and filled with argon after cooling to room temperature. To the flask was added (2,6-dimethyl-4-triisopropylsilyloxyphenyl)[4'-(methoxymethoxy)naphthyl]methanol (226 mg, 0.5 mmol) and dry DCM (5.5 mL) under a stream of argon. TFA (150 µL, 2 mmol) was added to this solution at 0 ºC, and then $HSiEt_3$ (2.3 mL, 1.25 mmol) was added. After stirring at 0 ºC for 10 min, the mixture was quenched with sat. Na_2CO_3*aq* and extracted with EtOAc (3 times). The combined extracts were dried over Na_2SO_4 and the solvent was evaporated under reduced pressure. The residue was dissolved in TBAF (1M in THF, 3 mL, 3 mmol), and the mixture was stirred at room temperature for 20 min. The mixture was quenched with water and extracted with EtOAc (3 times). The combined extracts were dried over Na_2SO_4 and the solvent was evaporated under reduced pressure. The residue was dissolved in dry DMF (2 mL), and Cs_2CO_3 (489 mg, 1.5 mmol) was added. Then, *tert*-butyl bromoacetate (60 µL, 0.4 mmol) was added and the mixture was stirred at room temperature for 12 h. The mixture was quenched with sat. NH_4Cl*aq* and extracted with ether (3 times). The combined extracts were dried over Na_2SO_4 and the solvent was evaporated under reduced pressure. The crude material was purified by PTLC (Hex/EtOAc = 10:1) and GPC to give *tert*-butyl 3,5-dimethyl-4-[4'-(methoxymethoxy)naphthylmethyl]phenoxyacetate **3** (86.4 mg, 40%) as a colorless oil.

^{1}H NMR (600 MHz, $CDCl_3$) δ 1.51 (s, 9H), 2.15 (s, 6H), 3.52 (s, 3H), 4.27 (s, 2H), 4.53 (s, 2H), 5.32 (s, 2H), 6.52 (d, *J* = 7.2 Hz, 1H), 6.67 (s, 2H), 6.85 (d, *J* = 8.4 Hz, 1H), 7.54 (t, *J* = 7.2 Hz, 1H), 7.61 (t, *J* = 7.2 Hz, 1H), 8.18 (d, *J* = 8.4 Hz, 1H), 8.35 (d, *J* = 7.2 Hz, 1H). ^{13}C NMR (150 MHz, $CDCl_3$) δ 20.2, 28.0, 30.7, 56.2, 65.8, 82.2, 94.7, 107.6, 114.2, 122.6, 123.0, 123.4, 125.0, 126.1, 126.3, 128.3, 129.4, 133.0, 138.9, 151.5, 156.1, 168.3. HRMS (ESI) *m/z* calcd for $C_{27}H_{32}O_5Na$ [M+Na]+: 459.2142, found 459.2143.

Preparation of MY-53

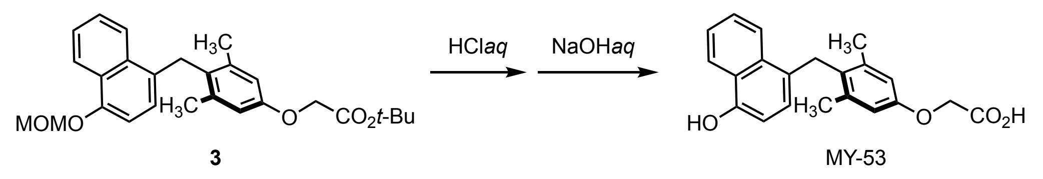

To a 50 mL flask was added *tert*-butyl 3,5-dimethyl-4-[4'-(methoxymethoxy)naphthylmethyl]-phenoxyacetate **3** (55 mg, 0.13 mmol) followed by *i*-PrOH (1.5 mL) and THF (1.5 mL). Then, 2N HCl*aq* (2.0 mL) was added and the reaction was stirred at 60 ºC for 5 h. Brine was added and the organic layer was extracted with EtOAc (3 x 10 mL), washed with brine, dried over sodium sulfate, and concentrated *in vacuo*. The crude material was dissolved in MeOH (5 mL) and 2N NaOH*aq* (2.0 mL). After stirring at room temperature for 2 h, the solvent was evaporated under reduced pressure. 1N HCl*aq* (5.0 mL) was added, and extracted with EtOAc (3 times). The combined extracts were dried over Na_2SO_4 and the solvent was evaporated under reduced pressure. The crude material was purified via reverse-phase HPLC (Biotage SNAP Cartridge 12g) to give MY-53 (23

mg, 52%) as a white solid. The purity of MY-53 was determined to be >96% by HPLC analysis. ^{1}H NMR (400 MHz, CD_3OD) δ 2.14 (s, 6H), 4.24 (s, 2H), 4.64 (s, 2H), 6.36 (d, J = 8.0 Hz, 2H), 6.56 (d, J = 8.0 Hz, 2H), 6.70 (s, 2H), 7.42-7.48 (m, 1H), 7.53-7.57 (m, 1H), 8.15 (d, J = 8.4 Hz, 1H), 8.25 (d, J = 8.4 Hz, 1H). ^{13}C NMR (100 MHz, CD_3OD) δ 20.2, 31.4, 65.9, 108.3, 115.1, 123.89, 123.92, 124.5, 125.3, 126.7, 127.0, 127.2, 131.1, 134.5, 139.9, 153.0, 157.6, 173.1. HRMS (ESI) m/z calcd for $C_{21}H_{19}O_4$ [M-H]-: 335.1278, found 335.1284.

HPLC chart of MY-53

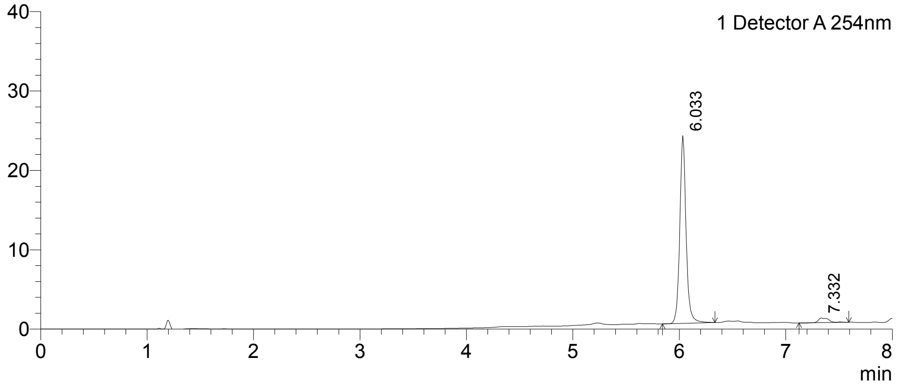

Peak Table

Detector A 254nm

Peak#	Ret. Time	Area	Height	Conc.	Unit	Mark	Name
1	6.033	97008	23648	96.156		M	
2	7.332	3878	587	3.844		M	
Total		100886	24235				

Synthesis of ZTA-245

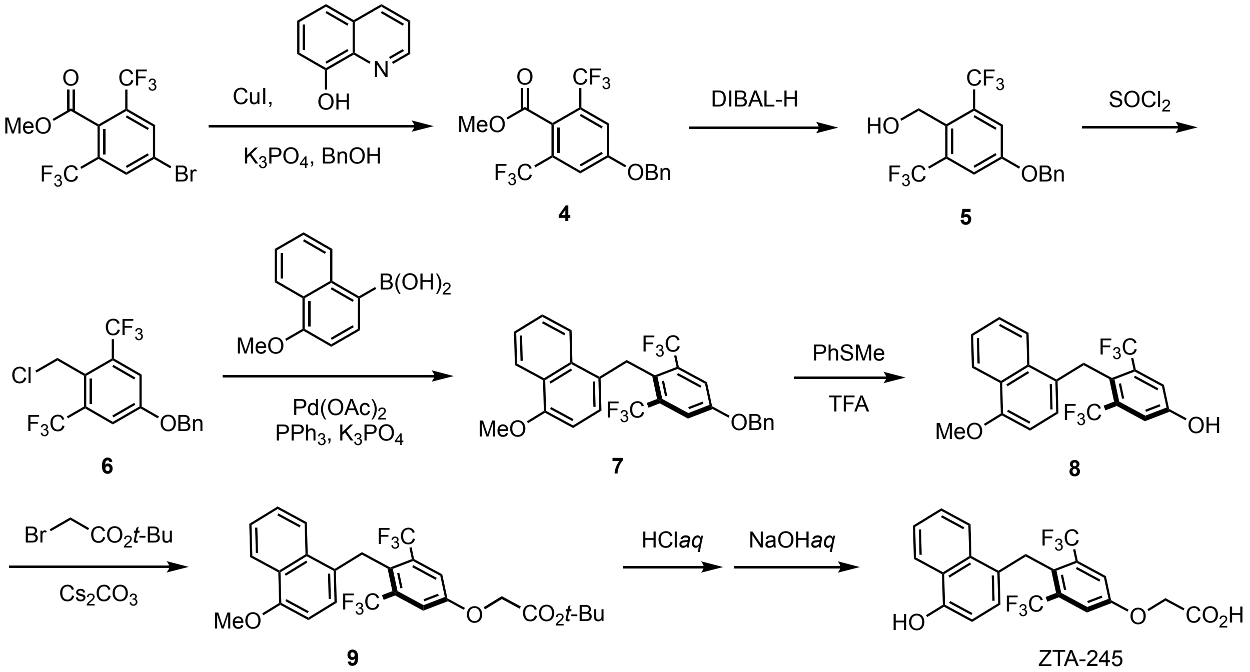

Preparation of 4

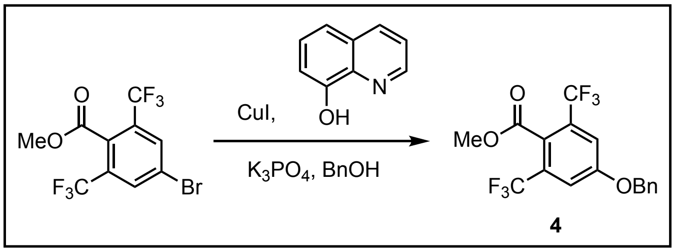

A 10 mL flask equipped with a Teflon cap containing a magnetic stirring bar was flame-dried under vacuum and filled with argon after cooling to room temperature. To the flask was added methyl 4-bromo-2,6-bis(trifluoromethyl)benzoate (1.05 g, 3.0 mmol), CuI (17.1 mg, 0.09 mmol), 8-hydroxyquinoline (26.1 mg, 0.18 mmol), K_3PO_4 (1.27 g, 6.0 mmol). The flask was evacuated and backfilled with argon. Anhydrous benzyl alcohol (3.0 mL) was then added at room temperature and the mixture was stirred at 110 °C for 24 h. The reaction mixture was then cooled to room temperature diluted with Et_2O (~15 mL). The mixture was then quenched with sat. $NH_4Cl_{(aq)}$ (~ 20 mL) and separated. The aqueous layer was extracted with Et_2O (2 x 15 mL) and the combined organic layer was washed with H_2O (2 x 25 mL), brine (25 mL), dried over sodium sulfate, filtered and concentrated *in vacuo*. The crude material was purified by column chromatography (30:1 → 25:1 hexanes:ethyl acetate) to give methyl 4-(benzyloxy)-2,6-bis(trifluoromethyl)benzoate **4** as a while solid (0.852 g, 75%).

^{1}H NMR (400 MHz, $CDCl_3$) δ: 3.93 (s, 3H), 5.16 (s, 2H), 7.36-7.44 (m, 7H). ^{13}C NMR (100 MHz, $CDCl_3$) δ: 53.3, 70.9, 116.0 (m), 122.6 (q, J = 272.9 Hz), 122.7, 127.6, 128.7, 128.9, 130.7 (q, J = 31.7 Hz), 134.8, 159.2, 165.6. ^{19}F NMR (376 MHz, $CDCl_3$) δ: -60.0 ppm. HRMS (ESI): *m/z* calcd for $C_{17}H_{12}F_6O_3Na$ ($[M+Na]^+$) 401.0588, found 401.0583.

Preparation of 5

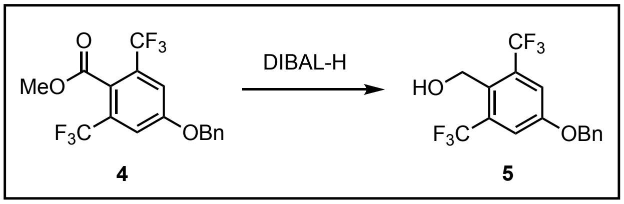

A 50 mL flask was flame-dried under vacuum and filled with argon after cooling to room temperature. To the flask was added methyl 4-(benzyloxy)-2,6-bis(trifluoromethyl)benzoate **4** (1.45 g, 3.82 mmol) followed by dry DCM (13 mL). The mixture was cooled to 0°C and then diisobutylaluminumhydride (11.5 mL, 11.5 mmol, 1M solution in hexanes) was added dropwise at that temperature. The reaction mixture was then stirred at room temperature for 3 h and then reaction was quenched water (20 mL) and the mixture became a gel. The gel was washed with 2N HCl (3 x 50 mL) and the organic layer was extracted with EtOAc (3 x 50 mL), washed with brine, dried over sodium sulfate, and concentrated *in vacuo*. The crude material was purified by column chromatography (Hex/EtOAc = 30:1) to give (4-(benzyloxy)-2,6-bis(trifluoromethyl)phenyl)methanol **5** (1.45 g, 83%) as a white solid.

^{1}H NMR (400 MHz, $CDCl_3$) δ 2.03 (s, 1H), 4.83 (s, 2H), 5.11 (s, 2H), 7.33-7.43 (m, 5H), 7.47 (s, 2H). ^{13}C NMR (100 MHz, $CDCl_3$) δ 56.6, 70.6, 116.3 (q, J = 5.7 Hz), 123.5 (q, J = 279.4 Hz), 127.6, 128.6, 128.8, 129.1, 132.9 (q, J = 31.5 Hz), 135.2, 158.0. ^{19}F NMR (376 MHz, $CDCl_3$) δ: -58.3. HRMS (ESI) *m/z* calcd for $C_{16}H_{12}O_2F_6Na$ [M+Na]+: 373.0634, found 373.0634.

Preparation of 6

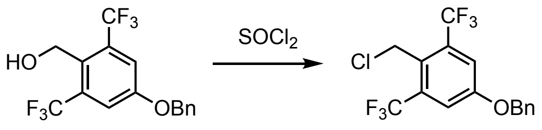

5 **6**

A 50 mL flask was flame-dried under vacuum and filled with argon after cooling to room temperature. To the flask was added (4-(benzyloxy)-2,6-bis(trifluoromethyl)phenyl)methanol **5** (1.29 g, 3.68 mmol) followed by dry DCM (3.70 mL). The mixture was cooled to 0°C and then thionyl chloride (657 mg, 5.52 mmol) was added dropwise at that temperature. The reaction was warmed to room temperature and stirred for 16 h. The reaction mixture was concentrated *in vacuo* and the crude material was purified by column chromatography (Hex/EtOAc = 50:1) to give 5-(benzyloxy)-2-(chloromethyl)-1,3-bis(trifluoromethyl)benzene **6** (1.22 g, 90%) as a clear oil.
^{1}H NMR (400 MHz, $CDCl_3$) δ 4.79 (s, 2H), 5.07 (s, 2H), 7.34-7.42 (m, 5H), 7.46 (s, 2H). ^{13}C NMR (100 MHz, $CDCl_3$) δ 36.7, 70.7, 116.6 (q, *J* = 5.7 Hz), 123.3 (q, *J* = 279.4 Hz), 126.1, 127.6, 128.6, 128.8, 132.9 (q, *J* = 31.5 Hz), 135.1, 158.6. ^{19}F NMR (376 MHz, $CDCl_3$) δ: -58.9. HRMS (DART) *m/z* calcd for $C_{16}H_{10}OF_6Cl$ [M-H]+: 367.0324, found 367.0322.

Preparation of 7

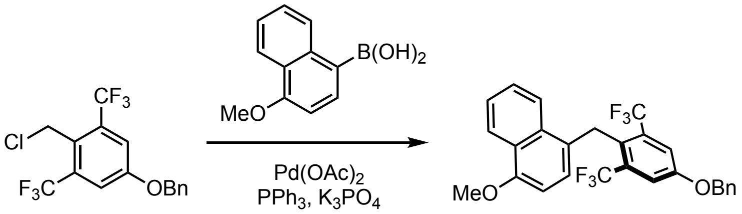

6 **7**

A 50 mL flask was flame-dried under vacuum and filled with argon after cooling to room temperature. To the flask was added 5-(benzyloxy)-2-(chloromethyl)-1,3-bis(trifluoromethyl)benzene **6** (500 mg, 1.36 mmol), (4-methoxynaphthalen-1-yl)boronic acid (411 mg, 2.03 mmol), $Pd(OAc)_2$ (30.4 mg, 0.14 mmol), PPh_3 (71.3 mg, 0.27 mmol), and K_3PO_4 (1.15 g, 5.42 mmol). The flask was evacuated and backfilled with argon. Then dry toluene (4.50 mL) was added at room temperature and the mixture was stirred at 90 °C for 16 h. After cooling to room temperature, water was added (20 mL) and the organic layer was extracted with ether (3 x 50 mL). The organic layer was washed with brine, dried over sodium sulfate, and concentrated *in vacuo*. The crude material was purified by column chromatography (Hex/EtOAc = 40:1) to give 1-(4-(benzyloxy)-2,6-bis(trifluoromethyl)benzyl)-4-methoxynaphthalene **7** (516 mg, 77%) as a white solid.
^{1}H NMR (400 MHz, $CDCl_3$) δ 3.92 (s, 3H), 4.63 (s, 2H), 5.17 (s, 2H), 6.26 (d, *J* = 8.2 Hz, 1H), 6.57 (d, *J* = 8.2 Hz, 1H), 7.37-7.63 (m, 9H), 8.07 (d, *J* = 8.2 Hz, 1H), 8.32 (d, *J* = 8.2 Hz, 1H). ^{13}C NMR (100 MHz, $CDCl_3$) δ 30.2, 55.3, 70.7, 102.9, 116.6 (q, *J* = 5.7 Hz), 122.5, 122.6, 123.5 (q, *J* = 279.4 Hz), 124.3, 124.9, 125.5, 126.6, 127.7, 128.6, 128.8, 128.9, 132.0, 133.5 (q, *J* = 31.4 Hz), 135.5, 153.9, 157.2. ^{19}F NMR (376 MHz, $CDCl_3$) δ: -59.7. HRMS (DART) *m/z* calcd for $C_{27}H_{21}F_6O_2$ [M+H]+: 491.1446, found 491.1445.

Preparation of 8

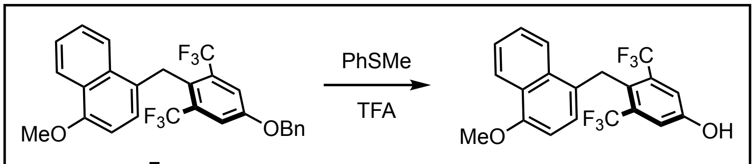

A 50 mL flask was flame-dried under vacuum and filled with argon after cooling to room temperature. To the flask was added 1-(4-(benzyloxy)-2,6-bis(trifluoromethyl)benzyl)-4-methoxynaphthalene **7** (127 mg, 0.26 mmol) followed by trifluoroacetic acid (2.6 mL). Then, thioanisole (1.61 g, 13.0 mmol) was added dropwise at room temperature. The reaction mixture was stirred for 22 h and then the reaction was concentrated *in vacuo*. The crude material was dissolved in EtOAc (20 mL) and washed with water (3 x 50 mL). The organic layer was collected and washed with brine, dried over sodium sulfate, and concentrated *in vacuo*. The crude material was purified by column chromatography (Hex/EtOAc = 10:1) to give 4-((4-methoxynaphthalen-1-yl)methyl)-3,5-bis(trifluoromethyl)phenol **8** as a white solid (95 mg, 92%).

^{1}H NMR (400 MHz, $CDCl_3$) δ 3.92 (s, 3H), 4.61 (s, 2H), 6.26 (d, *J* = 8.4 Hz, 1H), 6.57 (d, *J* = 8.4 Hz, 1H), 7.44 (s, 2H), 7.52 (ddd, *J* = 0.8, 7.6, 7.6 Hz, 1H), 7.61 (ddd, *J* = 0.8, 7.7, 7.7 Hz, 1H), 8.07 (d, *J* = 8.4 Hz, 1H), 8.32 (d, *J* = 8.4 Hz, 1H). ^{13}C NMR (100 MHz, $CDCl_3$) δ 30.1, 55.3, 103.0, 117.3 (q, *J* = 5.7 Hz), 122.5, 122.6, 123.3 (q, *J* = 279.4 Hz), 124.3, 125.0, 125.5, 126.6, 128.6, 128.9, 132.0, 133.6 (q, *J* = 34.2 Hz), 153.9, 154.2. ^{19}F NMR (376 MHz, $CDCl_3$) δ: -59.9. HRMS (ESI) *m/z* calcd for $C_{20}H_{13}F_6O_2$ [M-H]+: 399.0814, found 399.0822.

Preparation of 9

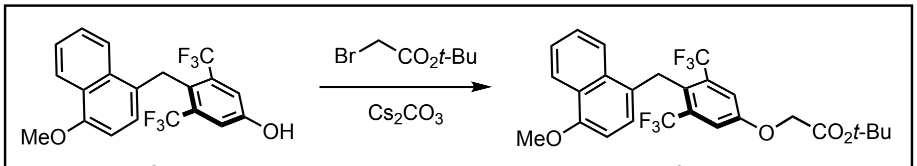

A 50 mL flask was flame-dried under vacuum and filled with argon after cooling to room temperature. To the flask was added 4-((4-methoxynaphthalen-1-yl)methyl)-3,5-bis(trifluoromethyl)phenol **8** (200 mg, 0.50 mmol) and cesium carbonate (705 mg, 2.0 mmol) followed by dry DMF (2.5 mL). Then, tert-butyl bromoacetate (219 mg, 1.12 mmol) was added dropwise at room temperature and the reaction was stirred for 3 h. Water (20 mL) was added and the organic layer was extracted with ether (3 x 20 mL), washed with brine, dried over sodium sulfate, and concentrated *in vacuo*. The crude material was purified by PTLC (Hex/EtOAc = 10:1) to give *tert*-butyl 2-(4-((4-methoxynaphthalen-1-yl)methyl)-3,5-bis(trifluoromethyl)phenoxy)acetate **9** (196 mg, 76%) as a clear oil.

^{1}H NMR (400 MHz, $CDCl_3$) δ 1.51 (s, 9H), 3.90 (s, 2H), 4.63 (s, 3H), 6.26 (d, *J* = 8.4 Hz, 1H), 6.56 (d, *J* = 8.4 Hz, 1H), 7.49-7.53 (m, 3H), 7.60 (ddd, *J* = 1.2, 8.4, 8.4 Hz, 1H), 8.06 (d, *J* = 8.4 Hz, 1H), 8.32 (d, *J* = 8.4 Hz, 1H). ^{13}C NMR (100 MHz, $CDCl_3$) δ 27.9, 30.2, 55.2, 65.8, 83.2, 102.9, 116.5 (q, *J* = 6.7 Hz), 122.5, 122.6, 123.4 (q, *J* = 279.5 Hz), 124.4, 124.9, 125.5, 126.6, 128.4, 129.7, 132.0, 133.5 (q, *J* = 30.5 Hz), 153.9, 156.3, 166.9. ^{19}F NMR (376 MHz, $CDCl_3$) δ: -59.7. HRMS (ESI) *m/z* calcd for $C_{26}H_{24}F_6O_4Na$ [M+Na]+: 537.1471, found 537.1471.

Preparation of ZTA-245

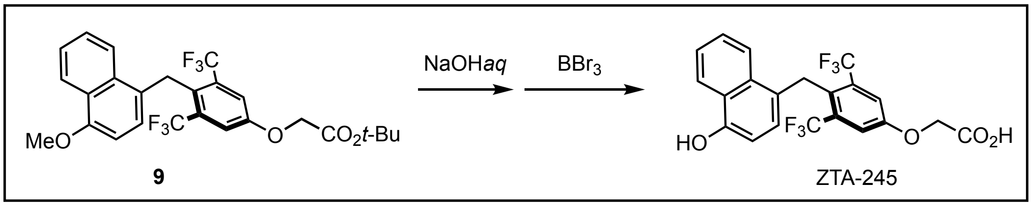

To a 50 mL flask was *tert*-butyl 2-(4-((4-methoxynaphthalen-1-yl)methyl)-3,5-bis(trifluoromethyl)phenoxy)acetate **9** (196 mg, 0.38 mmol) added followed by MeOH (0.6 mL) and THF (0.6 mL). Then, 3N NaOH (0.6 mL) was added and the reaction was stirred at room temperature for 3 h. 1N HCl (10 mL) was added and the organic layer was extracted with EtOAc (3 x 10 mL), washed with brine, dried over sodium sulfate, and concentrated *in vacuo*. The crude material was dissolved in dry DCM (3.8 mL) and cooled to -78°C. Then, BBr_3 (1.14 mL, 1.14 mmol, 1M solution in DCM) was added dropwise and the reaction was stirred at -78°C for 1.5 h. The reaction was then warmed to room temperature and stirred for 45 min. Finally, the reaction was poured onto ice and the organic layer was extracted with EtOAc (3 x 15 mL), washed with brine, dried over sodium sulfate, and concentrated *in vacuo*. The crude material was purified via reverse-phase HPLC (Biotage SNAP Cartridge 12g) to give ZTA-245 (114 mg, 67%) as a white solid. The purity of ZTA-245 was determined to be >97% by HPLC analysis.
^{1}H NMR (400 MHz, $CDCl_3$) δ 4.63 (s, 2H), 4.81 (s, 2H), 6.15 (d, J = 8.8 Hz, 1H), 6.56 (d, J = 7.6 Hz, 1H), 7.51-7.56 (m, 3H), 7.60-7.64 (m, 1H), 8.07 (d, J = 8.4 Hz, 1H), 8.24 (d, J = 8.8 Hz, 1H). ^{13}C NMR (100 MHz, $CDCl_3$) δ 30.2, 64.9, 107.8, 116.5 (q, J = 5.7 Hz), 122.3, 122.7, 123.2 (q, J = 279.4 Hz), 124.4, 125.1, 126.7, 128.8, 130.3, 132.1, 133.7 (q, J = 30.5 Hz), 149.9, 155.9, 172.5. ^{19}F NMR (376 MHz, $CDCl_3$) δ: -59.7. HRMS (ESI) *m/z* calcd for $C_{21}H_{13}F_6O_4$ [M-H]+: 443.0713, found 443.0720.

HPLC chart of ZTA-245

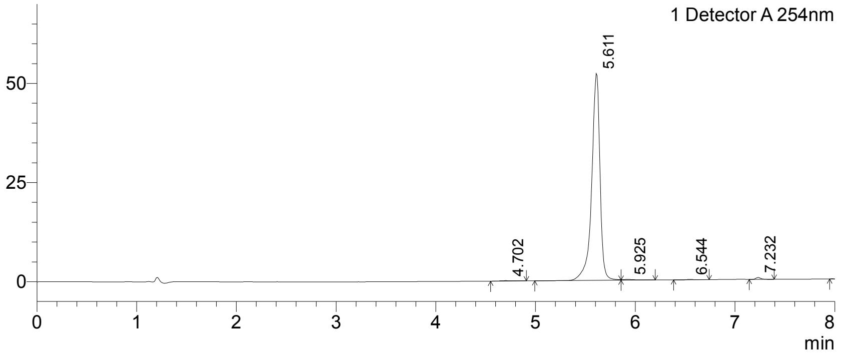

Peak Table

Detector A 254nm

Peak#	Ret. Time	Area	Height	Conc.	Unit	Mark	Name
1	4.702	637	88	0.206		M	
2	5.611	302334	52201	97.962		M	
3	5.925	1604	188	0.520		V M	
4	6.544	398	111	0.129		M	
5	7.232	1690	482	0.548		M	
6	8.096	580	168	0.188		M	
7	8.633	1382	334	0.448		M	
Total		308624	53572				

Synthesis of ZTA-261

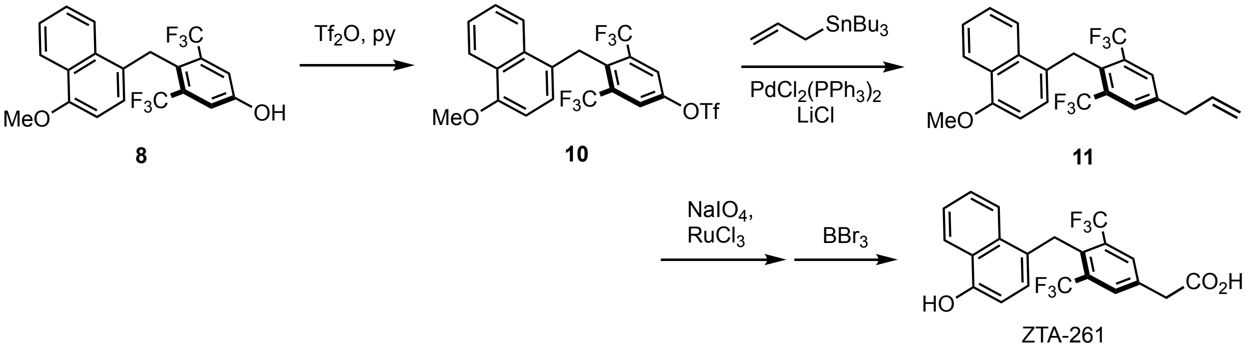

Preparation of 10

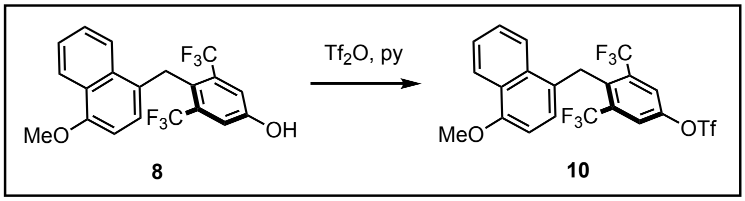

A 10 mL flask was flame-dried under vacuum and filled with argon after cooling to room temperature. To the flask was added 4-((4-methoxynaphthalen-1-yl)methyl)-3,5-bis(trifluoromethyl)phenol **8** (123 mg, 0.31 mmol) followed by dry DCM (0.62 mL). Then, pyridine (48.8 mg, 0.62 mmol) was added at room temperature and the mixture was cooled to 0 °C followed by the dropwise addition of trifluoromethanesuflonic anhydride (104 mg, 0.37 mmol). The reaction mixture was warmed to room temperature and stirred for 1 h. The reaction was quenched with 1N HCl (3 mL) and then saturated $NaHCO_3$ was added (15 mL). The organic layer was extracted with EtOAc (3 x 7 mL), washed with brine, dried over sodium sulfate, and concentrated *in vacuo*. The crude material was purified by PTLC (Hex/EtOAc = 20:1) to give 4-((4-methoxynaphthalen-1-yl)methyl)-3,5-bis(trifluoromethyl)phenyl trifluoromethanesulfonate **10** (130 mg, 79%) as a white solid.

^{1}H NMR (400 MHz, $CDCl_3$) δ 3.91 (s, 3H), 4.73 (s, 2H), 6.15 (d, *J* = 8.4 Hz, 1H), 6.57 (d, *J* = 8.4 Hz, 1H), 7.54 (t, *J* = 8.0 Hz, 1H), 7.62 (t, *J* = 7.8 Hz, 1H), 7.90 (s, 2H), 8.03 (d, *J* = 8.4 Hz, 1H), 8.34 (d, *J* = 8.4 Hz, 1H). ^{13}C NMR (100 MHz, $CDCl_3$) δ 30.7, 55.3, 102.9, 118.7 (q, *J* = 325.2 Hz), 122.3, 122.5 (q, *J* = 279.5 Hz), 122.8, 123.6 (q, *J* = 6.7 Hz), 124.4, 125.2, 125.6, 126.9, 127.2, 131.9, 134.8 (q, *J* = 32.4 Hz), 128.8, 147.4, 154.3. ^{19}F NMR (376 MHz, $CDCl_3$) δ: -60.0, -72.4. HRMS (DART) *m/z* calcd for $C_{21}H_{14}F_6O_4S$ [M+H]+: 533.0469, found 533.0465.

Preparation of 11

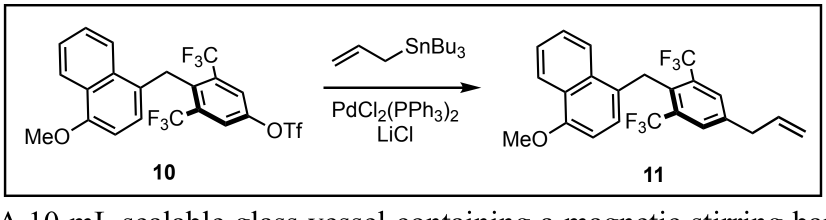

A 10 mL sealable glass vessel containing a magnetic stirring bar was flame-dried under vacuum and filled with argon after cooling to room temperature. To the glass vessel was added 4-((4-methoxynaphthalen-1-yl)methyl)-3,5-bis(trifluoromethyl)phenyl trifluoromethanesulfonate **10** (221 mg, 0.42 mmol) and LiCl (53.0 mg, 1.25 mmol). The vessel was evacuated and backfilled with argon. Then dry DMF (1.2 mL) was added at room temperature followed by

allyltributylstannane (137 mg, 0.42 mmol) and then $PdCl_2(PPh_3)_2$ (5.8 mg, 0.008 mmol). The vessel was capped and sealed under a stream of argon. The mixture was stirred at 80 °C for 16 h. Water (15 mL) was added and the organic layer was extracted with ether (3 x 15 mL), washed with brine, dried over sodium sulfate, and concentrated *in vacuo*. The crude material was purified by PTLC (Hex/EtOAc = 20:1) to give 1-(4-allyl-2,6-bis(trifluoromethyl)benzyl)-4-methoxynaphthalene **11** as a clear oil (120 mg, 68%).

^{1}H NMR (400 MHz, $CDCl_3$) δ 3.53 (d, J = 6.8 Hz, 1H), 3.91 (s, 3H), 5.16-5.24 (tm, 2H), 5.95-6.05 (m, 1H), 6.22 (d, J = 8.4 Hz, 1H), 6.56 (d, J = 8.0 Hz, 1H), 7.50-7.54 (m, 1H), 7.59-7.63 (m, 1H), 7.80 (s, 2H), 8.08 (d, J = 7.6 Hz, 1H), 8.32 (d, J = 8.4 Hz, 1H). ^{13}C NMR (100 MHz, $CDCl_3$) δ 30.6, 39.4, 55.3, 102.9, 117.7, 122.5, 122.6, 123.8 (q, J = 275.9 Hz), 124.4, 125.0, 125.5, 126.7, 128.4, 130.2 (q, J = 5.8 Hz), 132.0, 132.4 (q, J = 30.7 Hz), 135.2, 135.3, 139.7, 154.0. ^{19}F NMR (376 MHz, $CDCl_3$) δ: -59.4. HRMS (DART) *m/z* calcd for $C_{23}H_{19}F_6O$ [M+H]+: 425.1340, found 425.1338.

Preparation of ZTA-261

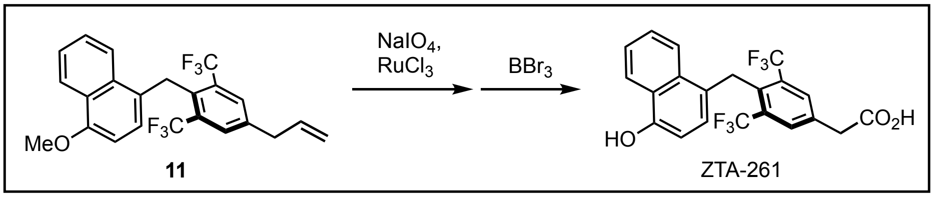

To a 10 mL flask 1-(4-allyl-2,6-bis(trifluoromethyl)benzyl)-4-methoxynaphthalene (112 mg, 0.26 mmol) and tetrabutylammonium iodide (9.4 mg, 0.026 mmol) were added followed by EtOAc (0.7 mL). Then, $RuCl_3{\cdot}xH_2O$ (2.7 mg, 0.014 mmol) was added and a solution of $NaIO_4$ (271 mg, 1.27 mmol) in water (2.1 mL) was added dropwise over 1 h at room temperature. The reaction mixture was stirred for an additional 4 h at room temperature. Water was added (10 mL) and the organic layer was extracted with EtOAc (3 x 10 mL), washed with brine, dried over sodium sulfate, and concentrated *in vacuo*. The crude material was dissolved in dry DCM (2.5 mL) and cooled to -78°C. Then, BBr_3 (0.76 mL, 0.76 mmol, 1M solution in DCM) was added dropwise and the reaction was stirred at -78°C for 1.5 h. The reaction was then warmed to room temperature and stirred for 45 minutes. Finally, the reaction was poured onto ice and the organic layer was extracted with EtOAc (3 x 10 mL), washed with brine, dried over sodium sulfate, and concentrated *in vacuo*. The crude material was purified by column chromatography (Toluene//EtOAc/AcOH = 20:1:1) and reverse-phase HPLC (Biotage SNAP Cartridge 12g) to give ZTA-261 as a white solid (13.8 mg, 13%). The purity of ZTA-261was determined to be >97% by HPLC analysis.

^{1}H NMR (400 MHz, $CDCl_3$) δ 3.85 (s, 2H), 4.70 (s, 2H), 6.13 (d, J = 8.0 Hz, 1H), 6.57 (d, J = 8.0 Hz, 1H), 7.55 (m, 1H), 7.63 (m, 1H), 7.92 (s, 2H), 8.09 (d, J = 8.4 Hz, 1H), 8.25 (d, J = 8.0 Hz, 1H). ^{13}C NMR (100 MHz, $CDCl_3$) δ 30.8, 40.2, 107.9, 122.4, 122.8, 123.6 (q, J = 279.5 Hz), 124.47, 124.51, 125.2, 126.9, 128.7 (q, J = 5.7 Hz), 132.3, 132.9 (q, J = 31.4 Hz), 133.0, 136.8, 150.1, 175.3. ^{19}F NMR (376 MHz, $CDCl_3$) δ: -59.2. HRMS (ESI) *m/z* calcd for $C_{20}H_{13}F_6O$ [M-CO_2H]-: 383.0865, found 383.0876.

HPLC chart of ZTA-261

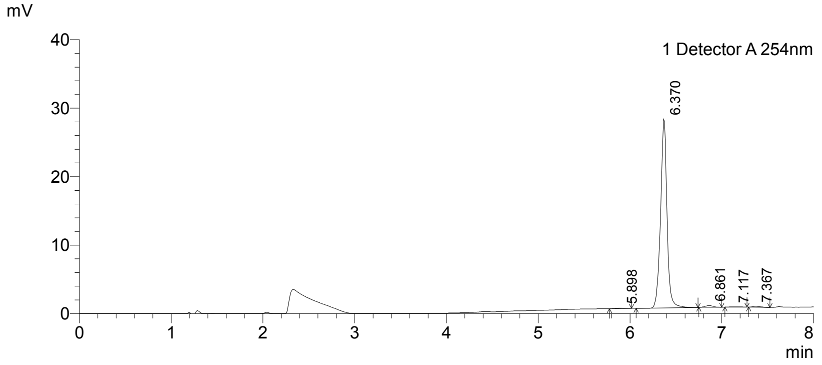

Peak Table

Detector A 254nm

Peak#	Ret. Time	Area	Height	Conc.	Unit	Mark	Name
1	5.898	358	57	0.261		M	
2	6.370	133886	27541	97.750		M	
3	6.861	1566	258	1.144		M	
4	7.117	421	56	0.307		M	
5	7.367	737	106	0.538		M	
Total		136968	28017				

Cell culture

GloResponse 9XGAL4UAS-luc2P HEK293 cells (Promega) were stably transfected with pFN26A(BIND) (Promega) in which the coding regions of the ligand binding domains of THRα or THRβ were inserted. Cells were cultured in DMEM supplemented with 10% FBS, 200 µg/mL Hygromycin and 750 µg/mL G418 in 90-mm cell culture dishes at 37°C with 5% CO_2. Cells were passaged every three days and used for luciferase reporter assay.

Luciferase reporter assay

Cells were suspended in assay medium (DMEM supplemented with 5% charcoal/dextran-treated FBS, 25 mM HEPES and 4mM L-Alanyl-L-glutamine Solution) and were seeded onto poly-L-lysine-coated 96-well plates at 2×10^4 cells / well. After incubation at 37°C and 5% CO_2 for 24 h, TH analog was added at the concentrations shown in Figure S1. On the next day, firefly luciferase luminescence was measured using the Dual-Glo Luciferase Assay System (Promega) using a microplate reader (SpectraMax i3, Molecular Devices). The luminescence of *Renilla* luciferase was used as an internal standard. EC_{50} values for each TH analogs were calculated by fitting the data to the Sigmoidal dose-response model using GraphPad Prism version 7.0 (GraphPad Software)

Evaluation of reference gene candidates for normalization of RT-qPCR analysis

Total RNA samples were extracted from frozen liver tissue of mice that were treated with either vehicle (n=9), 1 μmol/kg/day of T_3 (n=10), GC-1 (n=10), or ZTA-261 (n=10) using QIAzol reagent (Qiagen) according to the manufacturer's protocol. An equal amount of RNA from each group was mixed, and 1 μg of the mixture was used for cDNA synthesis with the ReverTra Ace qPCR RT Kit (TOYOBO). The qPCR reaction was performed in triplicates using QuantStudio 3 (Applied Biosystems) with a 20 μL reaction mixture containing 2 μL of cDNA, 10 μL of TB Green Premix Ex Taq™ II (TAKARA), 0.4 μL of ROX Dye Ⅱ (TAKARA), and 0.4 μM of each primer for reference gene candidates. The Mouse Housekeeping Gene Primer Set (TAKARA) was used for primers for *Atp5f1*, *B2m*, *Hprt1*, *Rplp1*, *Ppia*, *Rps18*, *Pgk1*, *Gusb*, *Tbp*, *Actb*, *Tfrc*, *Ywhaz*, and *Gapdh*. The sequence of the second set of *Gapdh* primers (Gapdh_2) was as follows: Forward: 5'-TGTGTCCGTCGTGGATCTGA-3', Reverse: 5'-CCTGCTTCACCACCTTCTTGAT-3. RefFinder[4, 5] software was used to analyze the results and select the optimal reference gene.

Blood-brain barrier permeability assay

The blood-brain barrier (BBB) transparency of TH analogs was assessed in vitro using RBT-24H (PharmaCo-Cell)[5] according to the manufacturer's protocol. Briefly, 10 μM of either GC-1 or ZTA-261 was added to the assay buffer (phosphate-buffered saline supplemented with 0.5 mM $MgCl_2$, 1 mM $CaCl_2$, and 4.5 mg/mL D-Glucose) in the wells mimicking the luminal side of the BBB, and allowed them to penetrate into the wells mimicking the abluminal side. Caffeine and digoxin were used as the positive and negative controls, respectively. After 30 min, the assay buffer in the wells mimicking the abluminal side was collected, and the concentration of the compounds was measured using liquid chromatography-mass spectrometry (LC-MS). Before injection into the LC-MS system, the samples were purified using a GL-tip GC (GL Science, Tokyo, Japan, for caffeine and digoxin) or GL-tip SDB (GL Science, Tokyo, Japan, for ZTA-261 and GC-1). These tips were preconditioned with 20 μL of acetonitrile and 20 μL of 0.1% TFA in purified water. 100 μL of the samples were loaded onto the preconditioned tips, washed with 200 μL of DW, and then eluted with 10 μL of acetonitrile. Triazine (1% vol/vol) was added to the eluent as an internal standard.

Chromatographic separation was performed using a Cadenza CD-C18 column (3 × 150 mm, 3 μm; Imtakt, Kyoto, Japan). All the experiments were performed using an Ultimate 3000 UHPLC system coupled with a Q Exactive Plus Orbitrap mass spectrometer (Thermo Fisher Scientific).

The injection volume was 10 μL for digoxin and 1 μL for other compounds. The mobile phase consisted of 100 % purified water (A) and 100 % acetonitrile (B). The following gradient elution program was used for chromatographic separation: 0–2 min (5 % B), 2–6 min (5–95 % B), 6–8 min (95 % B), 8–8.1 min (95–5 % B), 8.1–12 min (5 % B) at a flow rate of 0.4 mL/min. The temperature of the autosampler and the column was maintained at 10°C and 37°C, respectively.

The MS parameters were set as follows: sheath gas flow rate, 40 psi; Aux gas flow rate, 10; spray voltage, 4 kV; capillary temperature 350-degree; resolution 70,000, AGC target 3×10^6, Maximum IT 200 ms. Detection was performed in positive ion mode for triazine (m/z=216.10), caffeine (m/z=195.09) and in negative ion mode for digoxin (m/z=779.42), GC-1 (m/z=327.16), and ZTA-261 (m/z=383.09). Data were acquired and processed using the Xcalibur data system (Version 4.4, Thermo Fisher Scientific). The peak areas of the analytes were normalized to those of the internal standard.

Ranking Order (Better--Good--Average)

Method	1	2	3	4	5	6	7	8	9	10	11	12	13	14
Delta CT	B2m	Rplp1	Rps18	Tbp	Hptr1	Atp5f1	Ppia	Actb	Gusb	Tfrc	Pgk1	Gapdh	Gapdh_2	Ywhaz
BestKeeper	Hptr1	Atp5f1	Rps18	Rplp1	Tbp	B2m	Tfrc	Ppia	Actb	Gusb	Pgk1	Gapdh	Gapdh_2	Ywhaz
Normfinder	Actb	Pgk1	Gusb	B2m	Rplp1	Ppia	Tbp	Hptr1	Rps18	Atp5f1	Tfrc	Gapdh	Gapdh_2	Ywhaz
Genorm	Atp5f1 | Rps18		Rplp1	Hptr1	Tbp	B2m	Ppia	Actb	Gusb	Tfrc	Pgk1	Gapdh	Gapdh_2	Ywhaz
**Recommended comprehensive ranking**	**Rps18**	**Atp5f1**	**Rplp1**	**B2m**	**Hptr1**	**Actb**	**Tbp**	**Ppia**	**Gusb**	**Pgk1**	**Tfrc**	**Gapdh**	**Gapdh_2**	**Ywhaz**

Comprehensive Ranking | Delta CT | BestKeeper | Normfinder | Genorm

Genes Geomean of ranking values
Rps18 3.00
Atp5f1 3.31
Rplp1 3.31
B2m 3.46
Hptr1 3.56
Actb 4.90
Tbp 5.14
Ppia 6.96
Gusb 7.02
Pgk1 7.18
Tfrc 9.37
Gapdh 12.00
Gapdh_2 13.00
Ywhaz 14.00

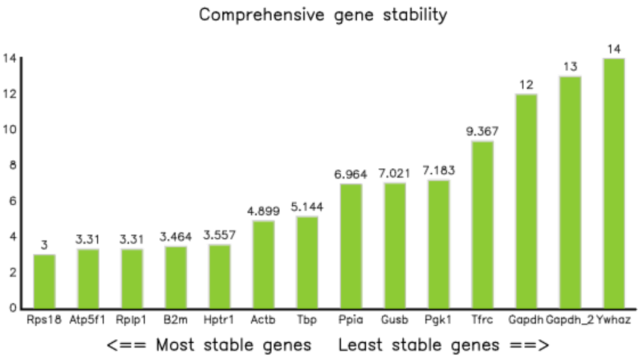

Supplementary Figure 1

Evaluation of reference gene candidates for normalization of RT-qPCR analysis

Fourteen primer sets for candidate genes were evaluated using RefFinder software[4, 5]. The Mouse Housekeeping Gene primer set (TAKARA) contained primers other than *Gapdh*_2. Gapdh_2 primer sets were shown in the supplementary methods section.

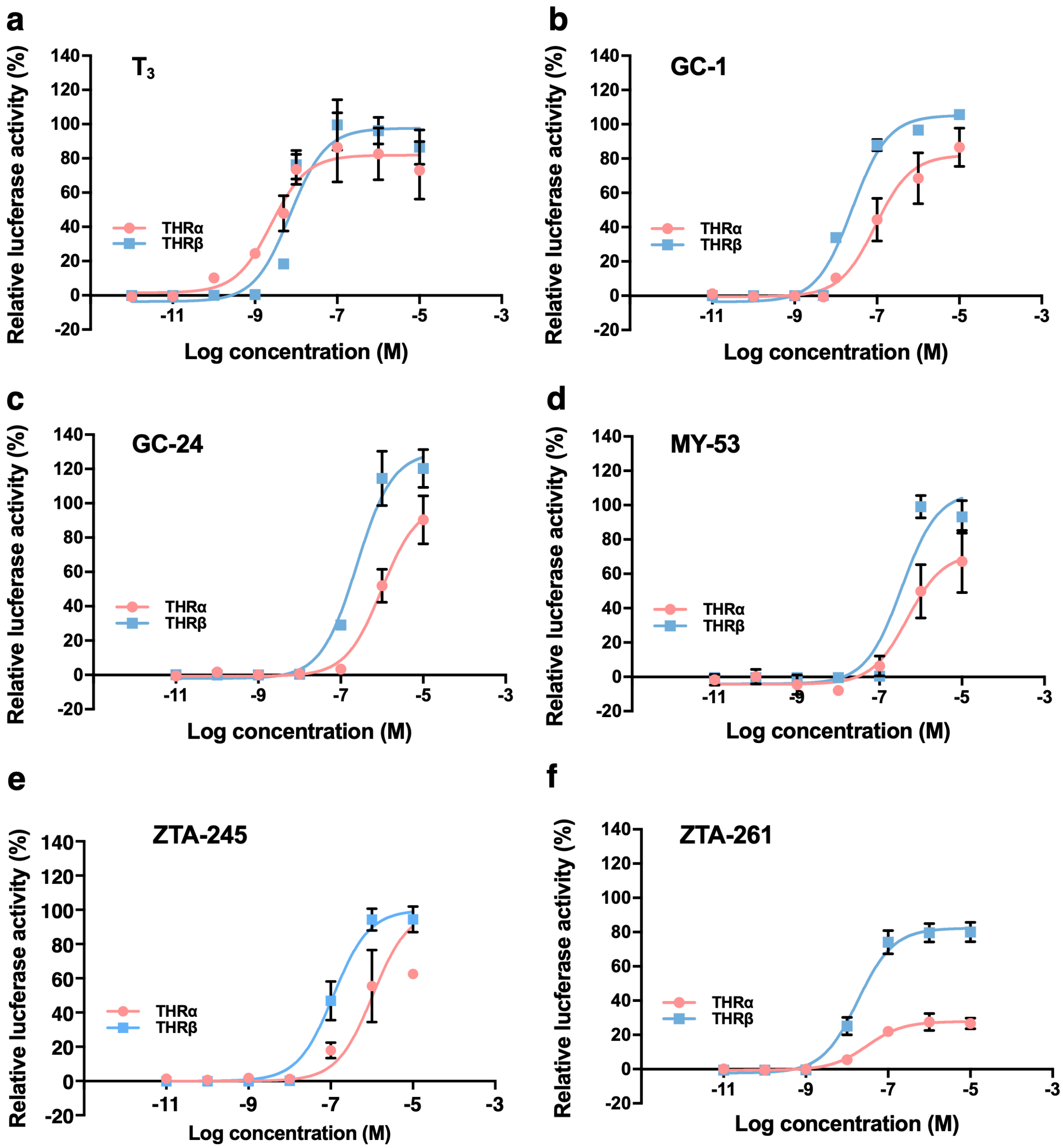

Supplementary Figure 2

Ligand binding assay using 9×GAL4UAS-*luc2P* reporter cell lines that express ligand binding domain of THRs.

HEK293 cells containing 9×GAL4UAS-*luc2P* reporter that stably express ligand binding domain (LBD) of either THRα or THRβ as a GAL4 DBD fusion protein were incubated in the presence of T_3 (A), GC-1 (B), GC-24 (C) MY-53(D), ZTA-245 (E), and ZTA-261(F) at the concentrations indicated in the figures. After incubation, cells were lysed and subjected to luminescence measurement. Luciferase activities are shown as relative values (mean ± SD, n=3) normalized against the maximum luminescence counts of THRβ LBD-expressing cell in the presence of T_3, which was set to 100%. Data were fitted with a sigmoidal dose-response model.

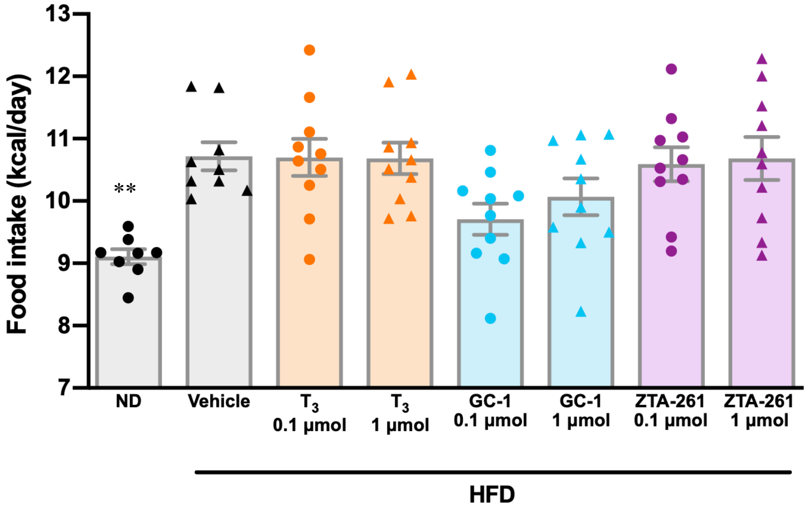

Supplementary Figure 3

Food consumption of mice during the course of the experiment.

Male 8-weeks-old C57BL/6J mice were fed with either a normal diet (ND; 10 kcal% fat, D12450B, RESEARCH DIETS) or a high-fat diet (HFD; 60 kcal% fat, D12492, RESEARCH DIETS). After 8 weeks of rearing with the ND or HFD, the animals were intraperitoneally injected with either, T_3 (0.1 or 1 µmol/kg·day) (A), GC-1 (0.1 or 1 µmol/kg·day) (B) or ZTA-261 (0.1 or 1 µmol/kg·day) (C) for 3 weeks. Food consumption was measured once every week, and average values of calory intake over the 11 weeks of the experiment were plotted. Data are shown as mean ± SEM (n=8-10, $^{**}P < 0.01$ vs Vehicle by one-way ANOVA with Dunnett's post hoc analysis). A significant difference was observed between the vehicle- and ND-fed groups. No significant differences were observed between the vehicle- and compound-treated groups fed with HFD.

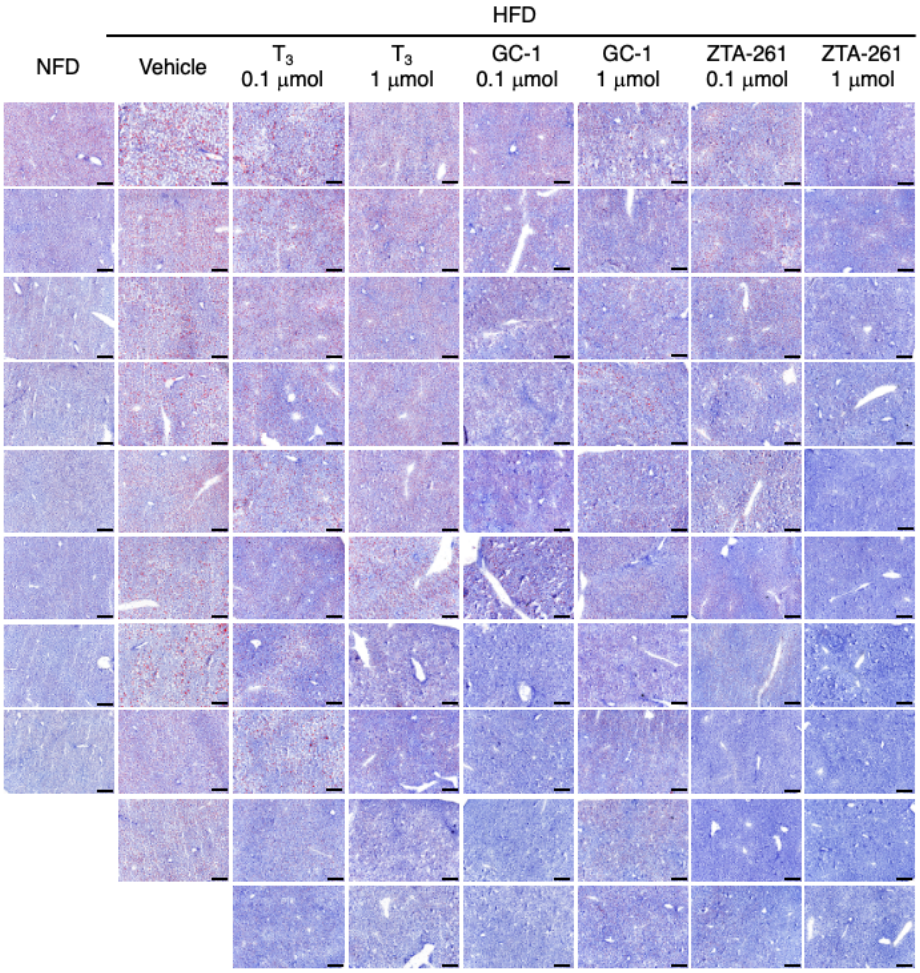

Supplementary Figure 4
Oil Red O staining of the liver sections
The liver sections were prepared from mice fed with either a normal diet (n = 8) or a high-fat diet injected with either saline (n = 9), T_3 (0.1 or 1 µmol/kg BW/day, n = 10), GC-1 (0.1 or 1 µmol/kg/day, n = 10), or ZTA-261 (0.1 or 1 µmol/kg/day, n = 10), and subjected to Oil Red O staining (scale bar = 200 µm). A representative image from each mouse was shown.

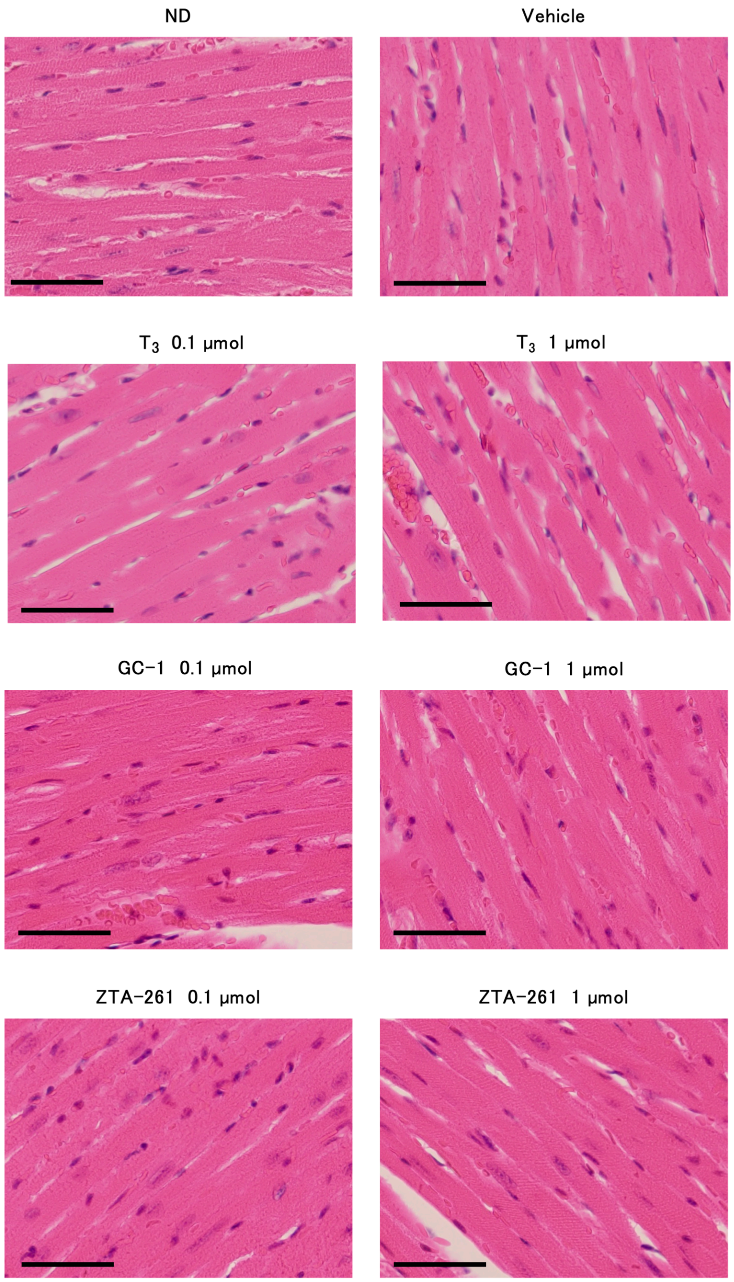

Supplementary Figure 5
Hematoxylin-eosin staining of the heart sections
The heart was collected from mice fed with either a normal diet or a high-fat diet injected with either saline, T_3 (0.1 or 1 μmol/kg BW/day), GC-1 (0.1 or 1 μmol/kg/day), or ZTA-261 (0.1 or 1 μmol/kg/day), sections were prepared and subjected to hematoxylin-eosin staining (scale bar = 50 μm).

Supplementary Tables

Supplementary Table 1 EC_{50} values of compounds

Compounds	hTHRα		hTHRβ	
	EC_{50} (nM)	95% CI (nM)	EC_{50} (nM)	95% CI (nM)
T_3	2.5	1.3 to 4.7	7.1	4.1 to 12
GC-1	92	53 to 1.6×10^2	25	18 to 36
GC-24	9.8×10^2	6.5×10^2 to 1.5×10^3	2.5×10^2	1.6×10^2 to 3.8×10^2
MY-53	4.7×10^2	2.1×10^2 to 1.1×10^3	3.5×10^2	1.6×10^2 to 7.7×10^2
ZTA-245	1.0×10^3	5.8×10^2 to 1.9×10^3	1.2×10^2	89 to 1.5×10^2
ZTA-261	31	18 to 52	18	14 to 25

Supplementary Table 2 in vitro blood-brain barrier transparency assay

	Digoxin	Caffeine	GC-1	ZTA-261
Abluminal concentration (μM)	B.L.Q	0.504 ± 0.0551	0.0966 ± 0.0234	0.0481 ± 0.0168
Apparent permeability (P_{app}, 10^{-6} cm/s)	N.D.	76.4 ± 8.35	14.6 ± 3.54	7.28 ± 2.55

The apparent blood-brain barrier permeability was calculated from the concentration of each compound detected in the medium on the abluminal side using equation (1). Data were shown as mean ± standard deviation (n=3). B.L.Q; Below the limit of quantification. N.D.; Not determined.

^{1}H, ^{13}C and ^{19}F NMR Spectra of Products

^{1}H-NMR (400 MHz, $CDCl_3$) of **1**

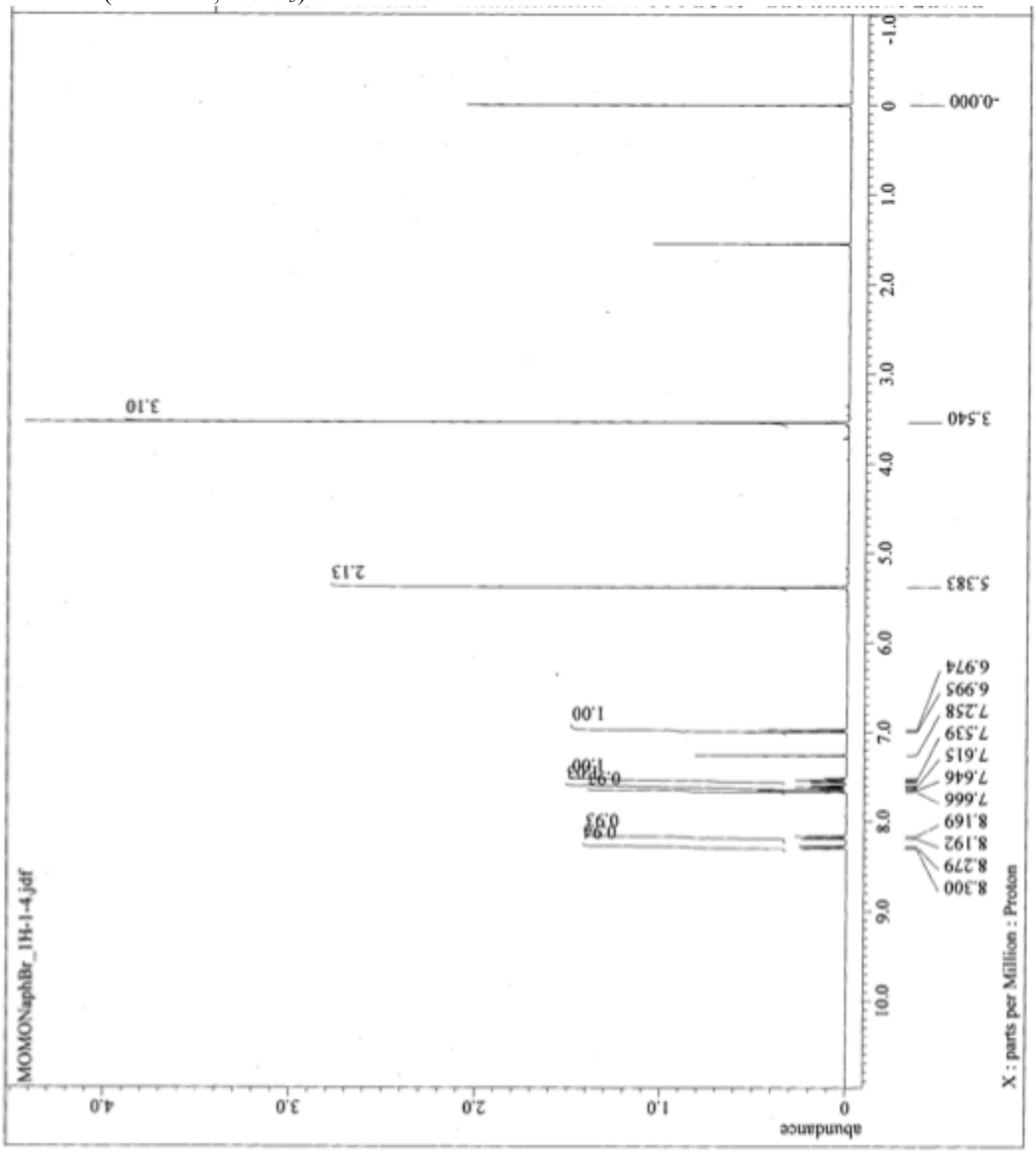

^{13}C-NMR (100 MHz, $CDCl_3$) of **1**

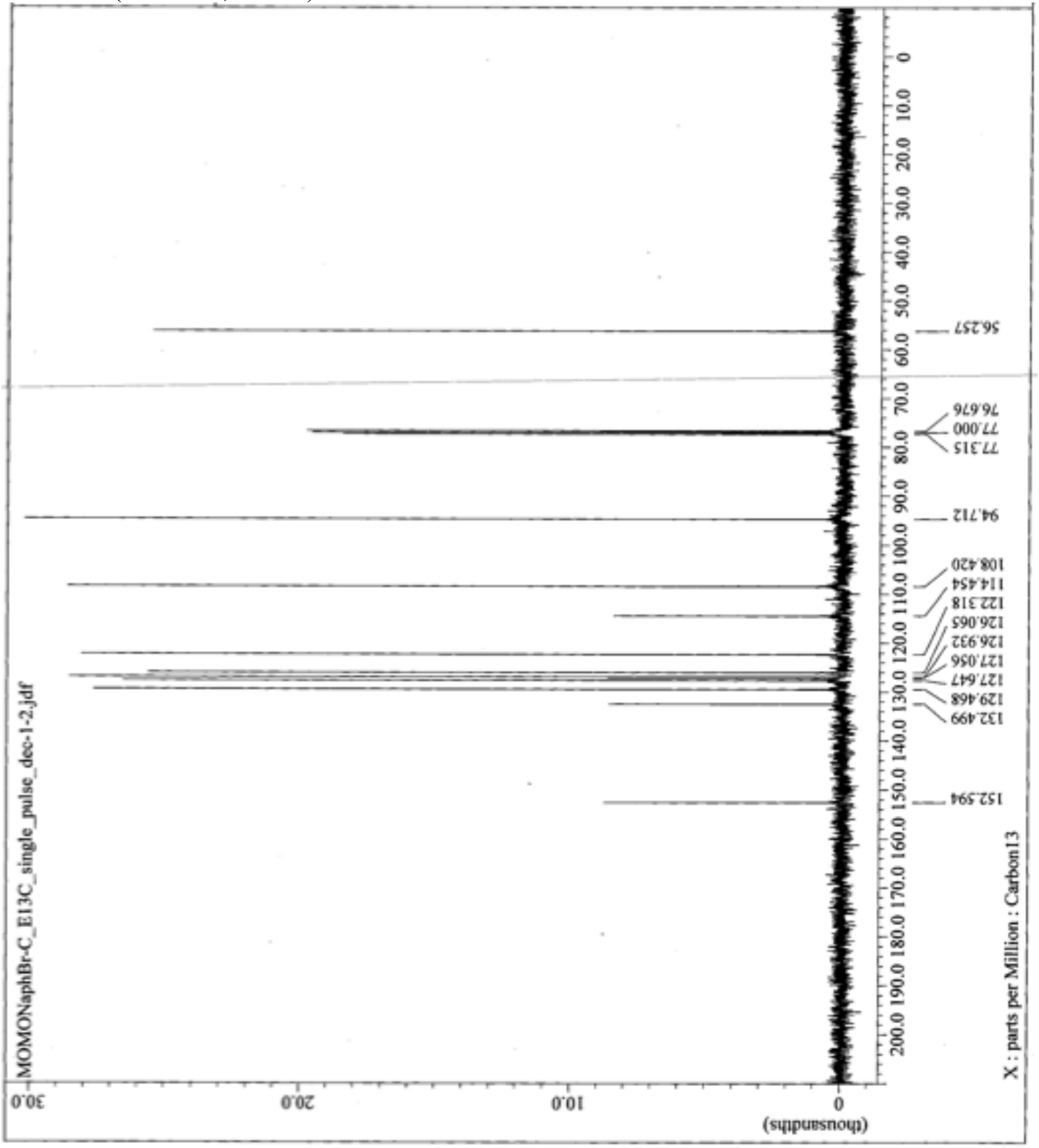

^{1}H-NMR (600 MHz, $CDCl_3$) of **2**

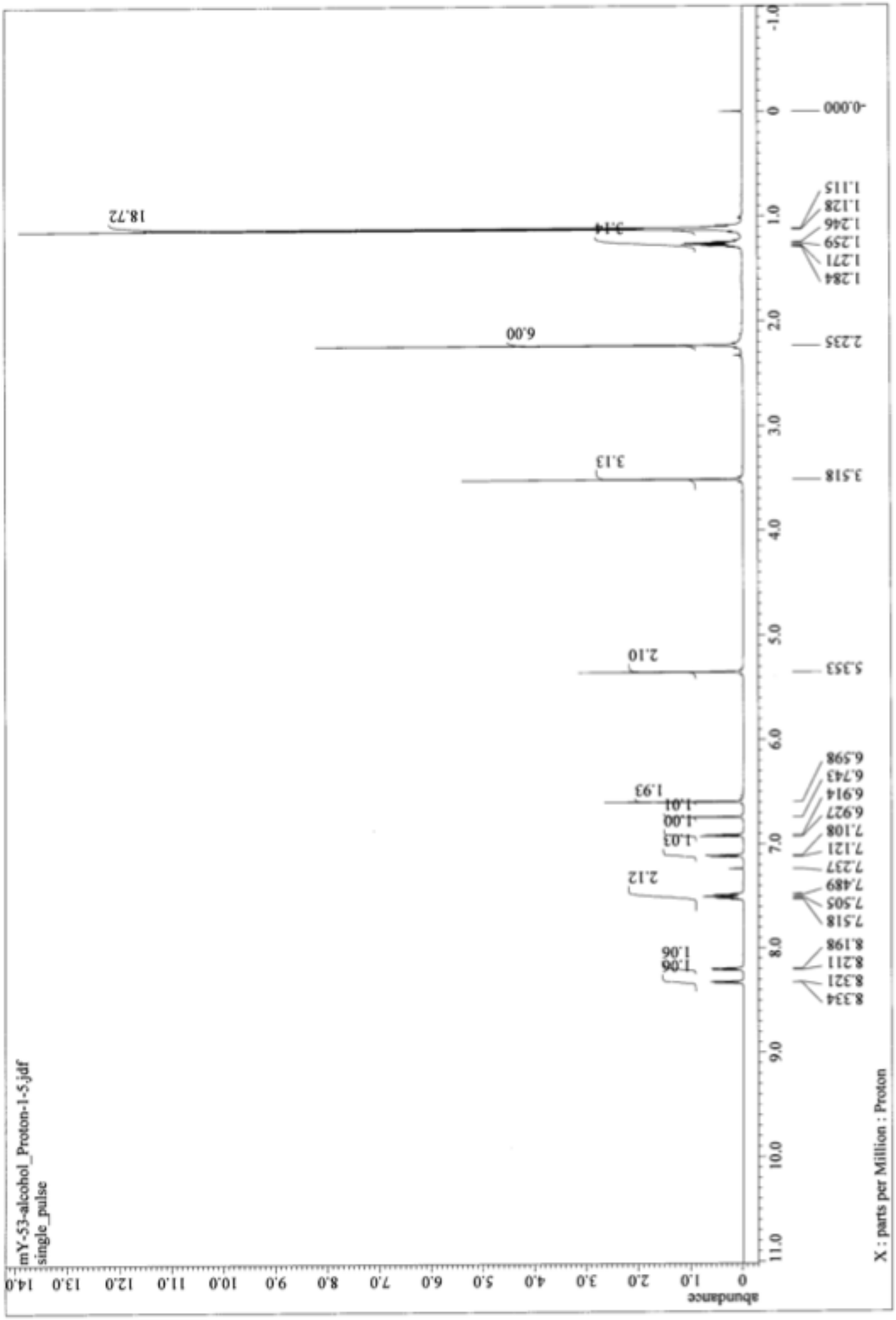

^{13}C-NMR (150 MHz, $CDCl_3$) of **2**

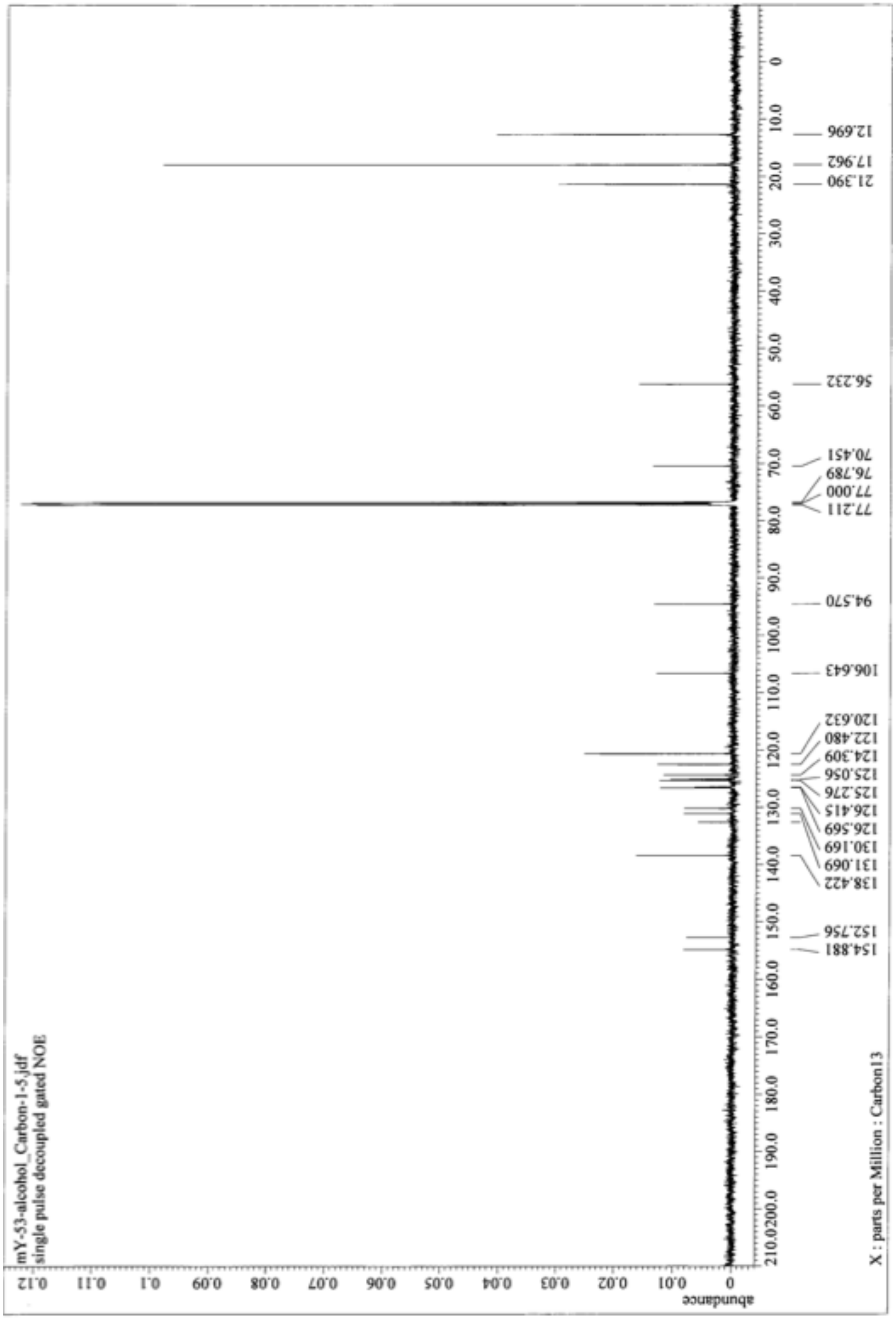

^{1}H-NMR (600 MHz, $CDCl_3$) of **3**

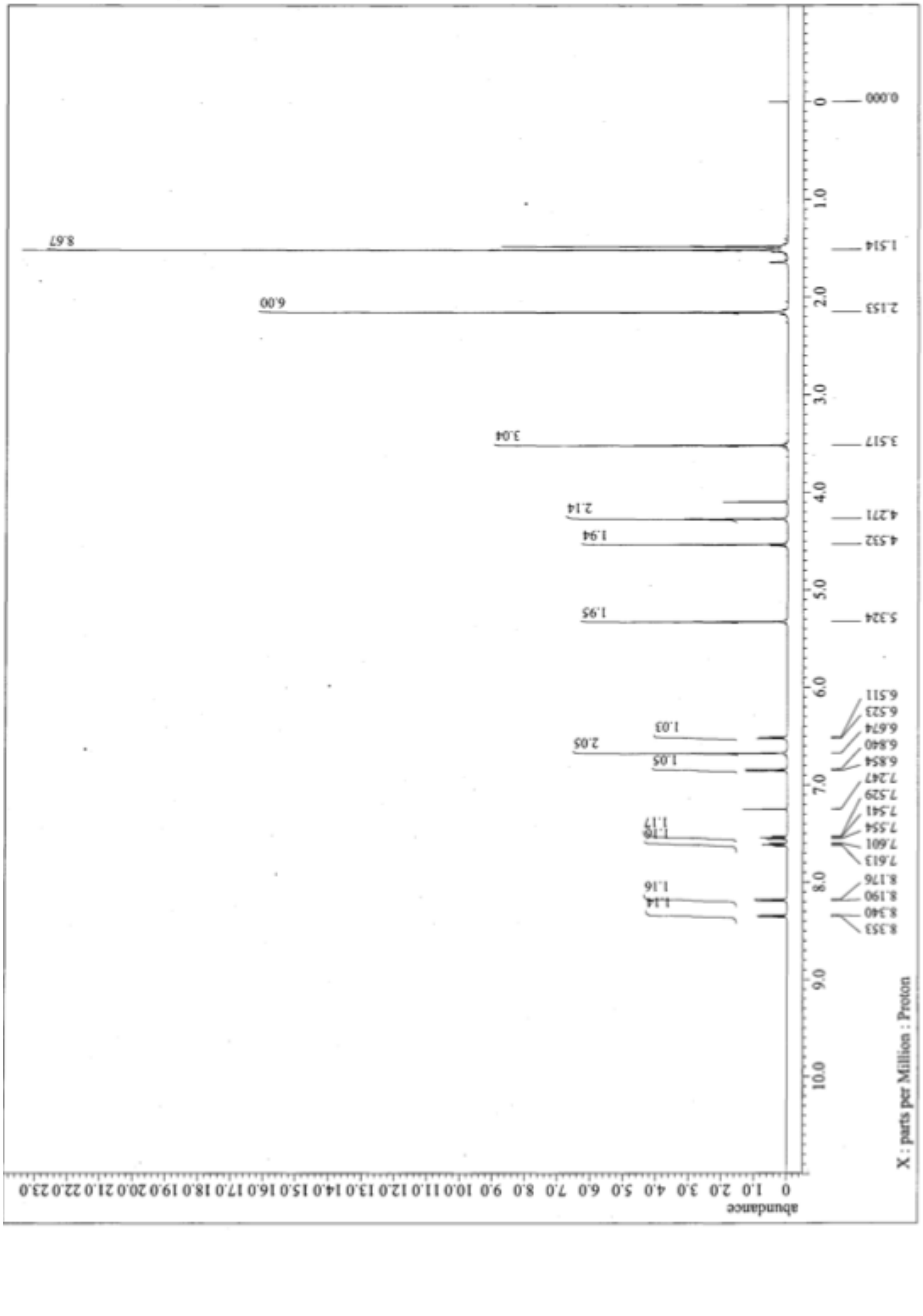

^{13}C-NMR (150 MHz, $CDCl_3$) of **3**

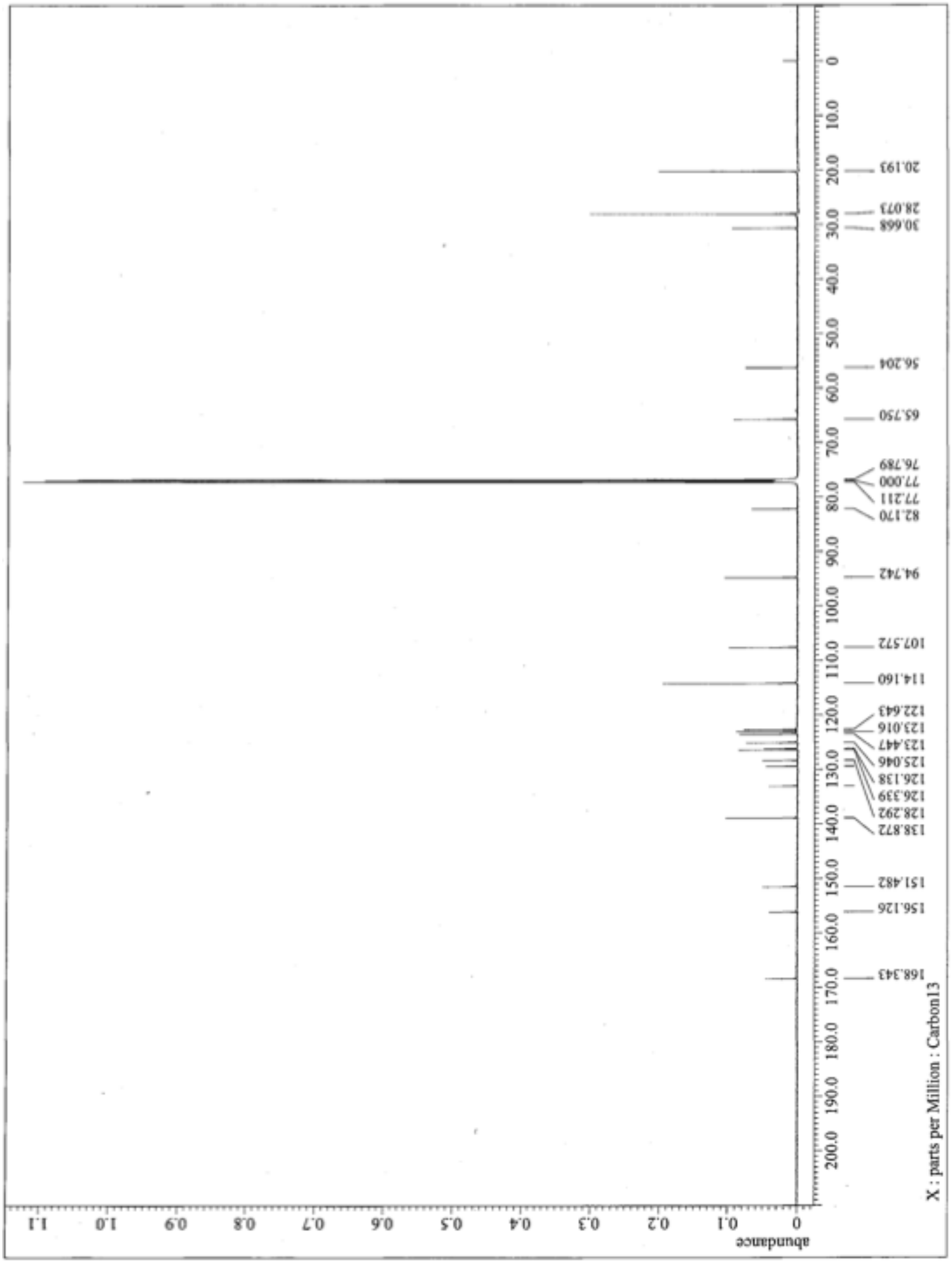

^{1}H-NMR (400 MHz, CD_3OD) of MY-53

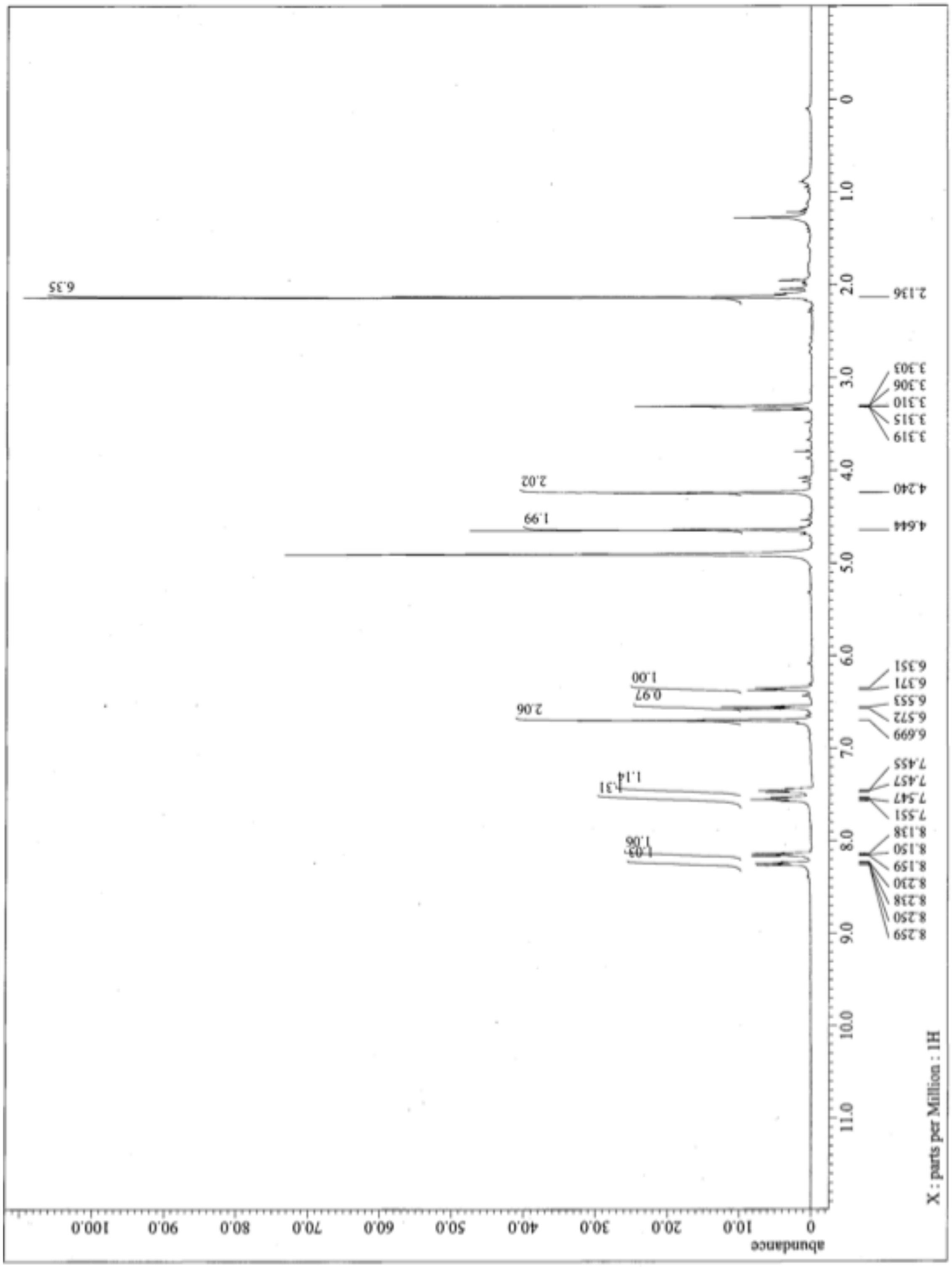

^{13}C-NMR (150 MHz, CD_3OD) of MY-53

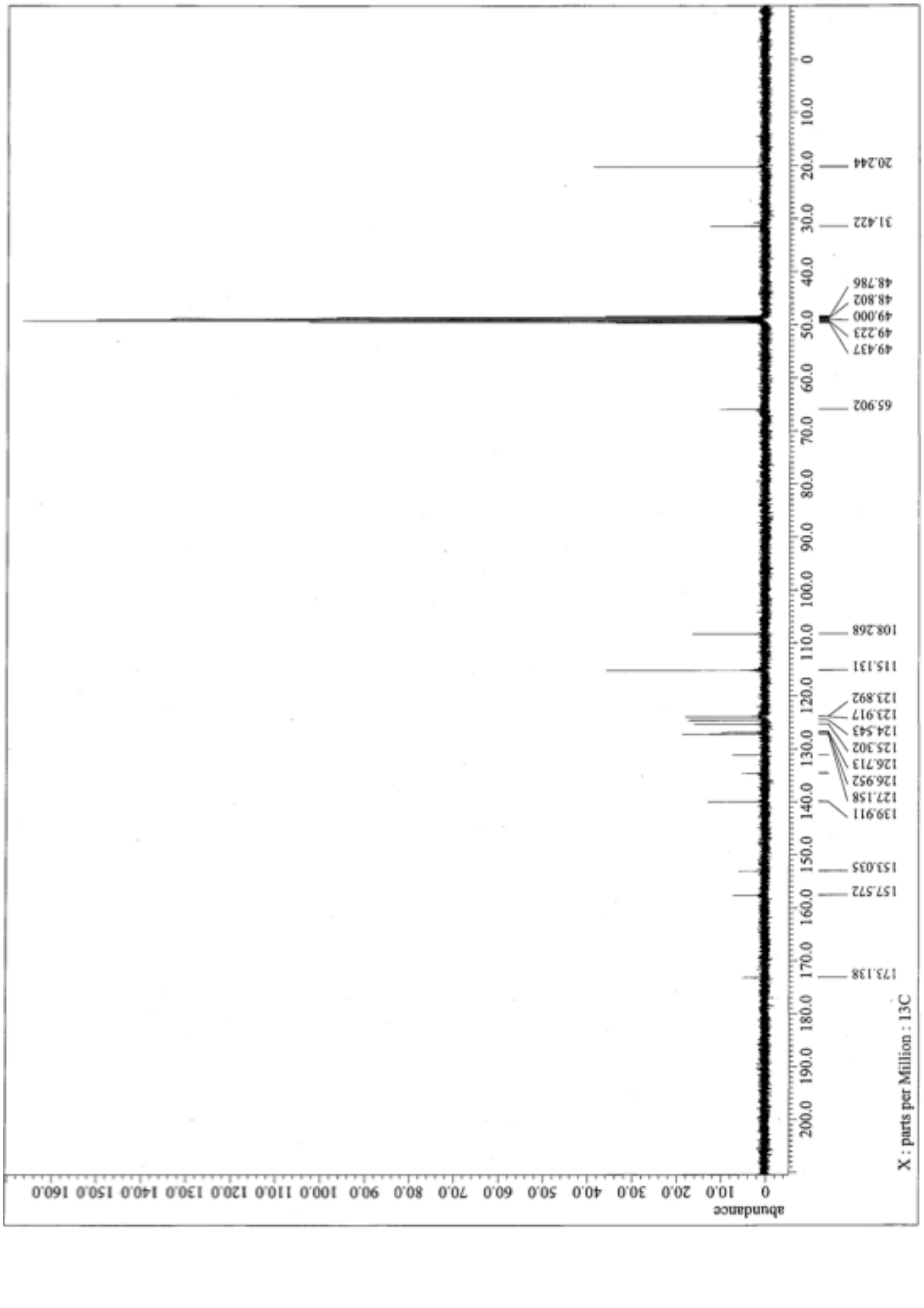

^{1}H-NMR (400 MHz, $CDCl_3$) of **4**

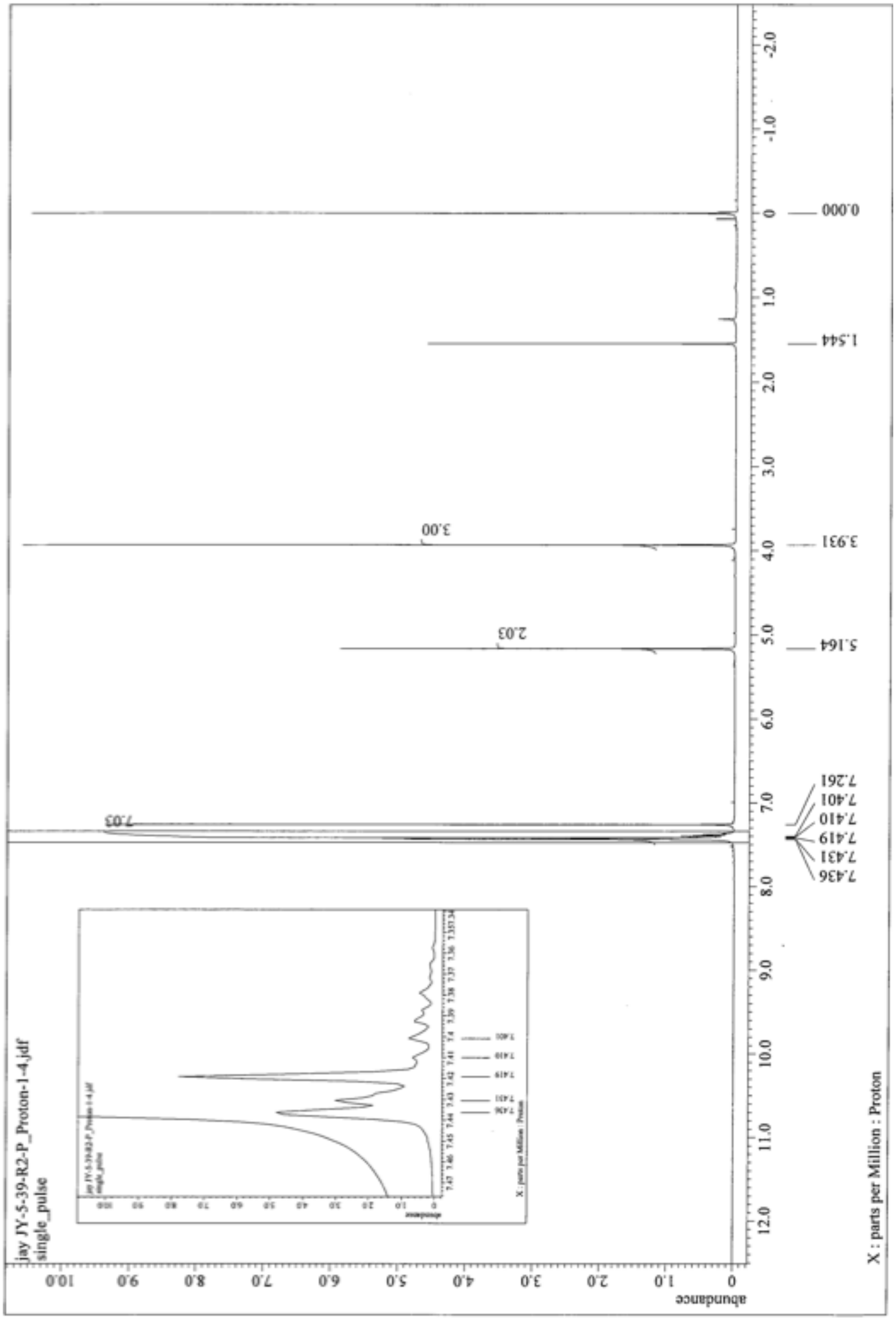

^{13}C-NMR (100 MHz, $CDCl_3$) of **4**

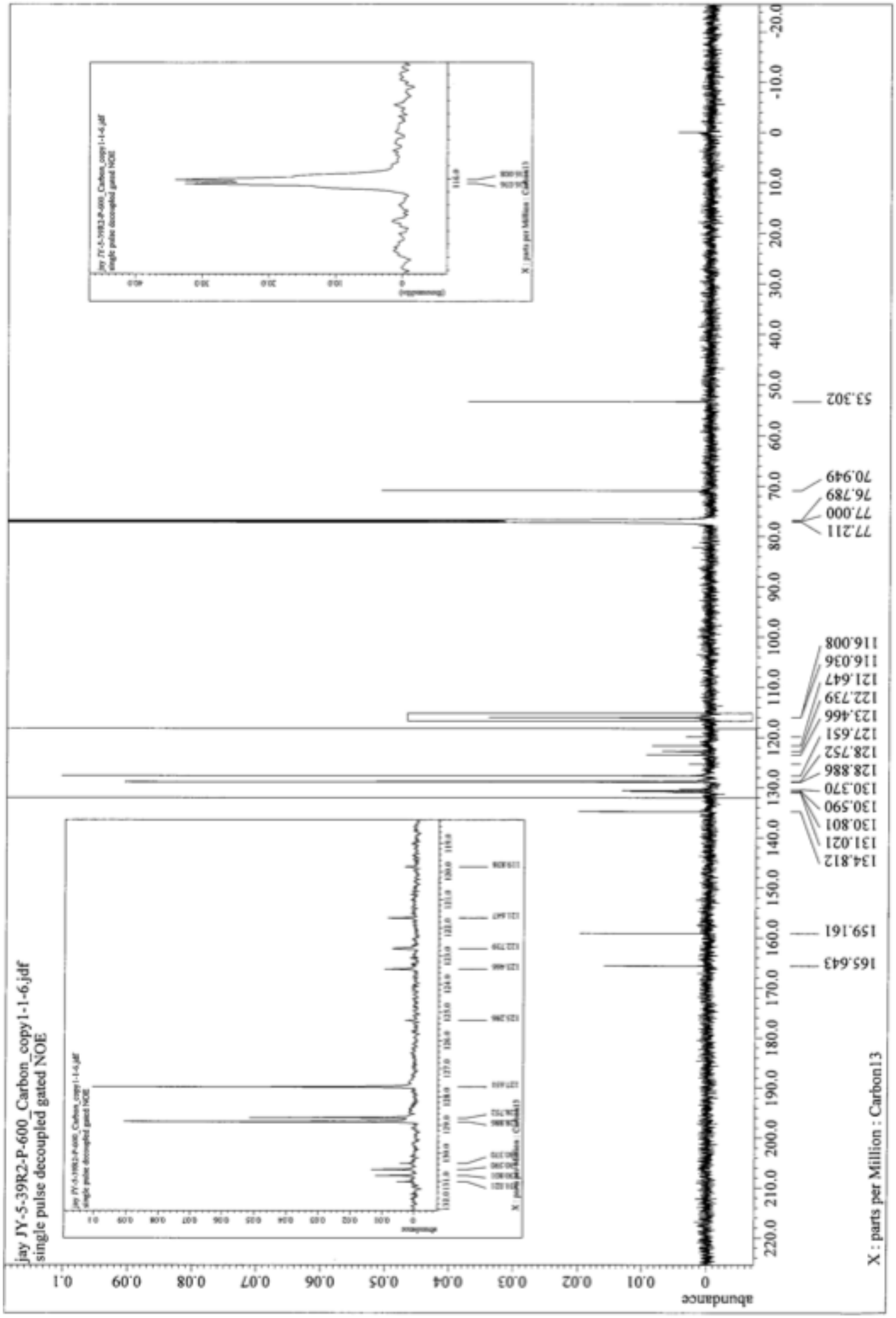

^{19}F-NMR (376 MHz, $CDCl_3$) of **4**

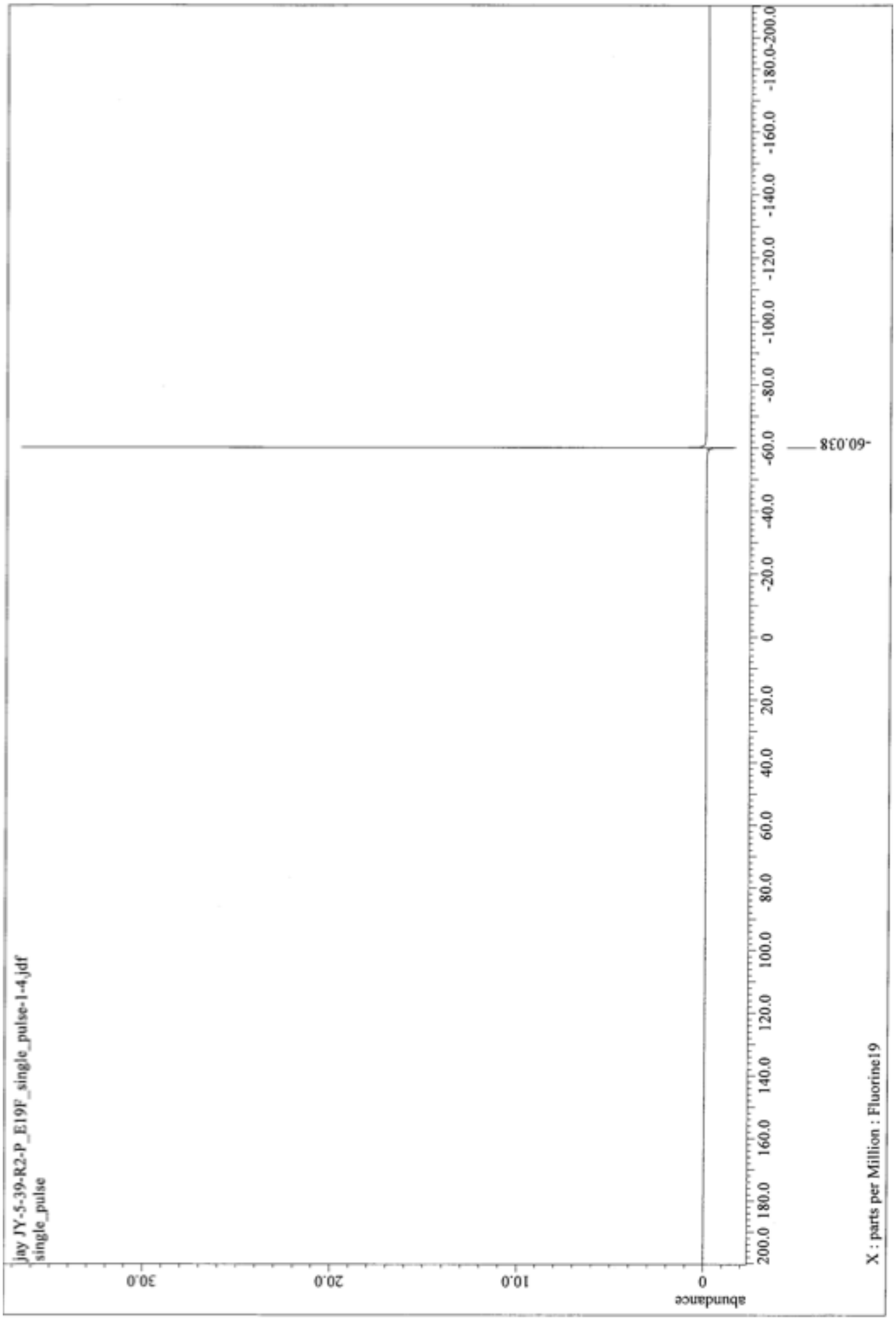

^{1}H-NMR (400 MHz, $CDCl_3$) of **5**

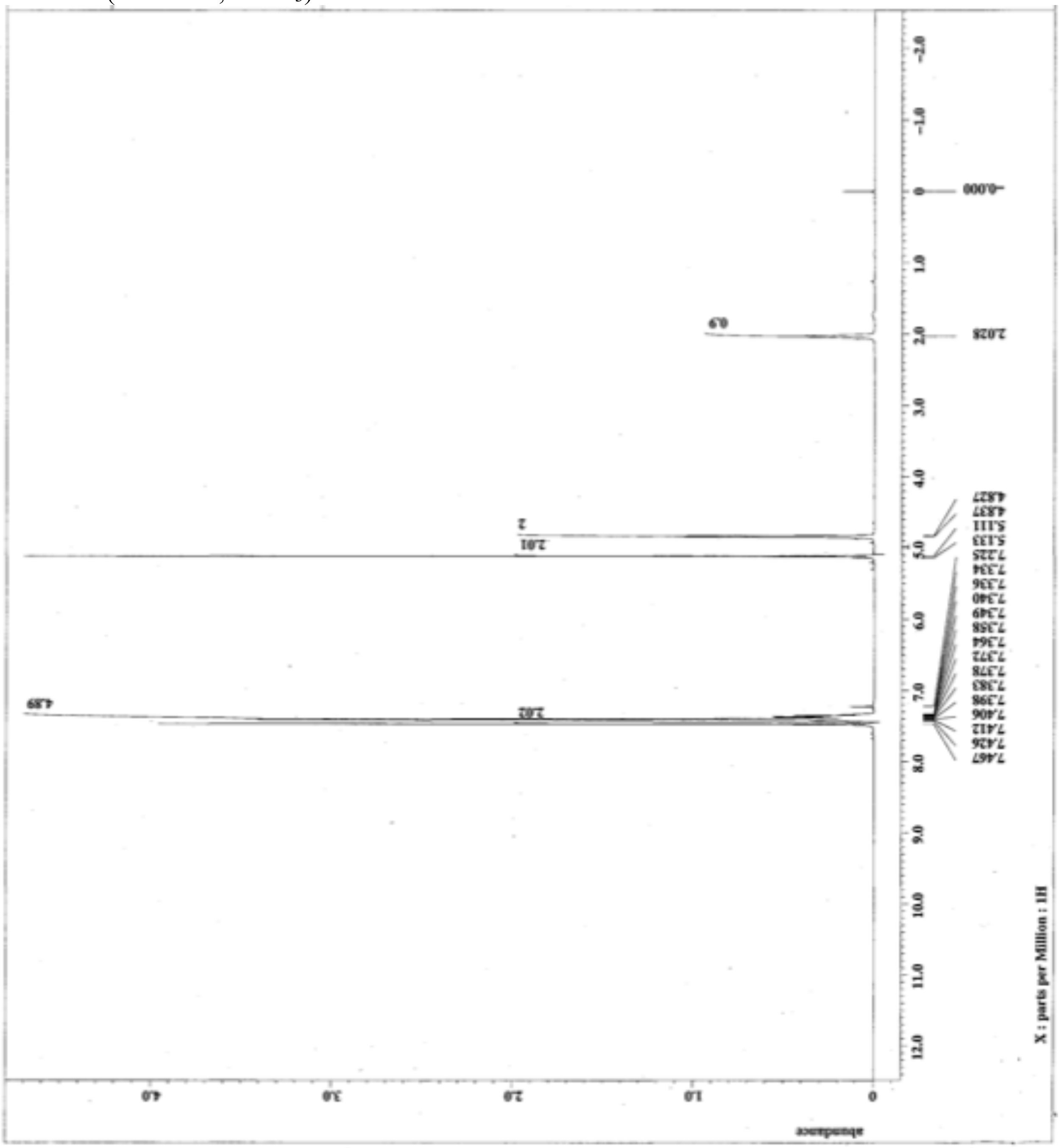

^{13}C-NMR (100 MHz, $CDCl_3$) of **5**

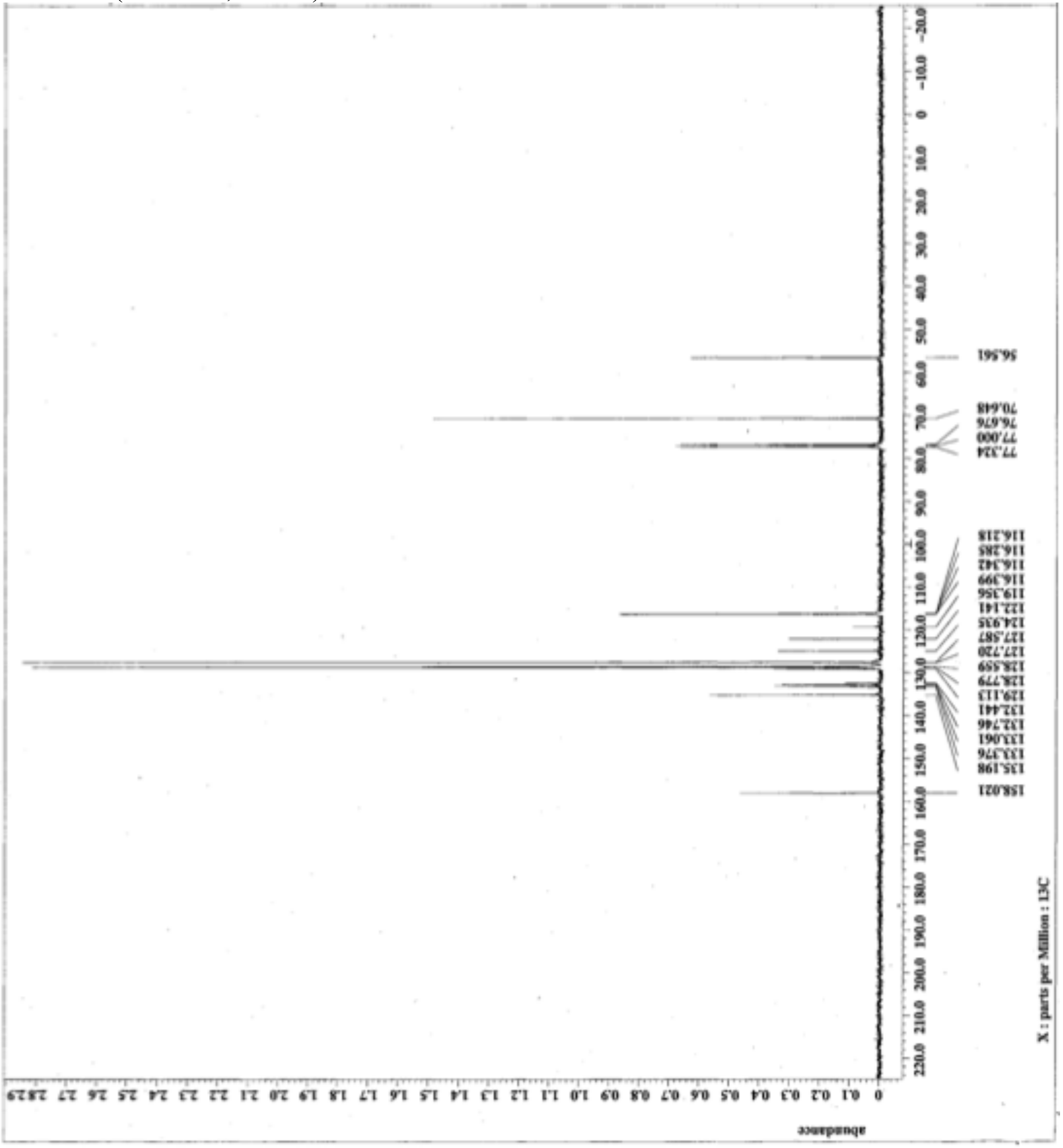

^{19}F-NMR (376 MHz, $CDCl_3$) of **5**

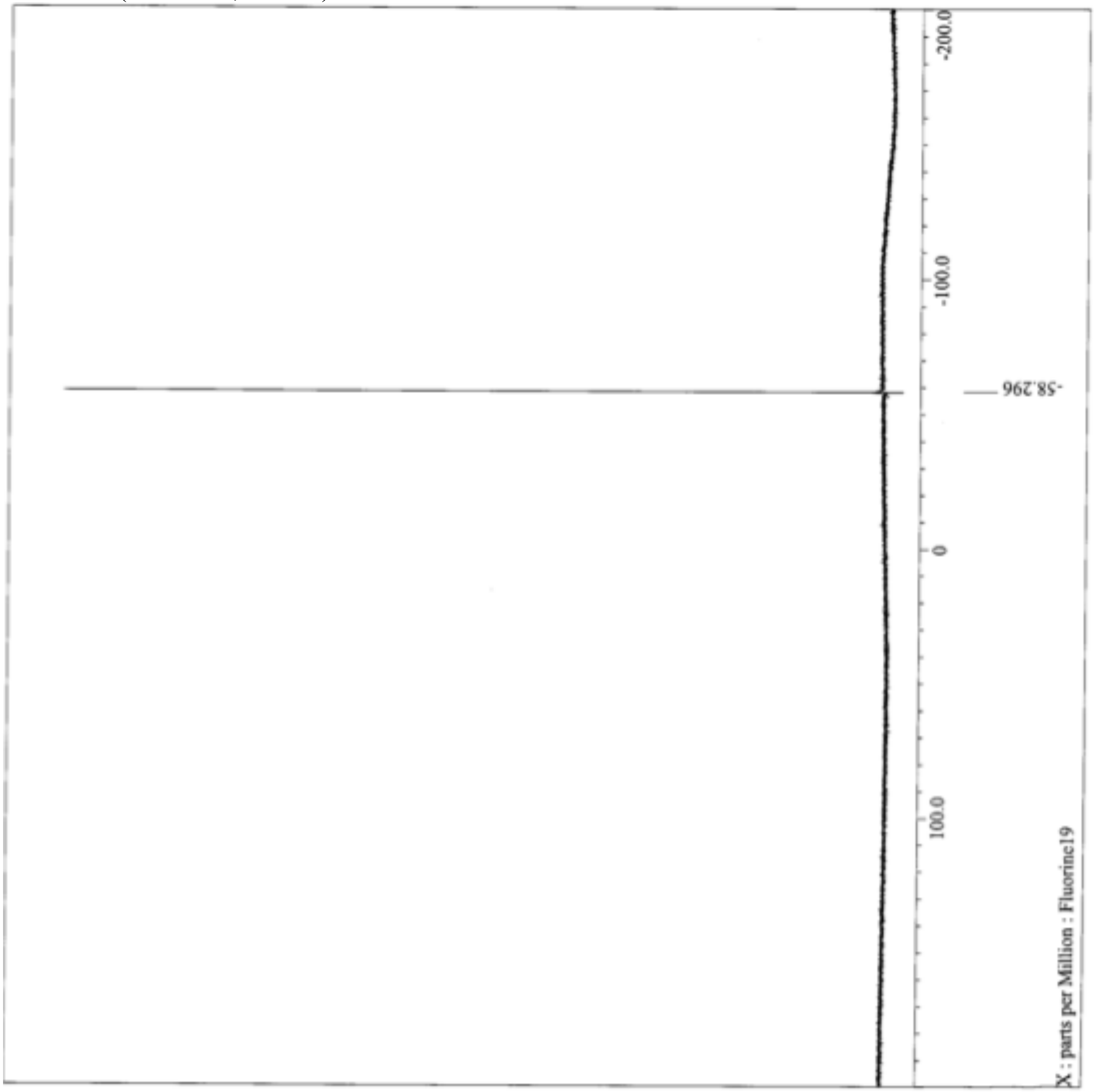

^{1}H-NMR (400 MHz, CDCl$_3$) of **6**

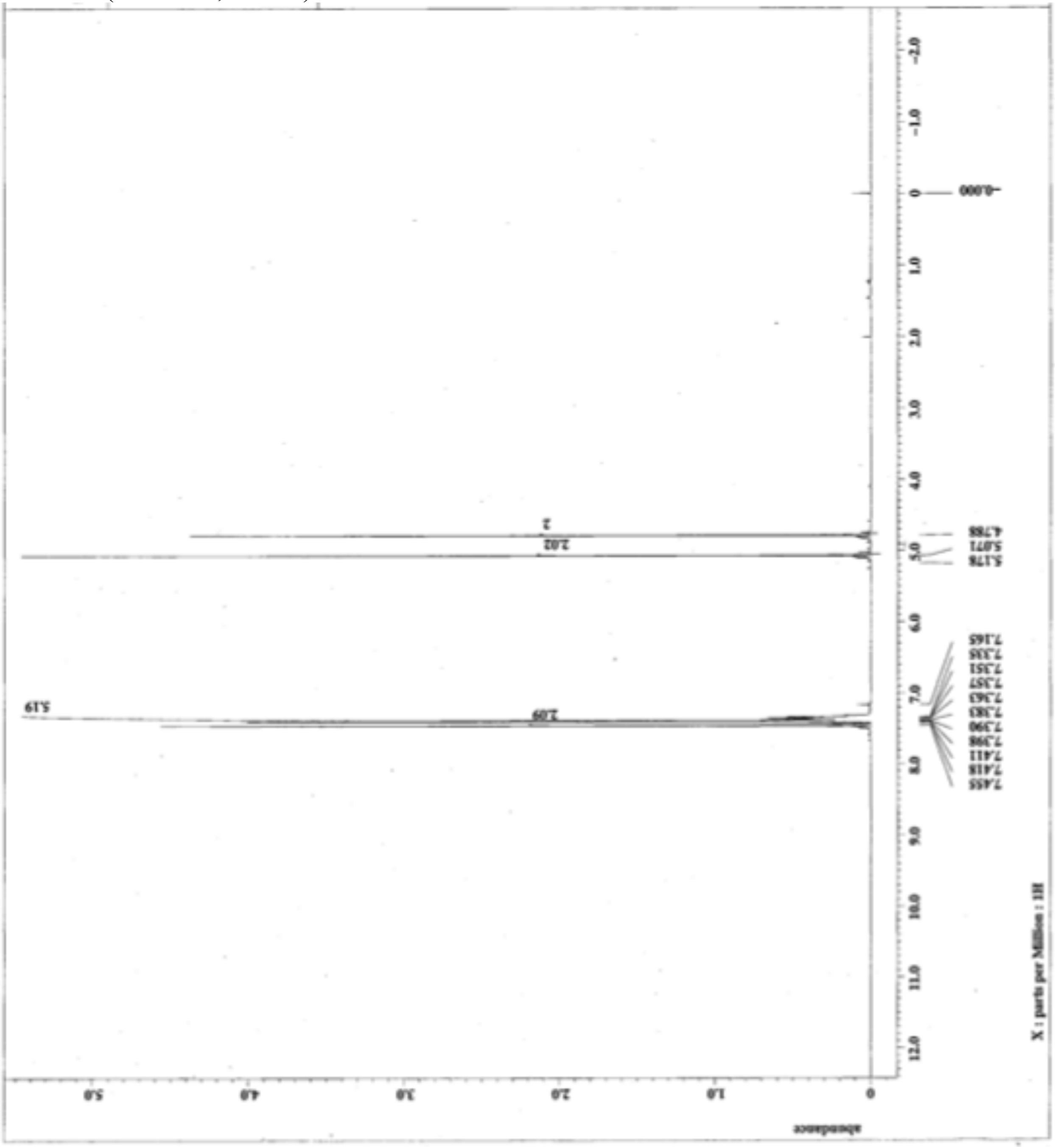

^{13}C-NMR (100 MHz, $CDCl_3$) of **6**

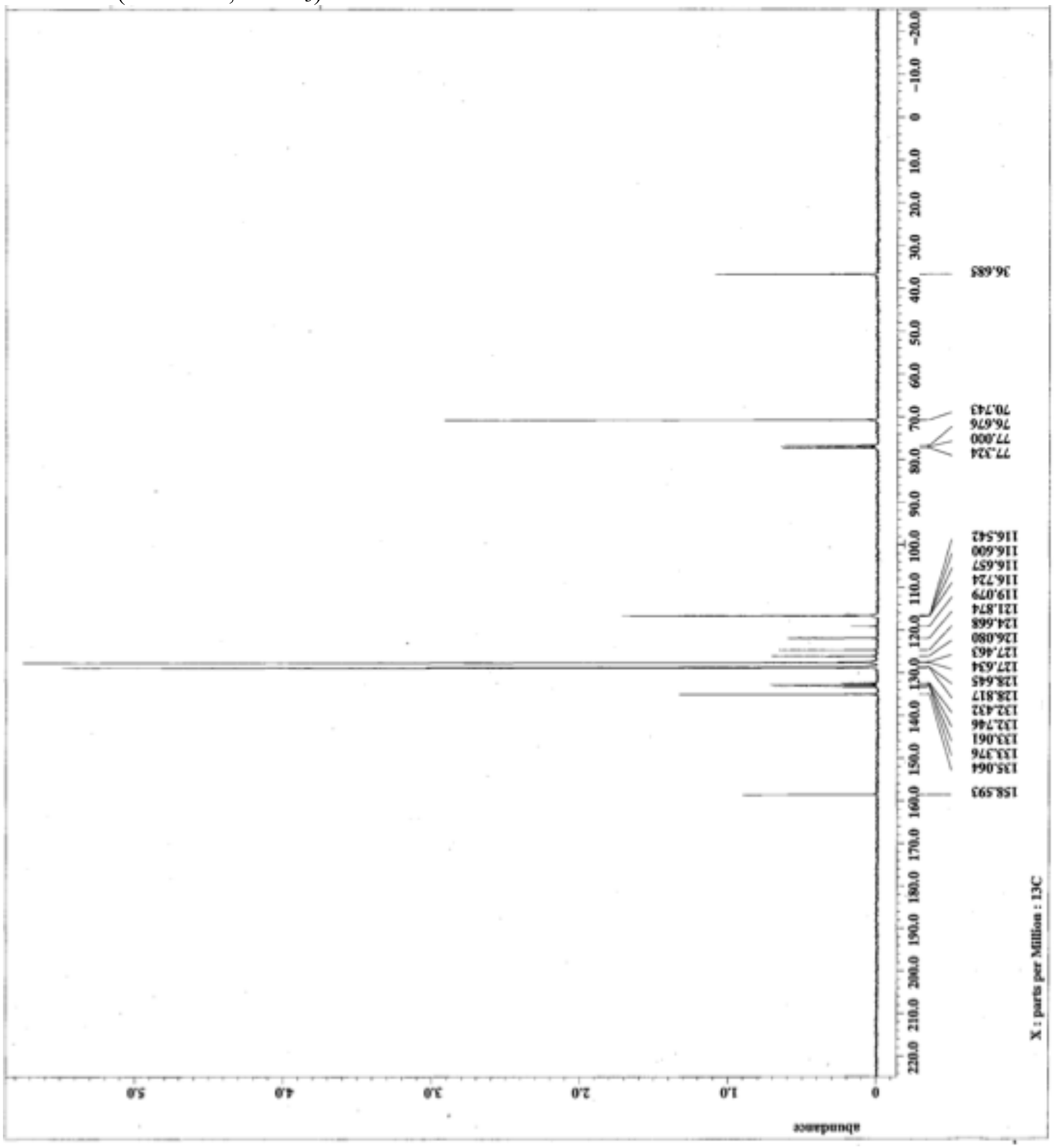

^{19}F-NMR (376 MHz, $CDCl_3$) of **6**

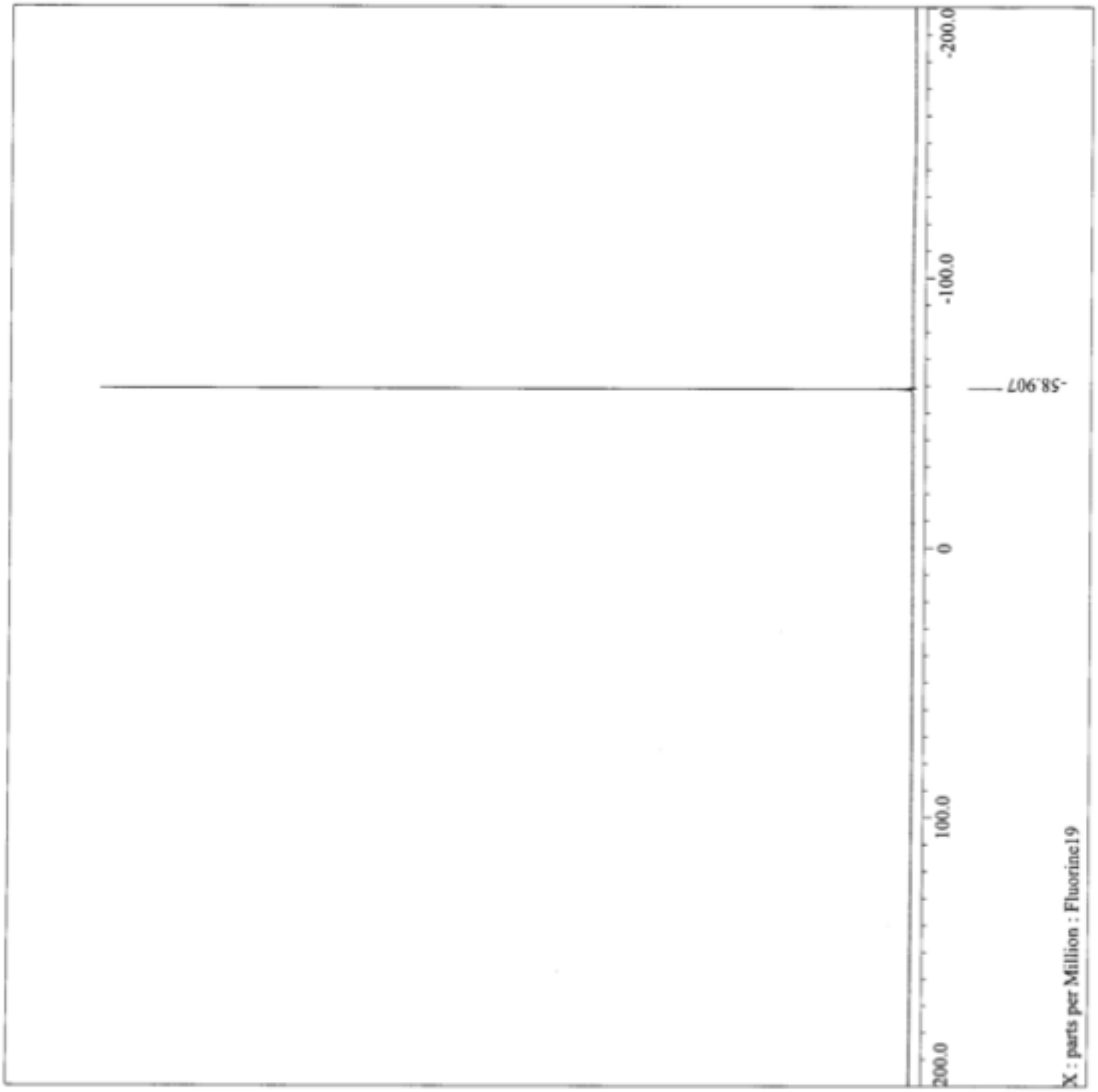

^{1}H-NMR (400 MHz, $CDCl_3$) of **7**

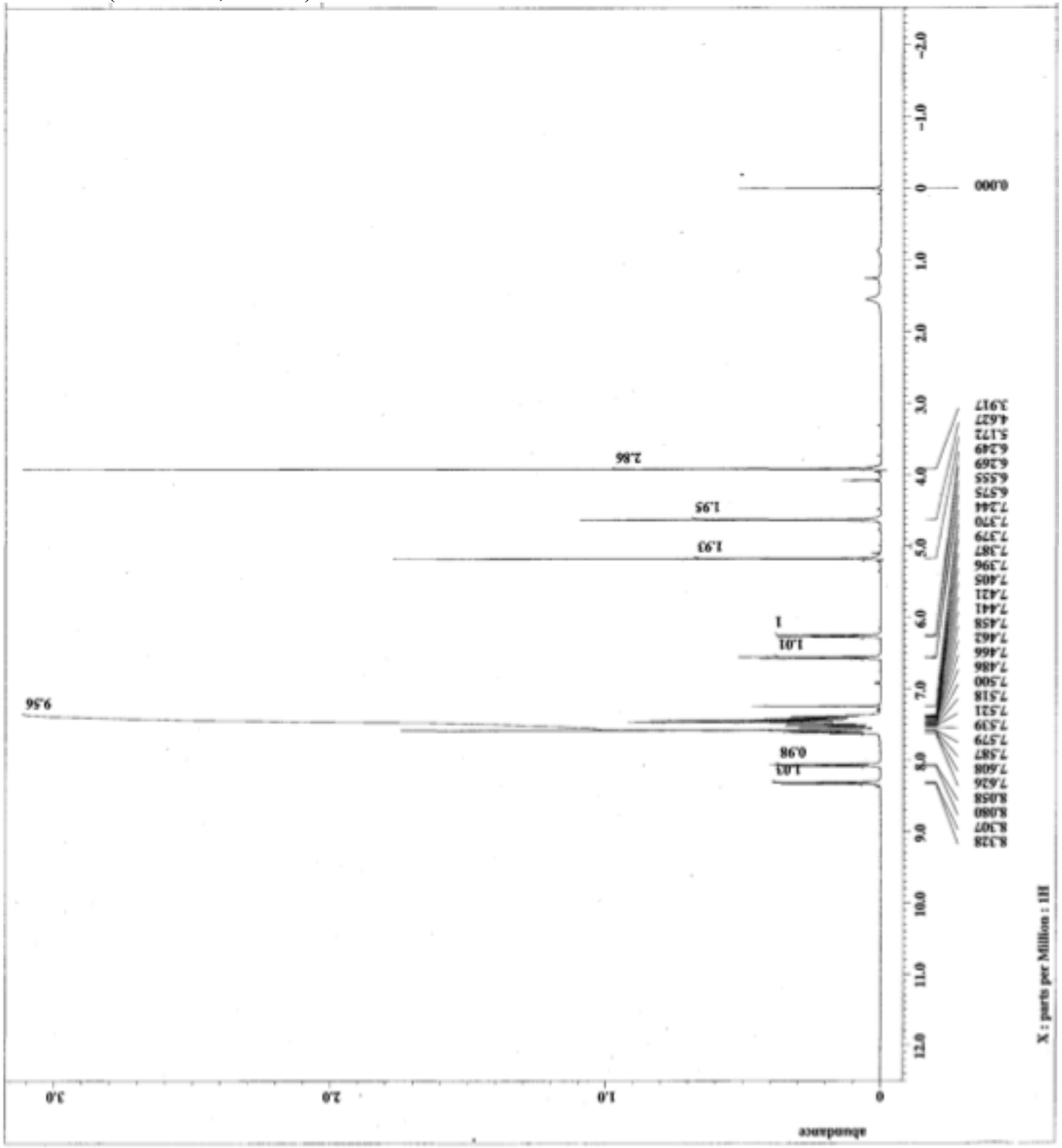

^{13}C-NMR (100 MHz, $CDCl_3$) of **7**

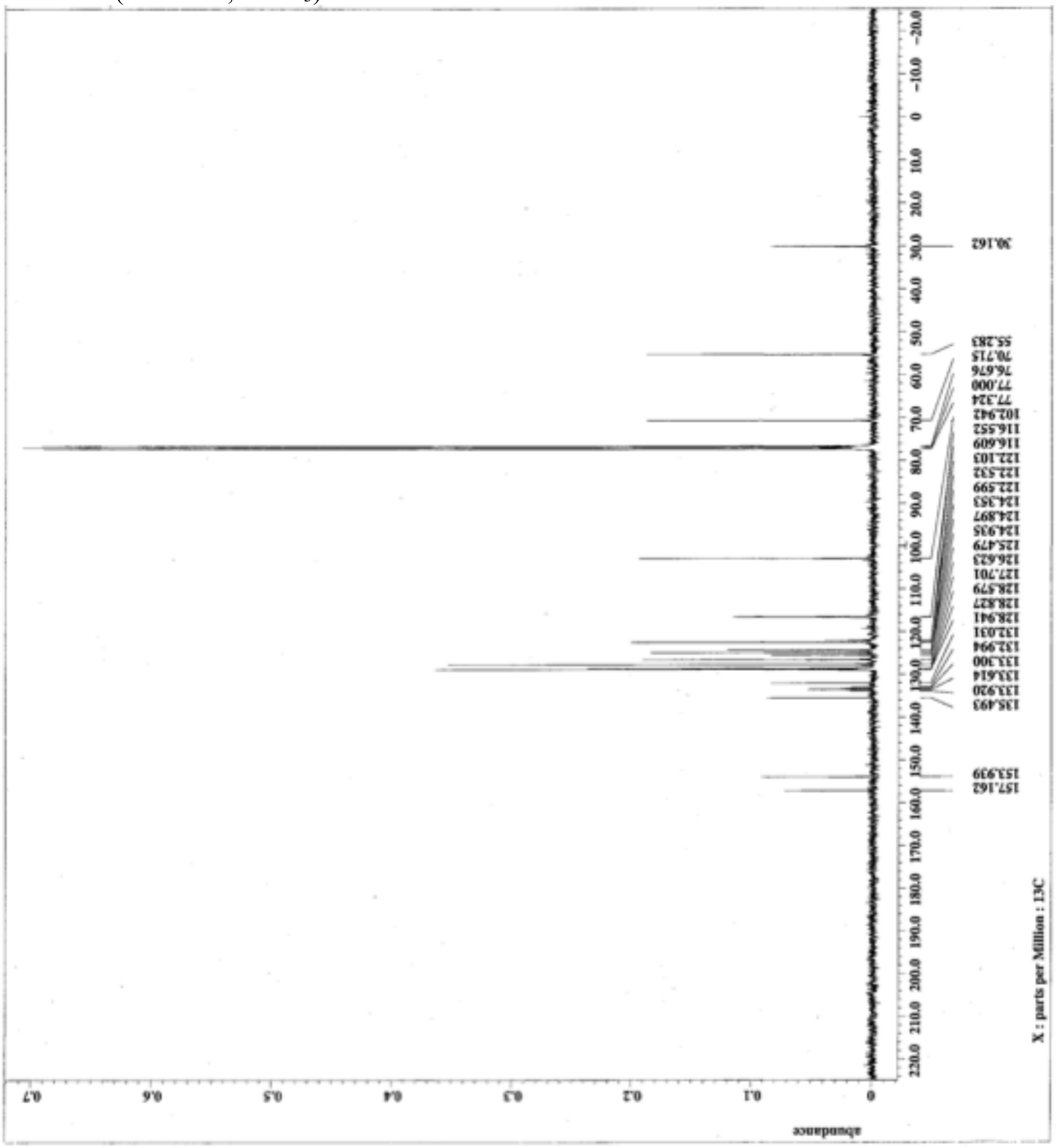

^{19}F-NMR (376 MHz, $CDCl_3$) of **7**

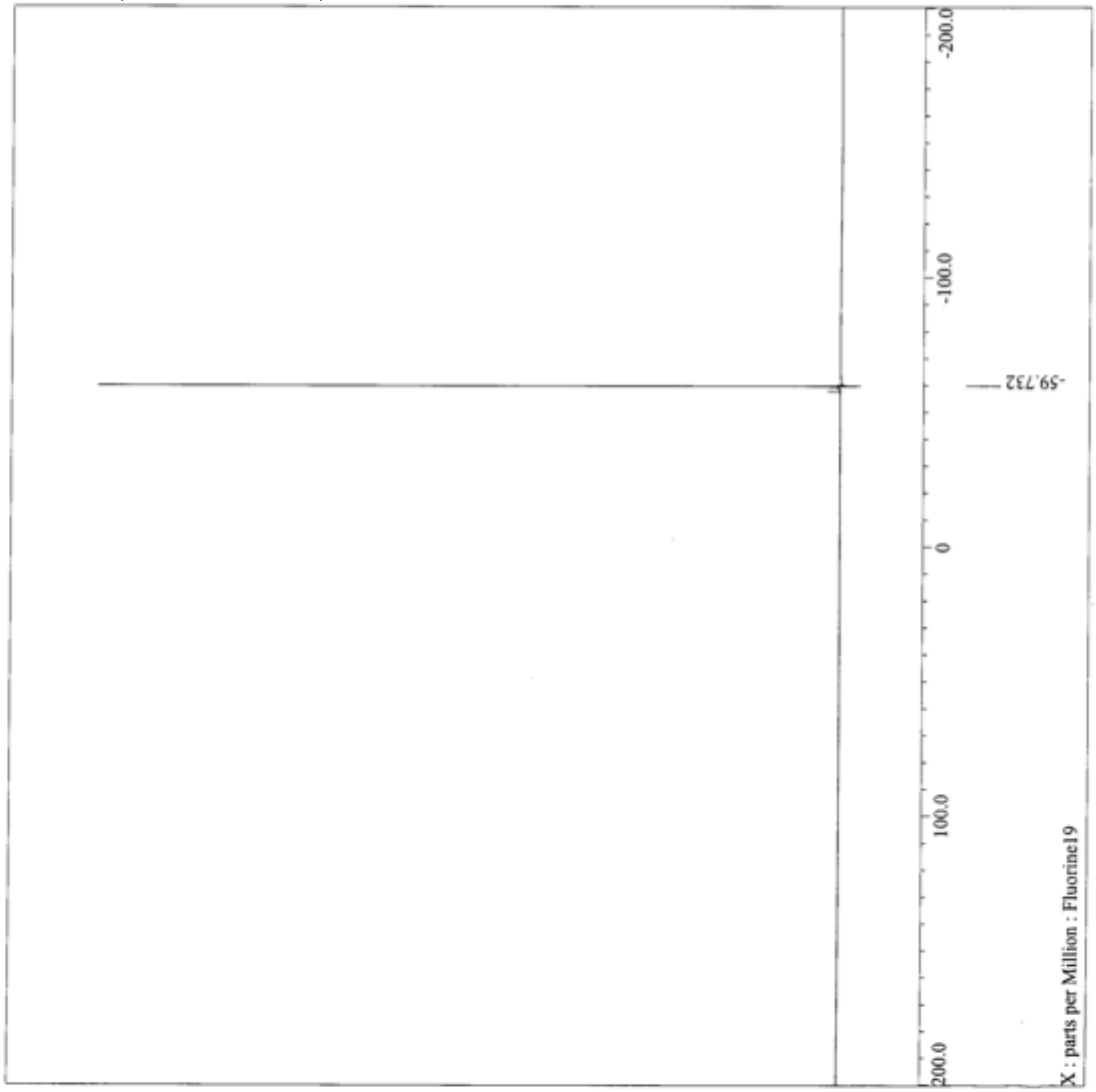

^{1}H-NMR (400 MHz, $CDCl_3$) of **8**

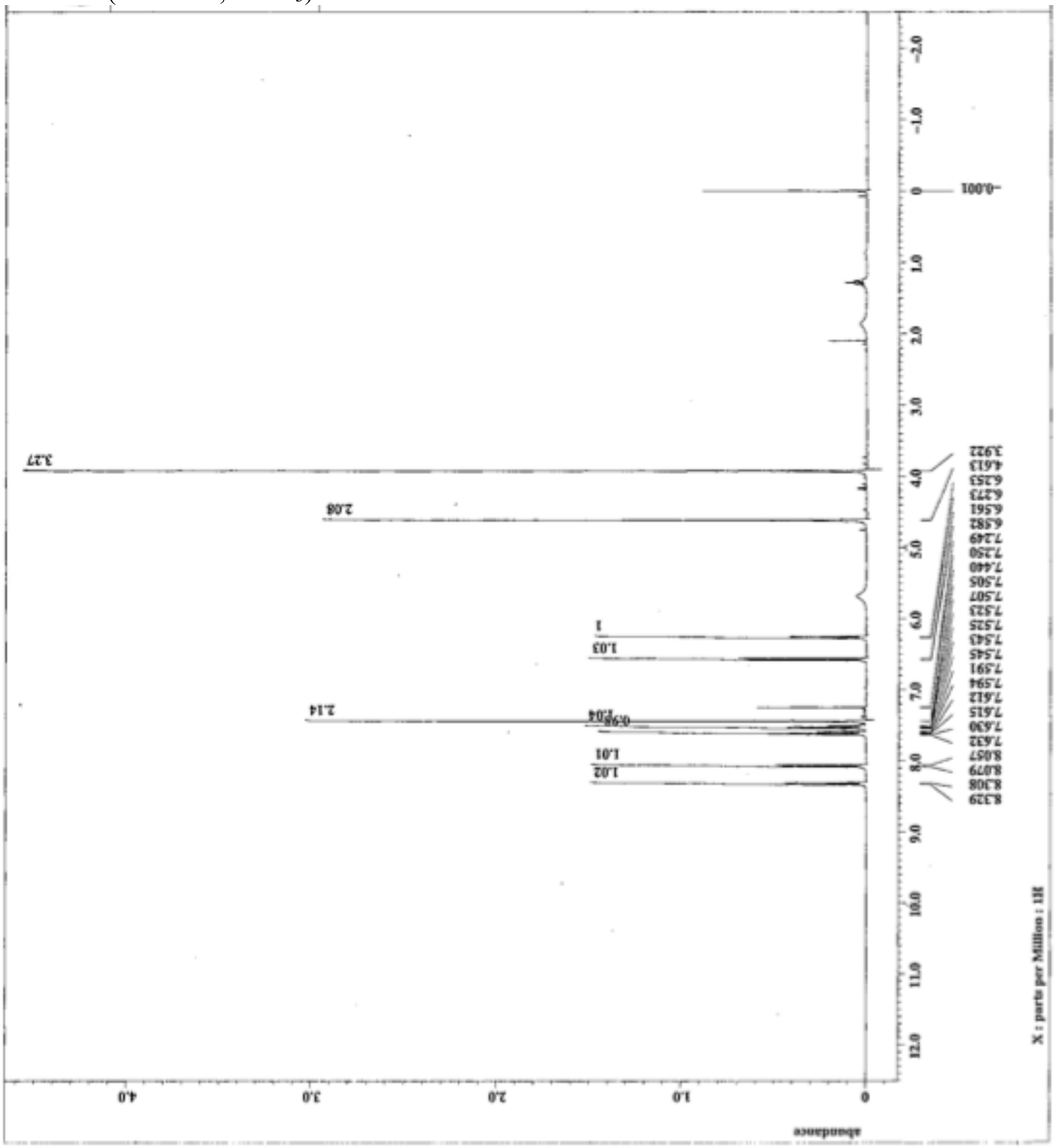

^{13}C-NMR (100 MHz, $CDCl_3$) of **8**

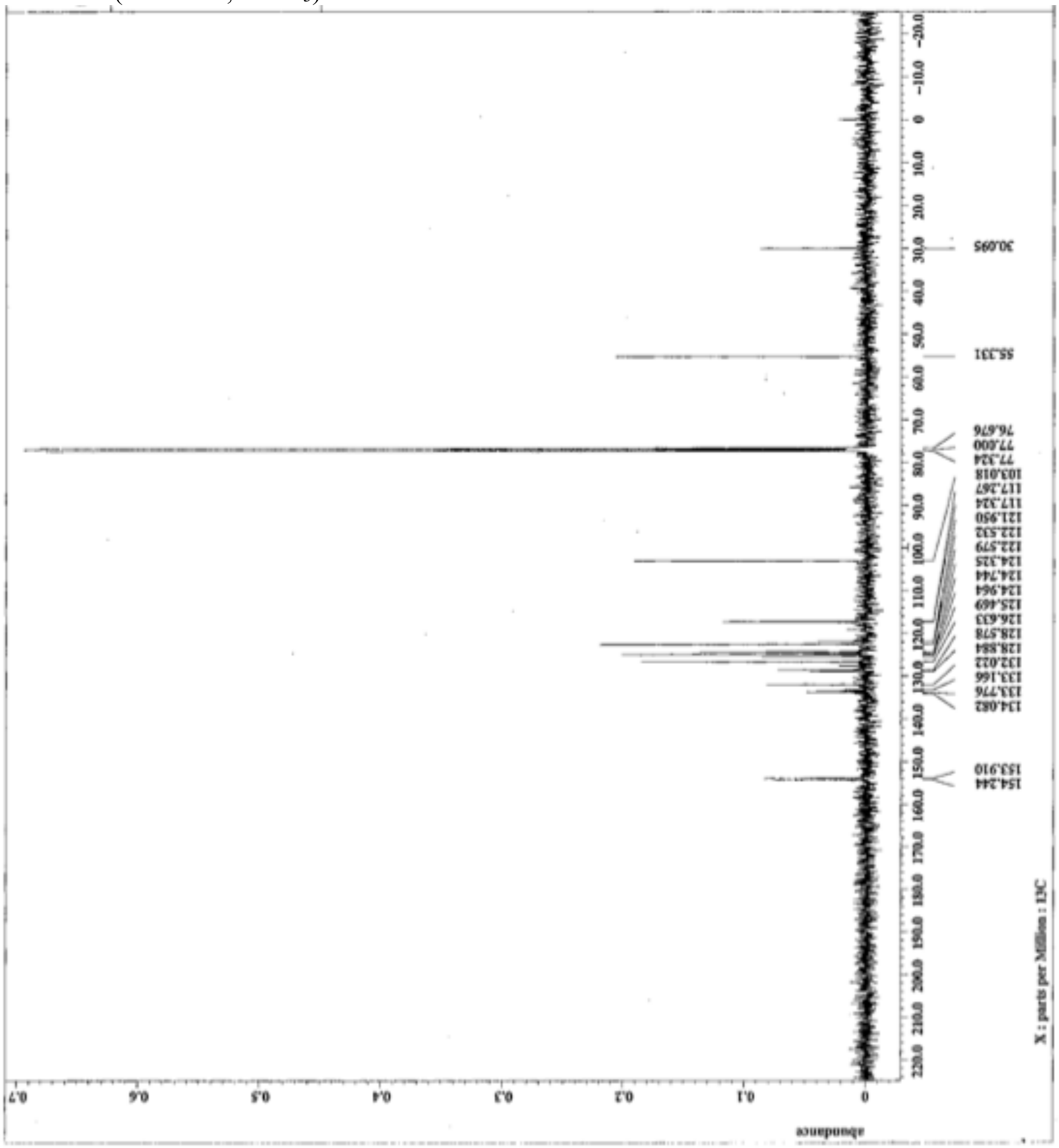

^{19}F-NMR (376 MHz, $CDCl_3$) of **8**

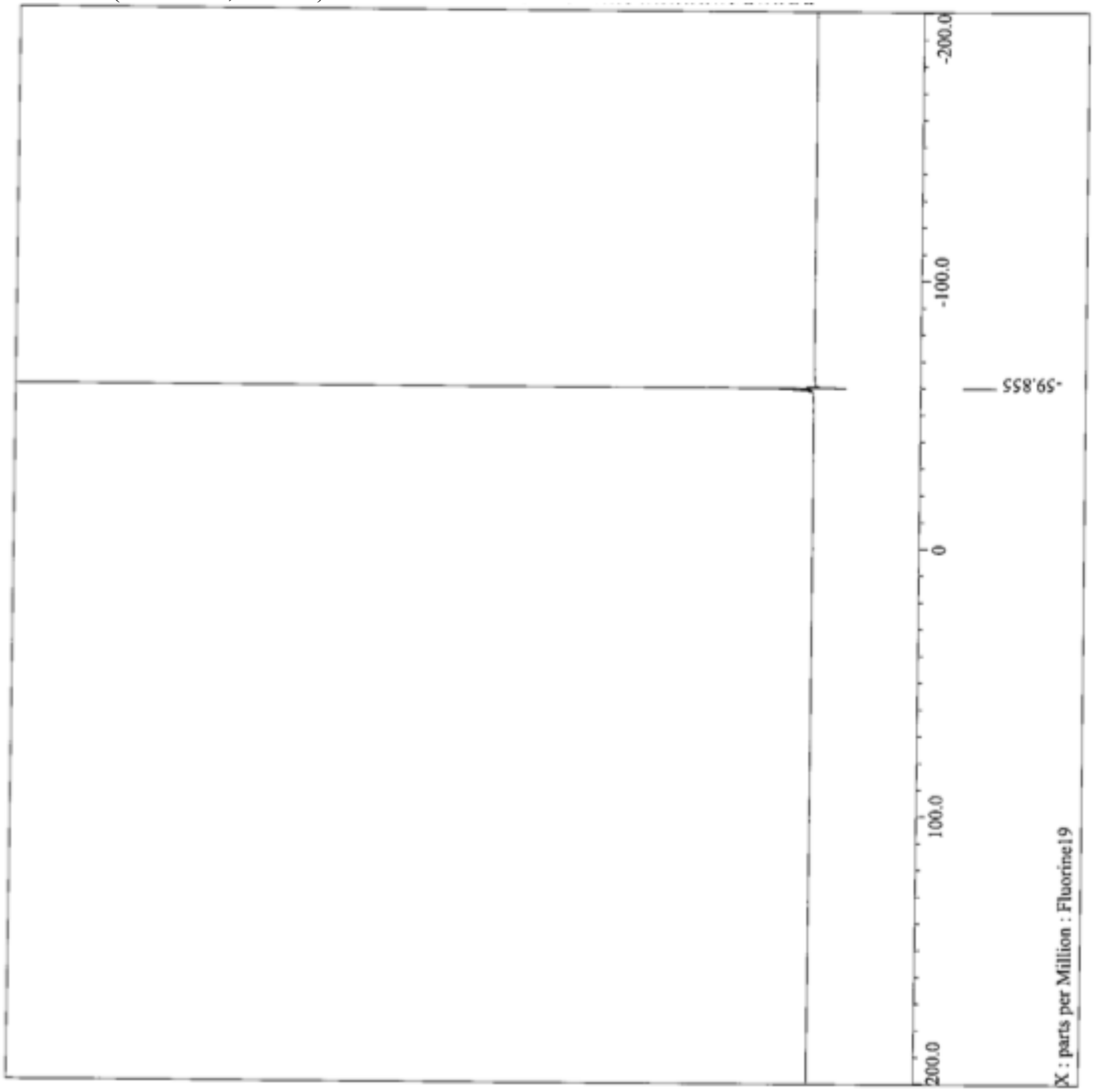

^{1}H-NMR (400 MHz, $CDCl_3$) of **9**

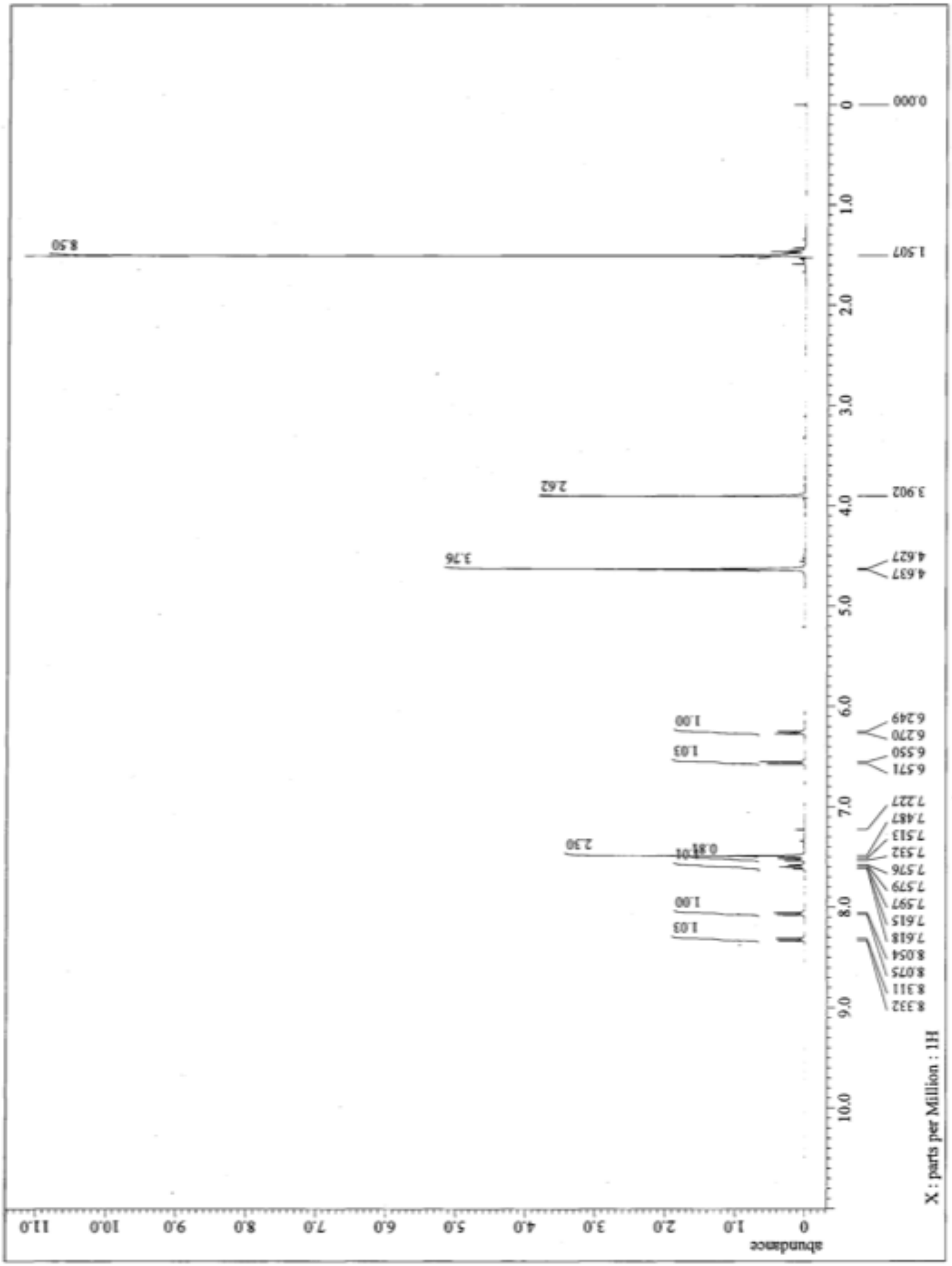

^{13}C-NMR (100 MHz, $CDCl_3$) of **9**

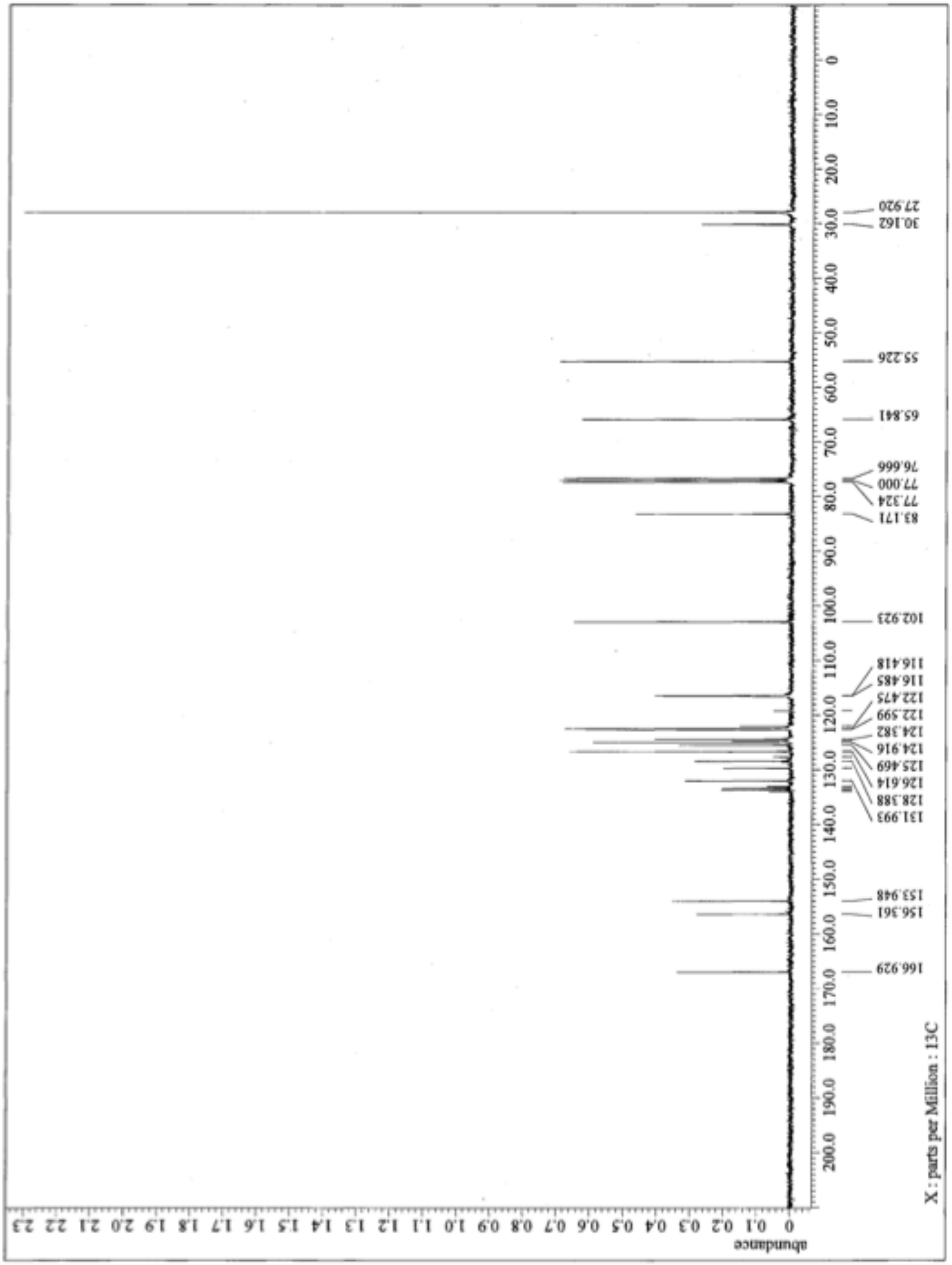

^{19}F-NMR (376 MHz, $CDCl_3$) of **9**

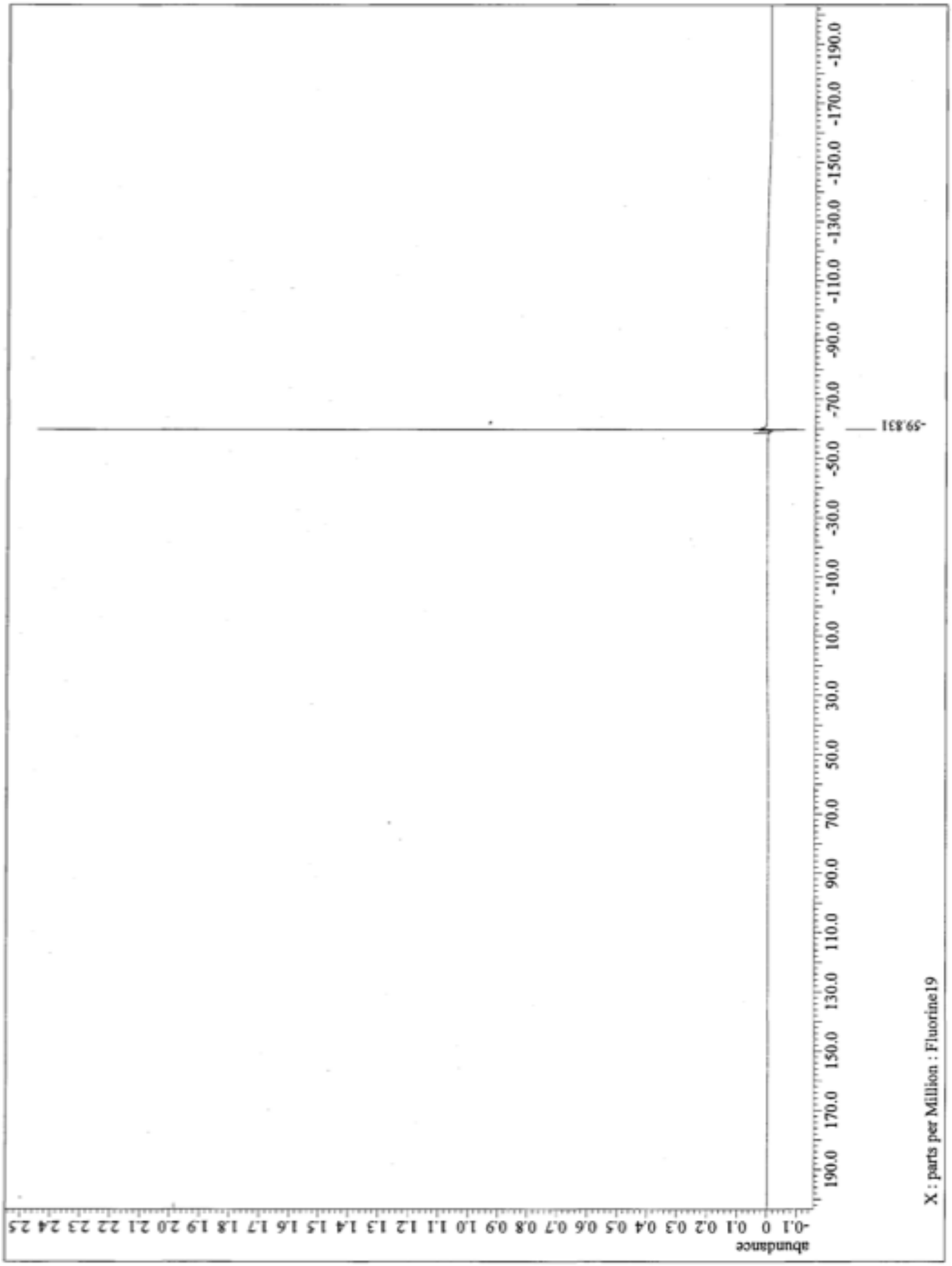

^{1}H-NMR (400 MHz, $CDCl_3$) of ZTA-245

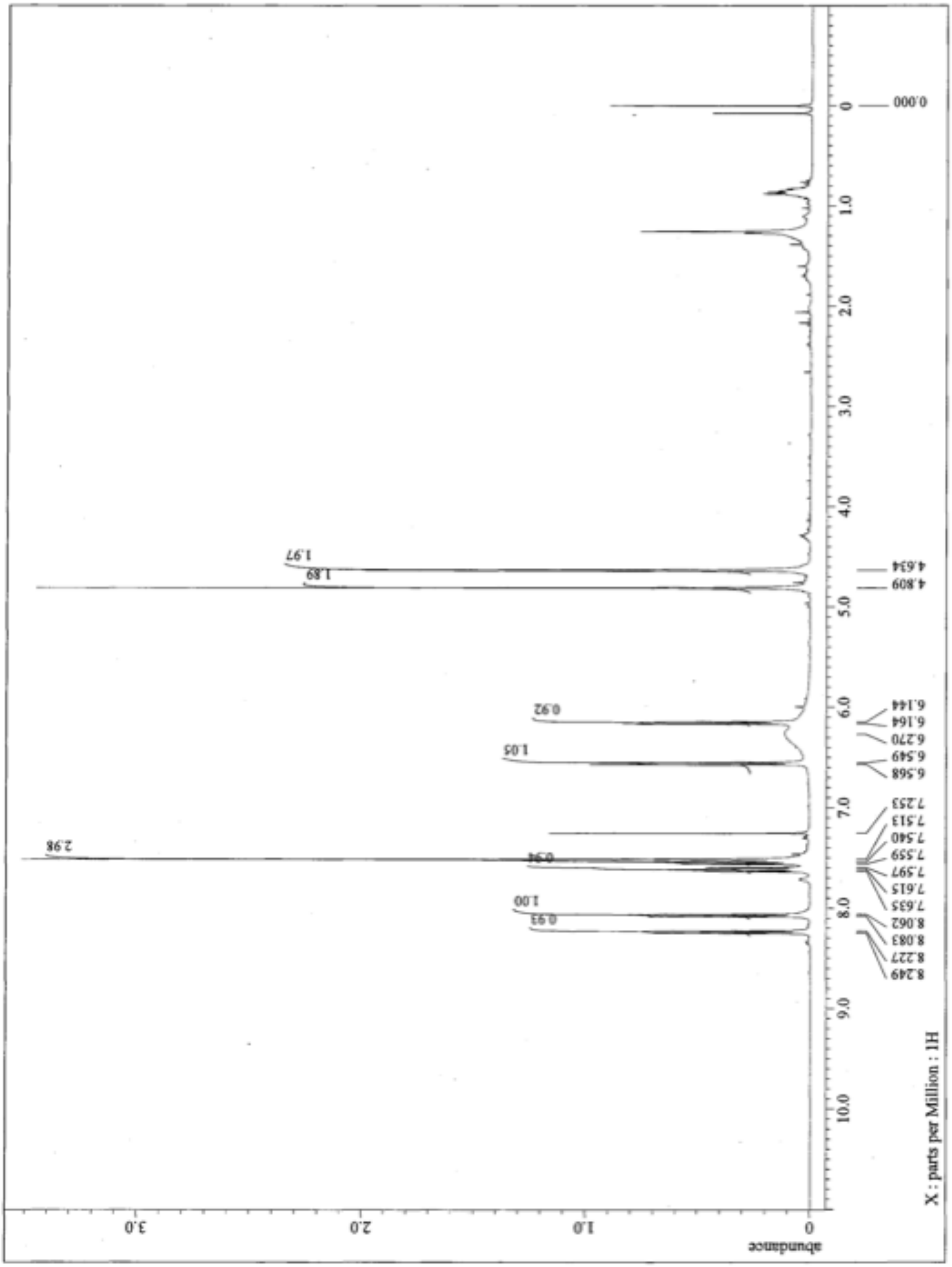

^{13}C-NMR (100 MHz, $CDCl_3$) of ZTA-245

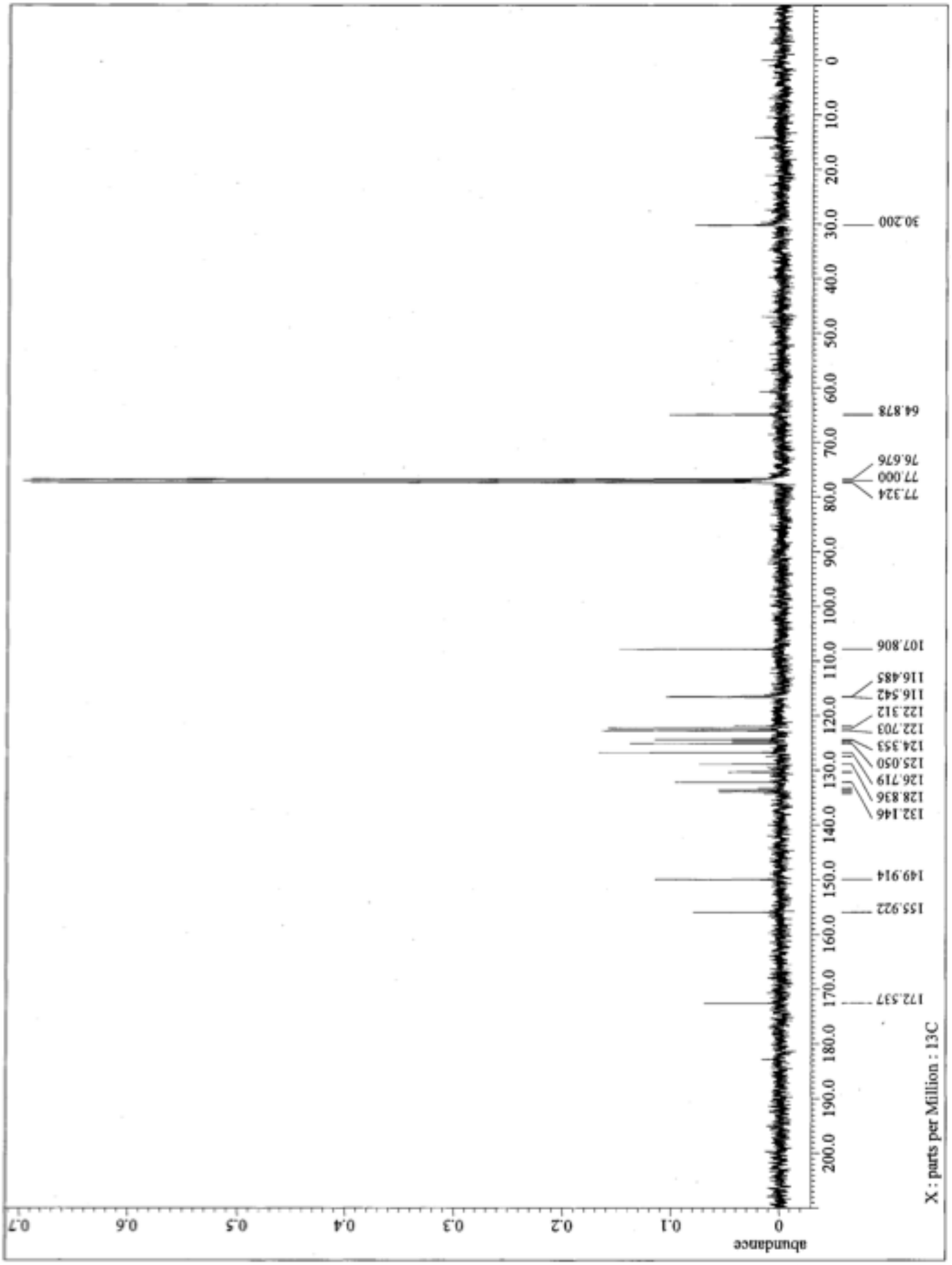

^{19}F-NMR (376 MHz, $CDCl_3$) of ZTA-245

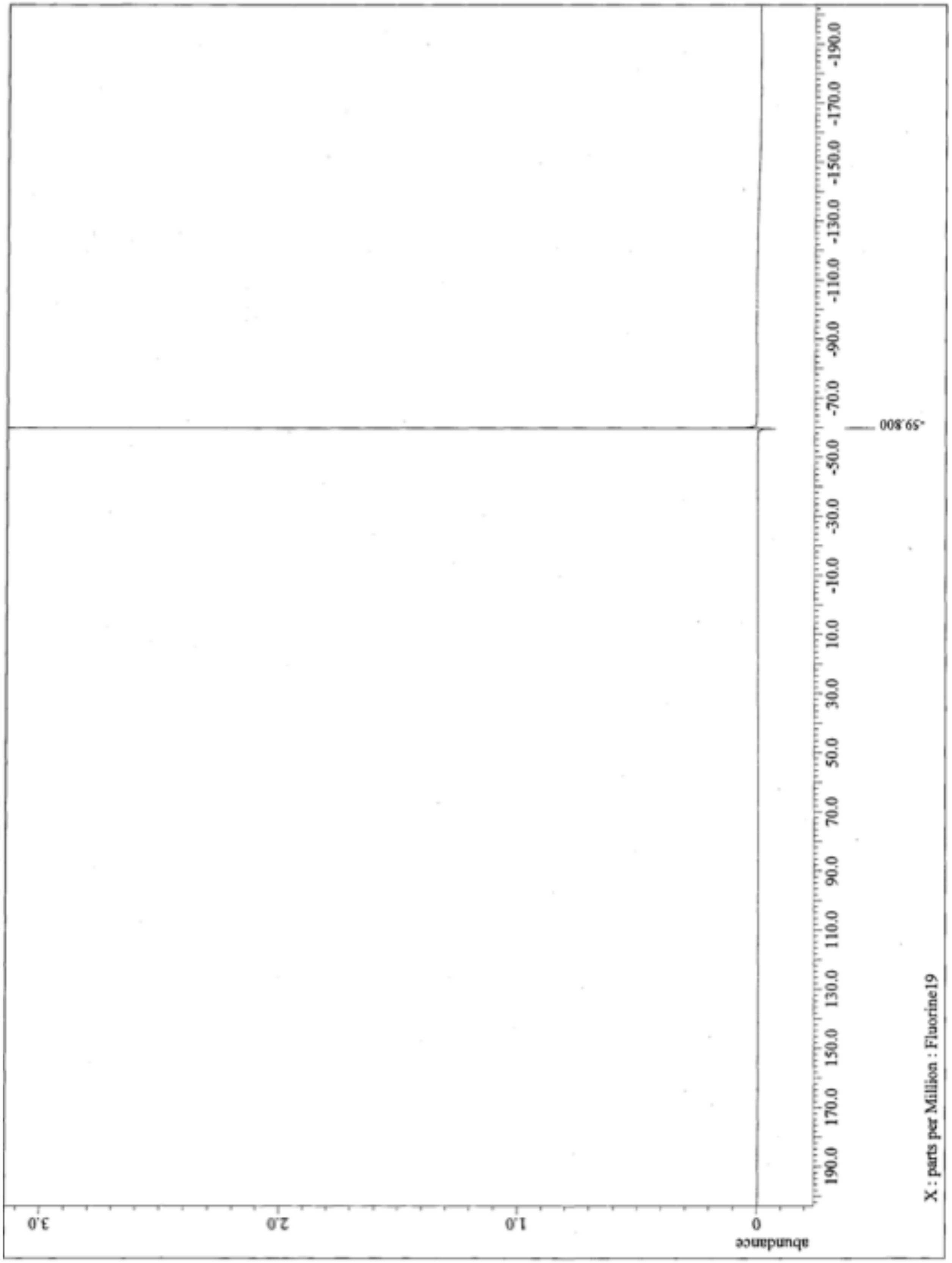

^{1}H-NMR (400 MHz, $CDCl_3$) of **10**

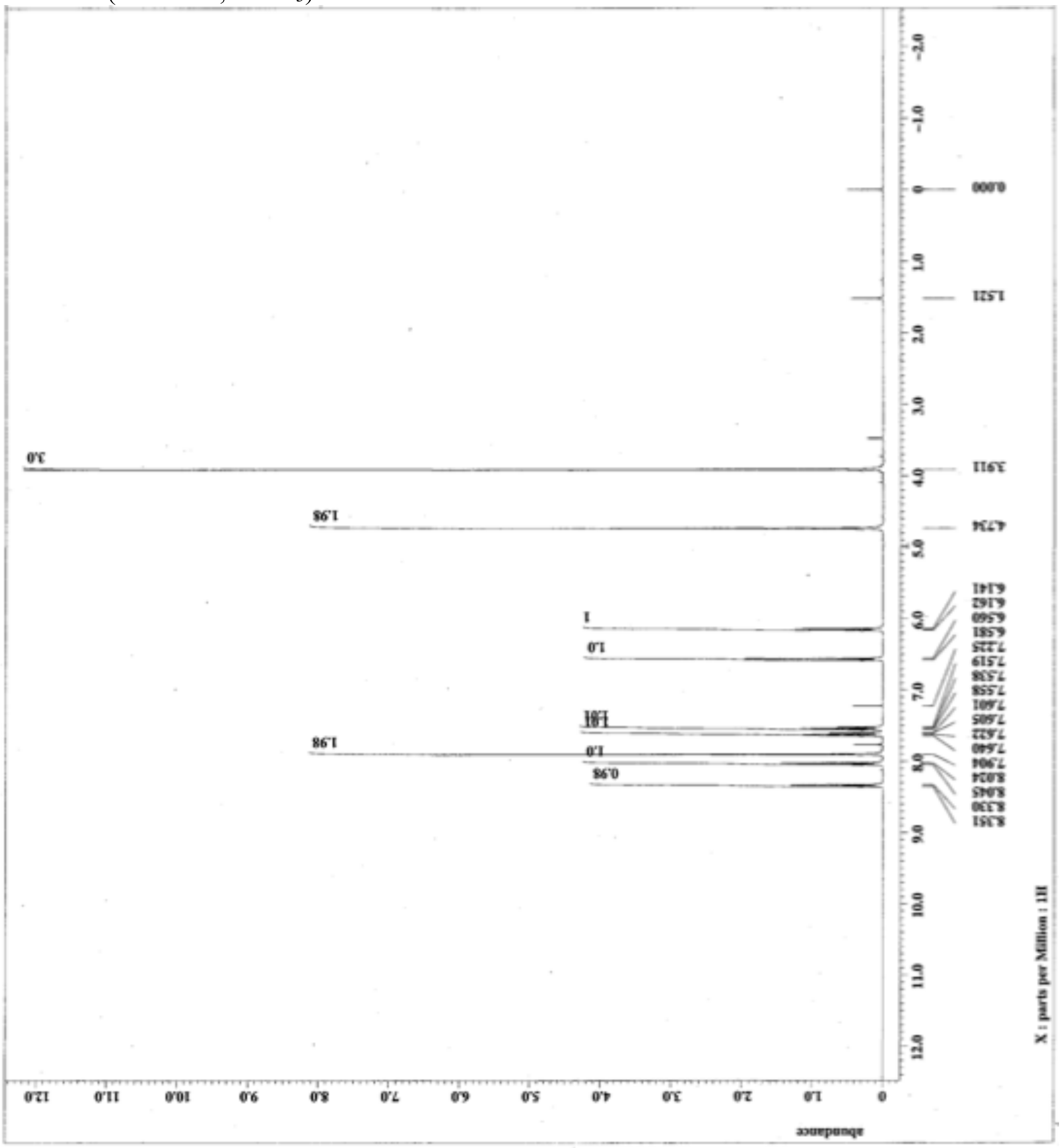

^{13}C-NMR (100 MHz, $CDCl_3$) of **10**

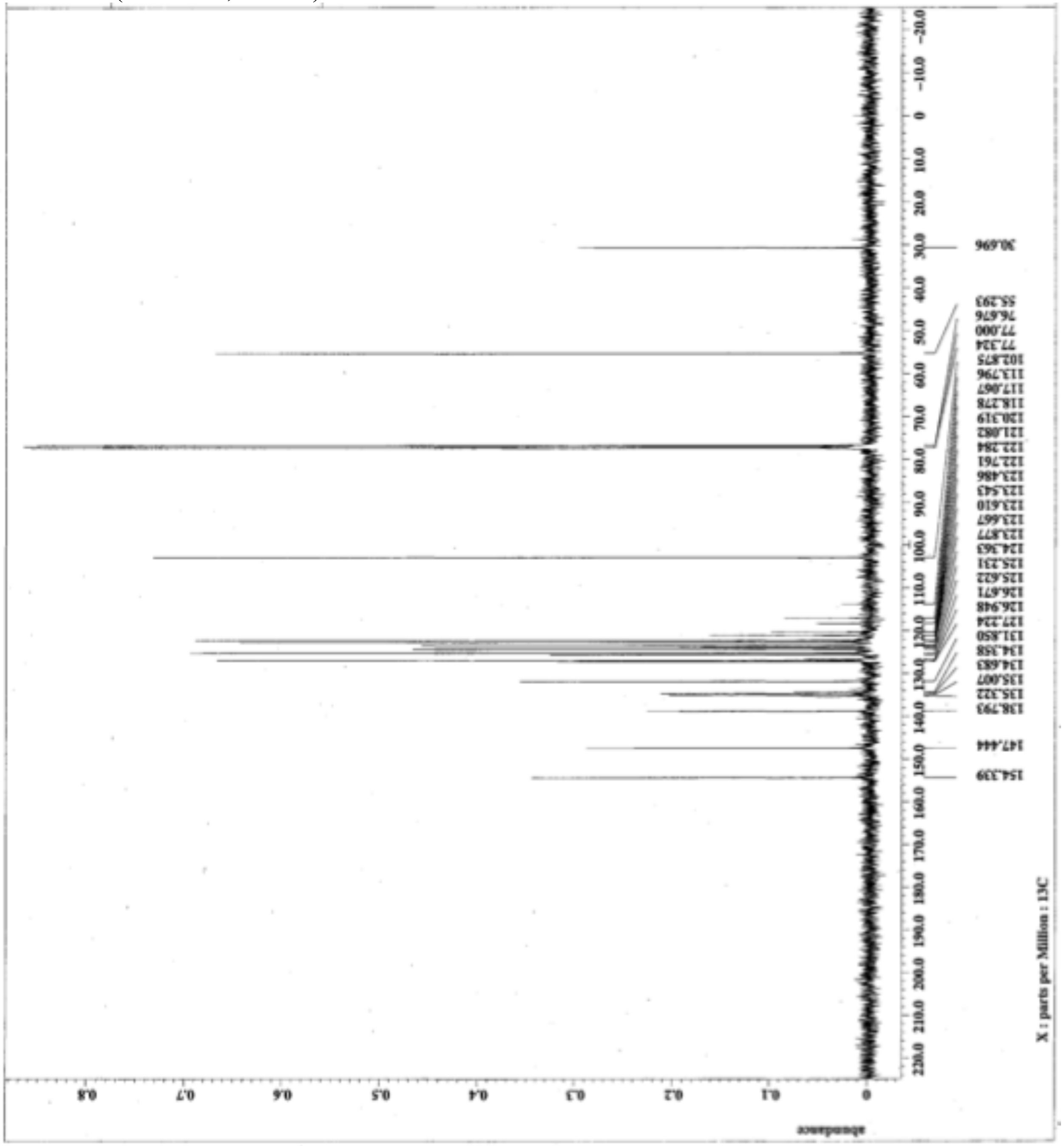

^{19}F-NMR (376 MHz, $CDCl_3$) of **10**

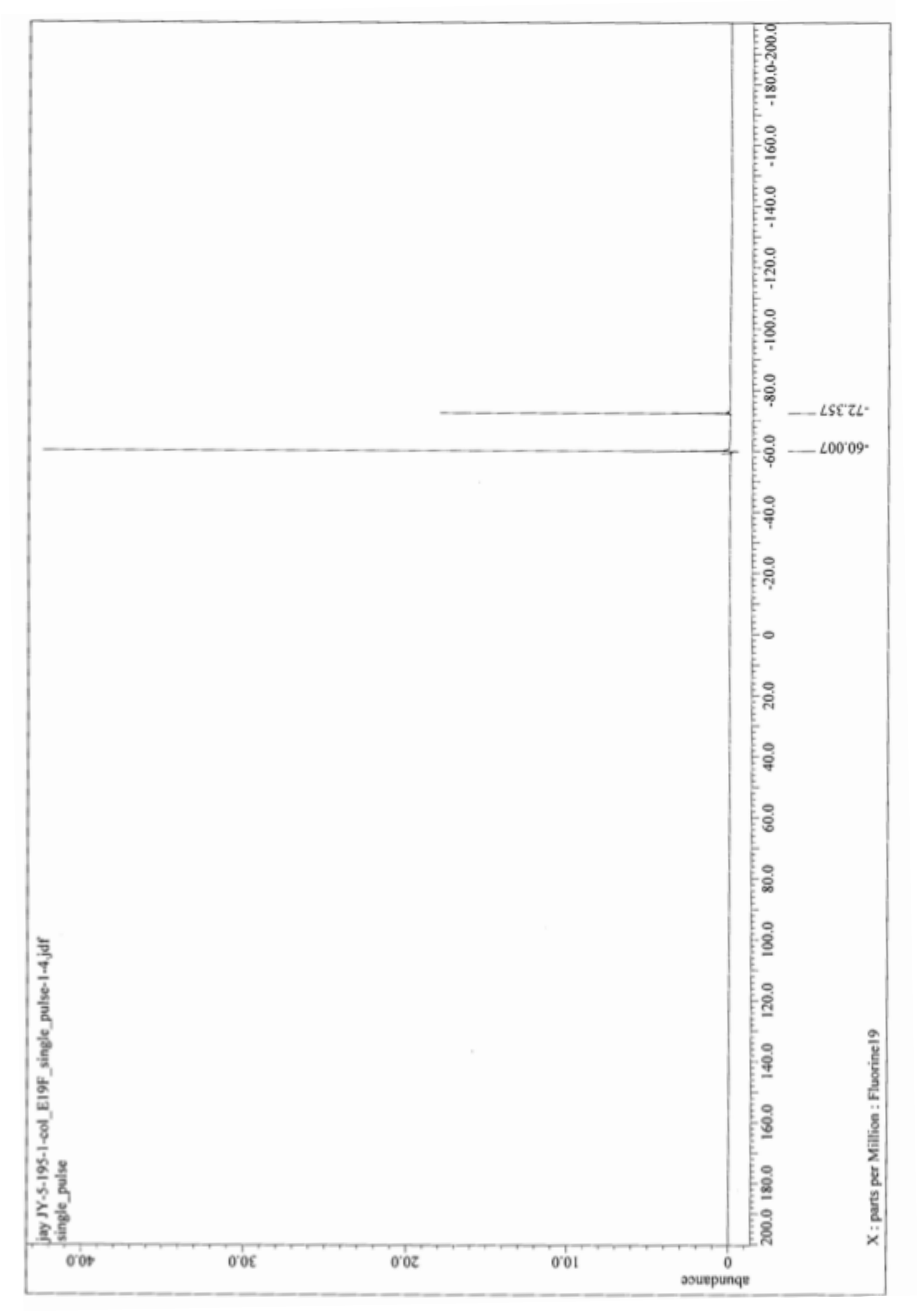

^{1}H-NMR (400 MHz, $CDCl_3$) of **11**

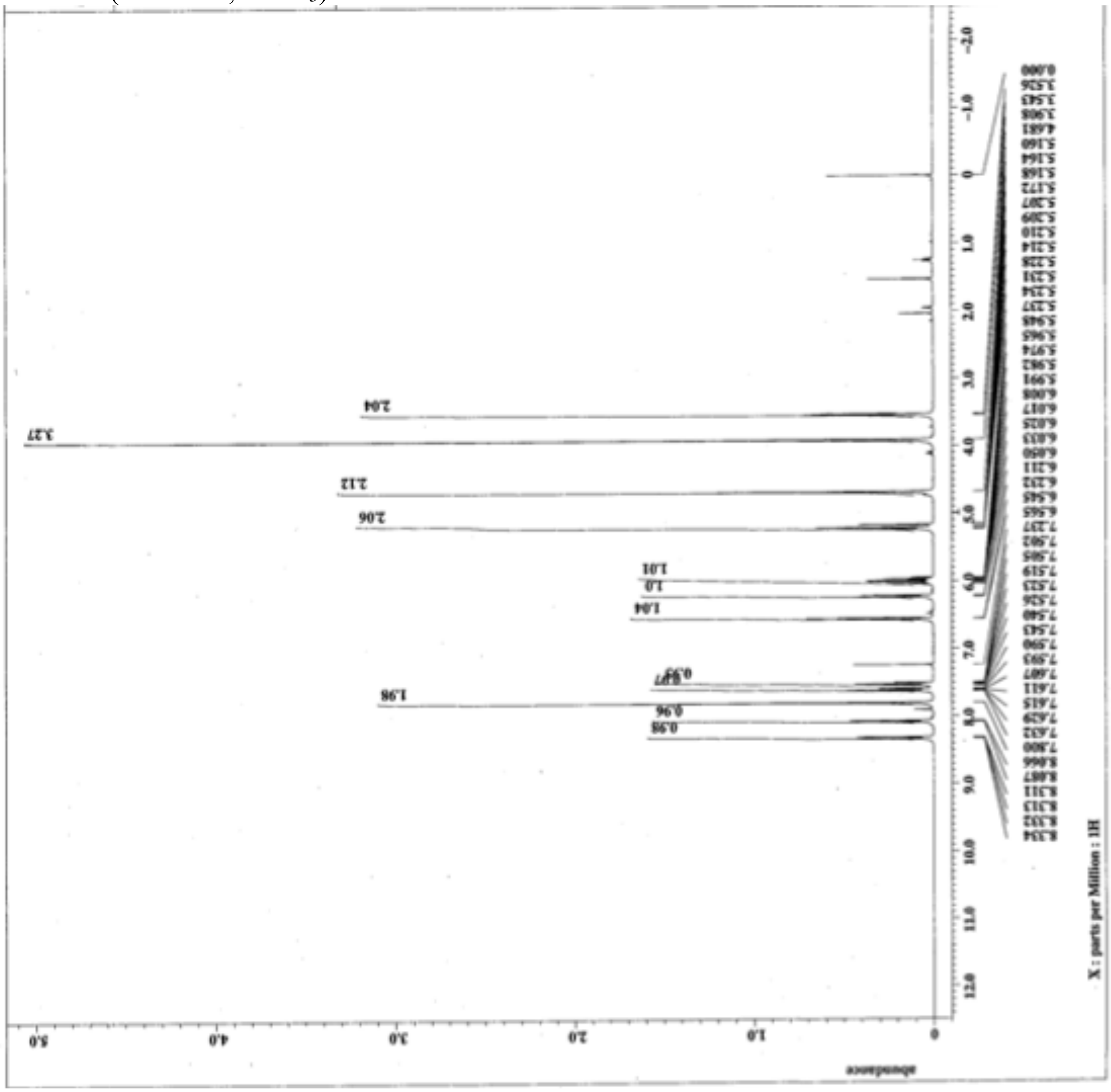

^{13}C-NMR (100 MHz, $CDCl_3$) of **11**

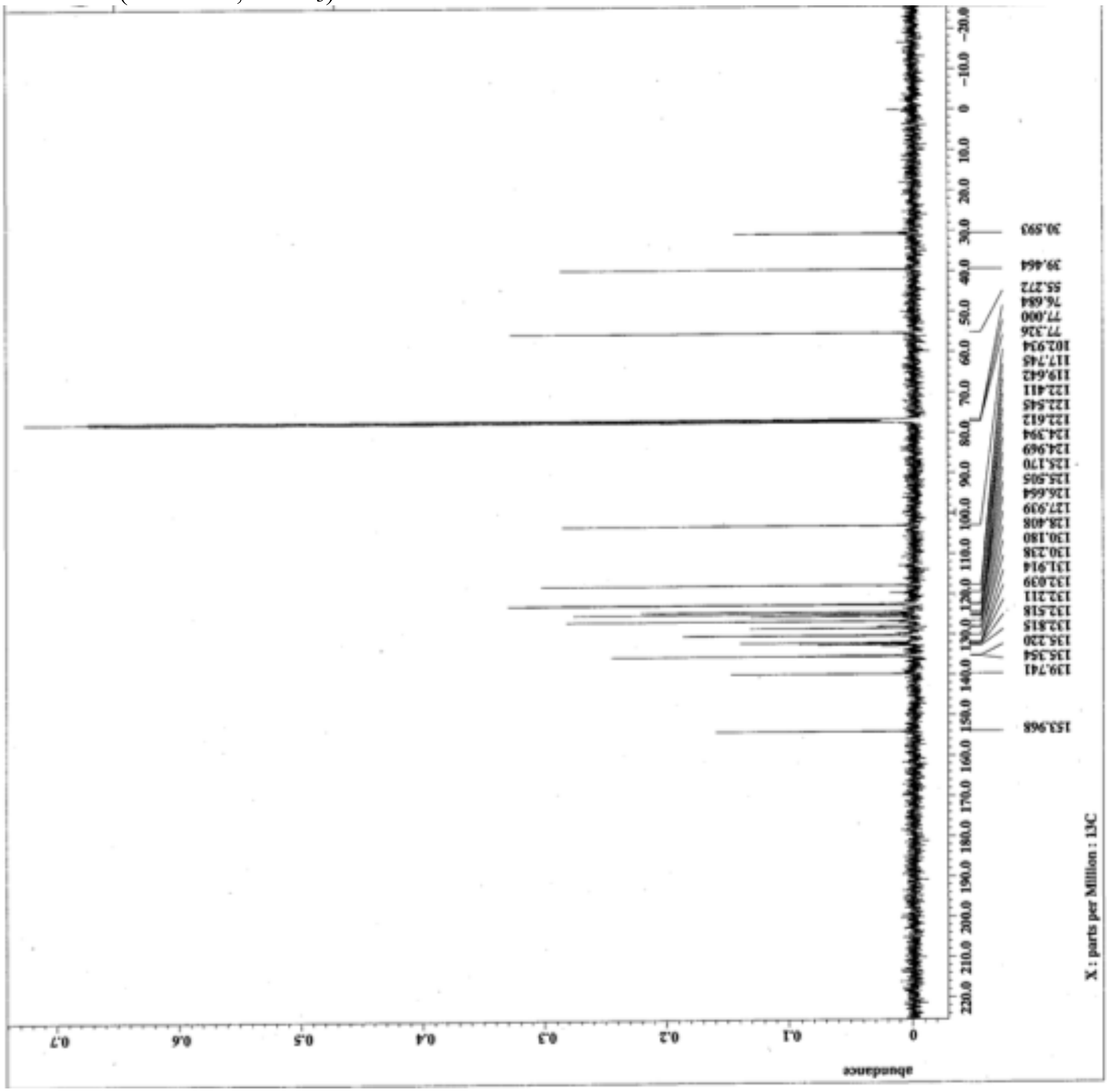

^{19}F-NMR (376 MHz, $CDCl_3$) of **11**

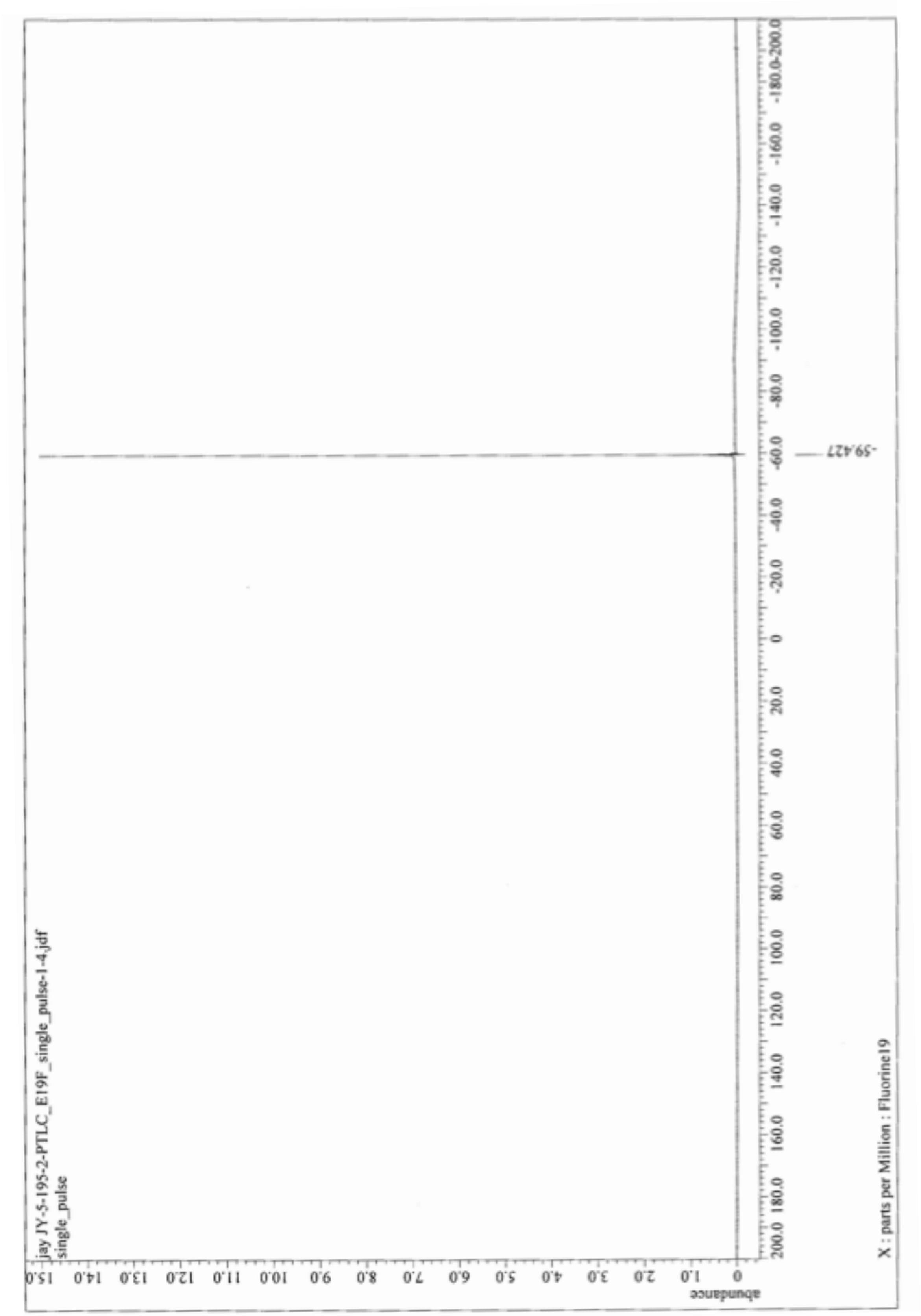

^{1}H-NMR (400 MHz, $CDCl_3$) of ZTA-261

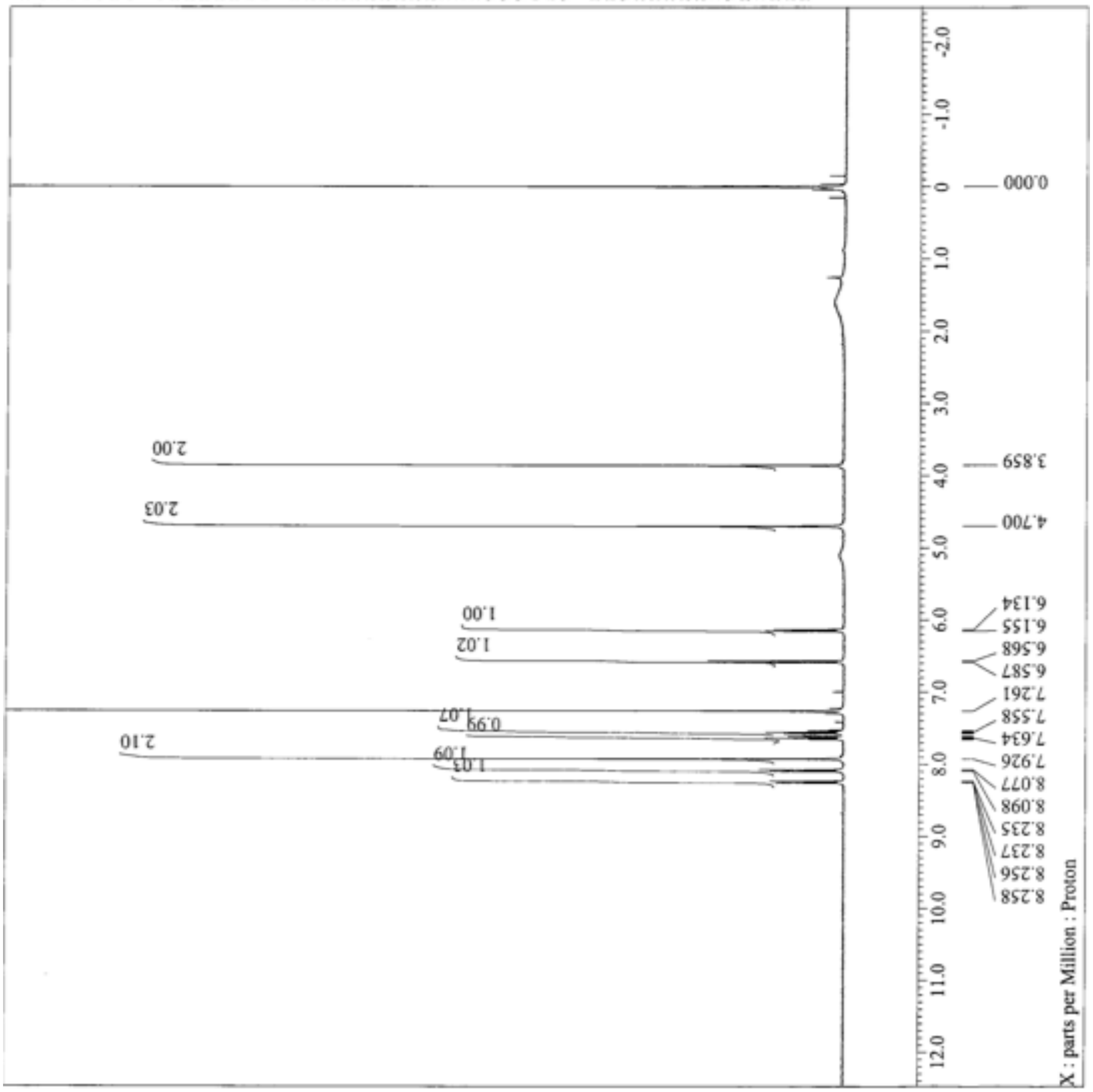

^{13}C-NMR (100 MHz, $CDCl_3$) of ZTA-261

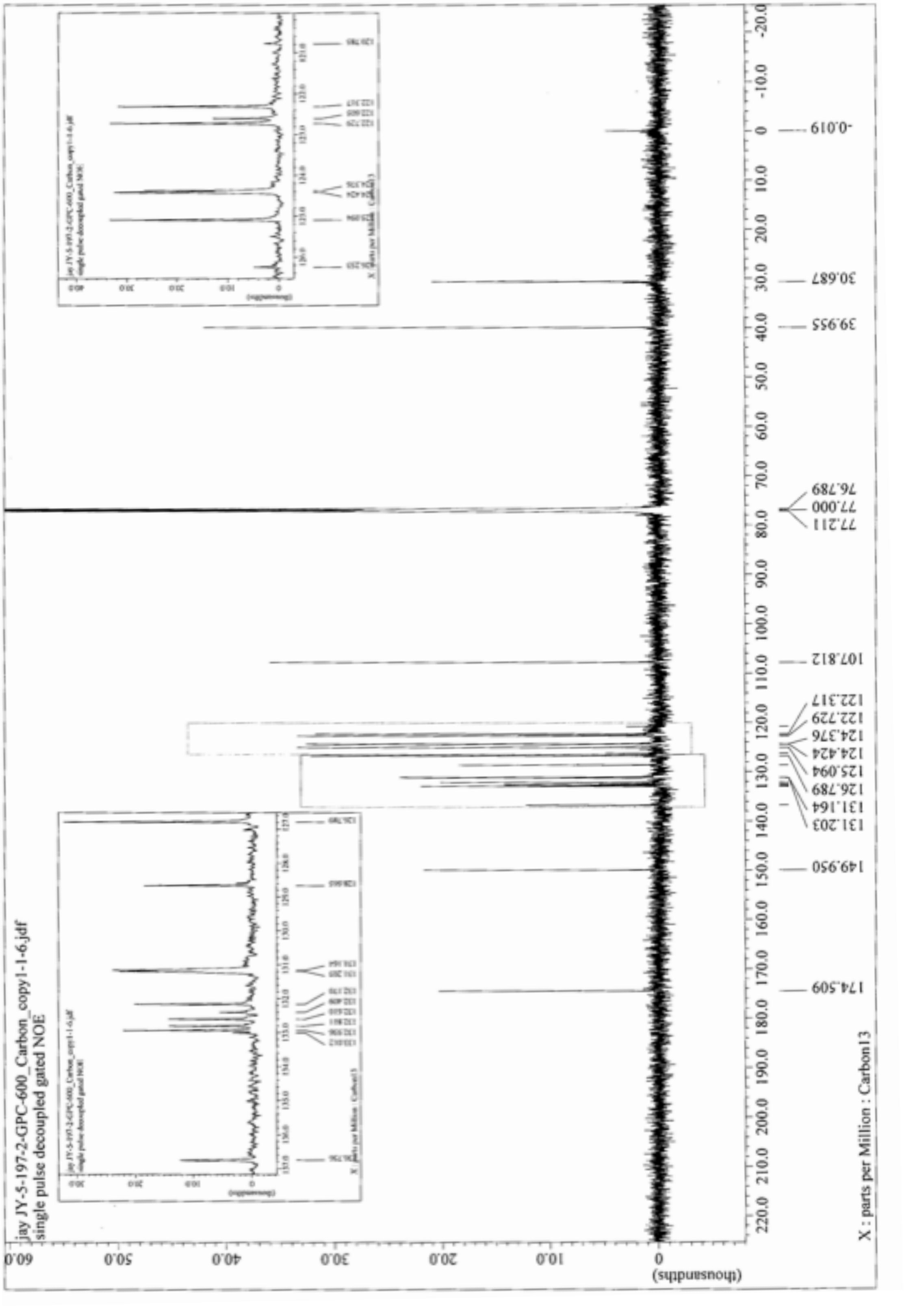

^{19}F-NMR (376 MHz, $CDCl_3$) of ZTA-261

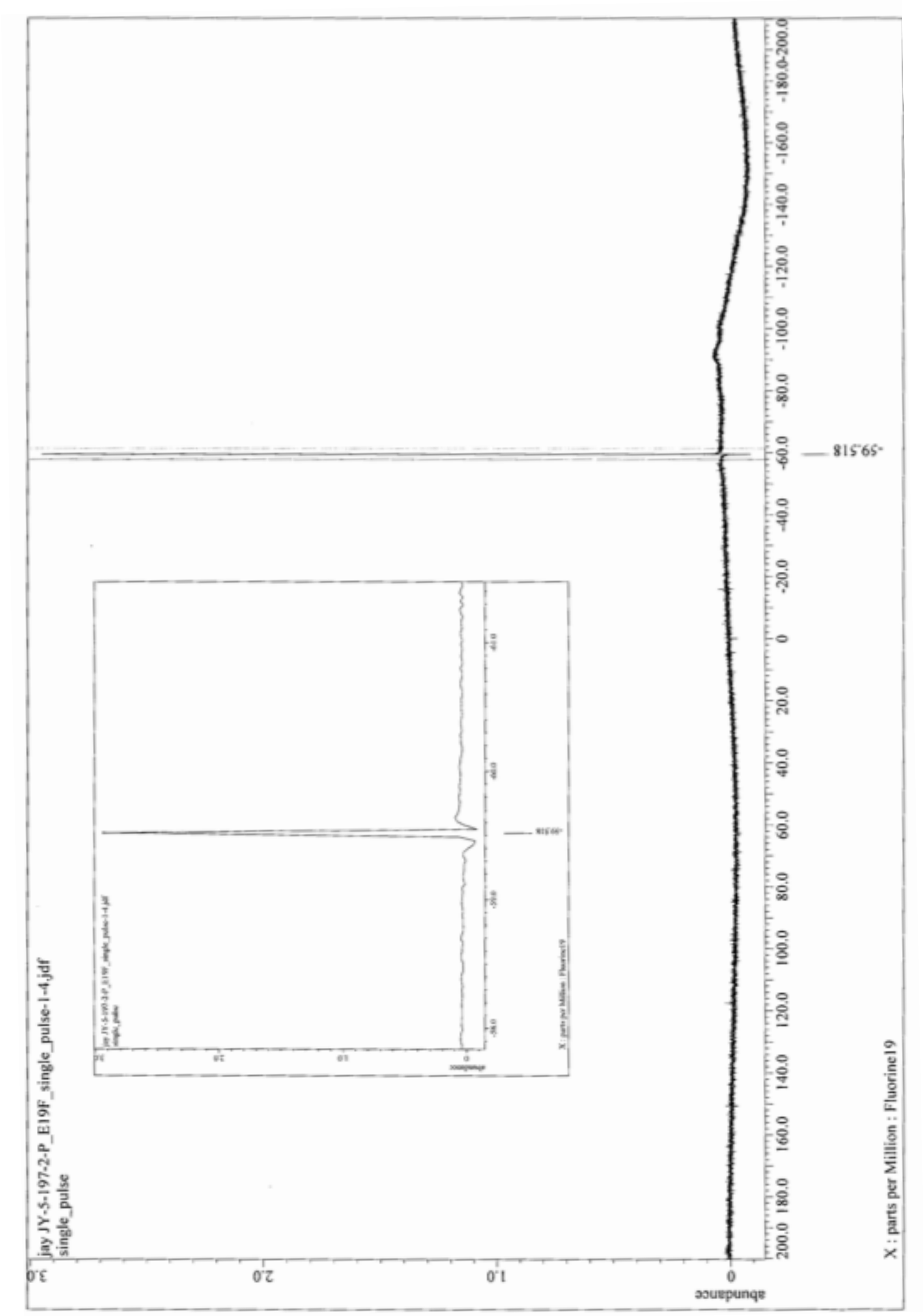

Supplementary References

[1]. Grazia Chiellini, Ngoc-Ha Nguyen, Hikari A. I. Yoshihara and Thomas S. Scanlan, *Bioorg. Med. Chem. Lett.* **10**, 2607-2611 (2000).

[2]. Nakamura, Y.; Inomata, S.; Ebine, M.; Manabe, Y.; Iwakura, I.; Ueda, M. *Org. Biomol. Chem.* **9**, 83-85 (2011).

[3]. Arif Music, Andreas N. Baumann, Philipp Spieß, Allan Plantefol, Thomas C. Jagau, Dorian Didier, *J. Am. Chem. Soc.* **142**, 4341-4348 (2020).

[4]. Xie F., Wang J. & Zhang B. RefFinder: a web-based tool for comprehensively analyzing and identifying reference genes. *Funct. Integr. Genomics* **23**, 125 (2023)

[5]. Xie F. et al. miRDeepFinder: a miRNA analysis tool for deep sequencing of plant small RNAs. *Plant Mol. Biol.* **80**, 75–84 (2012).

[6]. Nakagawa S. *et al.* A new blood-brain barrier model using primary rat brain endothelial cells, pericytes and astrocytes. *Neurochem. Int.* 54, 253-263 (2009).
