# Supplementary material for: Synthesis and preclinical testing of a selective beta-subtype agonist of thyroid hormone receptor ZTA-261
Source: Commun Med (Lond). 2024 Aug 6;4:152. doi: 10.1038/s43856-024-00574-z (PMC11303563; doi:10.1038/s43856-024-00574-z)
Supplement: Supplementary file 3 — Description of Additional Supplementary Files [file 43856_2024_574_MOESM3_ESM.pdf]

Description of Additional Supplementary Files

File name: Supplementary Data

File Description: All source data for the figures and tables presented in this article
