# Supplementary material for: Synthesis and preclinical testing of a selective beta-subtype agonist of thyroid hormone receptor ZTA-261
Source: Commun Med (Lond). 2024 Aug 6;4:152. doi: 10.1038/s43856-024-00574-z (PMC11303563; doi:10.1038/s43856-024-00574-z)
Supplement: Supplementary file 12 — Reporting Summary [file 43856_2024_574_MOESM12_ESM.pdf]

nature portfolio

Corresponding author(s): Masakazu Nambo
Taeko Nishiwaki-Ohkawa
Ayato Sato
Cathleen M. Crudden
Takashi Yoshimura

Last updated by author(s): Jun 10, 2024

Reporting Summary

Nature Portfolio wishes to improve the reproducibility of the work that we publish. This form provides structure for consistency and transparency in reporting. For further information on Nature Portfolio policies, see our Editorial Policies and the Editorial Policy Checklist.

Statistics

For all statistical analyses, confirm that the following items are present in the figure legend, table legend, main text, or Methods section.

n/a	Confirmed	
☐	☒	The exact sample size (*n*) for each experimental group/condition, given as a discrete number and unit of measurement
☐	☒	A statement on whether measurements were taken from distinct samples or whether the same sample was measured repeatedly
☐	☒	The statistical test(s) used AND whether they are one- or two-sided<br>*Only common tests should be described solely by name; describe more complex techniques in the Methods section.*
☒	☐	A description of all covariates tested
☐	☒	A description of any assumptions or corrections, such as tests of normality and adjustment for multiple comparisons
☐	☒	A full description of the statistical parameters including central tendency (e.g. means) or other basic estimates (e.g. regression coefficient) AND variation (e.g. standard deviation) or associated estimates of uncertainty (e.g. confidence intervals)
☐	☒	For null hypothesis testing, the test statistic (e.g. F, t, r) with confidence intervals, effect sizes, degrees of freedom and P value noted<br>*Give P values as exact values whenever suitable.*
☒	☐	For Bayesian analysis, information on the choice of priors and Markov chain Monte Carlo settings
☒	☐	For hierarchical and complex designs, identification of the appropriate level for tests and full reporting of outcomes
☒	☐	Estimates of effect sizes (e.g. Cohen's *d*, Pearson's *r*), indicating how they were calculated

Our web collection on statistics for biologists contains articles on many of the points above.

Software and code

Policy information about availability of computer code

Data collection	Xcalibur data system (Version 4.4, Thermo Fisher Scientific)
Data analysis	GraphPad Prism version 7.0/8.0 (GraphPad Software),

For manuscripts utilizing custom algorithms or software that are central to the research but not yet described in published literature, software must be made available to editors and reviewers. We strongly encourage code deposition in a community repository (e.g. GitHub). See the Nature Portfolio guidelines for submitting code & software for further information.

Data

Policy information about availability of data

All manuscripts must include a data availability statement. This statement should provide the following information, where applicable:

- Accession codes, unique identifiers, or web links for publicly available datasets
- A description of any restrictions on data availability
- For clinical datasets or third party data, please ensure that the statement adheres to our policy

All source data for the figures and tables presented in this article can be found in the Supplementary Data and Supplementary Information files. All other data are available from the corresponding authors upon reasonable request.

Human research participants

Policy information about studies involving human research participants and Sex and Gender in Research.

Reporting on sex and gender	N/A
Population characteristics	N/A
Recruitment	N/A
Ethics oversight	N/A

Note that full information on the approval of the study protocol must also be provided in the manuscript.

Field-specific reporting

Please select the one below that is the best fit for your research. If you are not sure, read the appropriate sections before making your selection.

☒ Life sciences ☐ Behavioural & social sciences ☐ Ecological, evolutionary & environmental sciences

For a reference copy of the document with all sections, see nature.com/documents/nr-reporting-summary-flat.pdf

Life sciences study design

All studies must disclose on these points even when the disclosure is negative.

Sample size	Mice are divided into 8 groups, normal diet (ND), High-fat diet with saline (Vehicle), High fat diet with low (0.1 umol/kg/day) and high (1 umol/kg/day) doses of either T3, GC-1, or ZTA-261 treated groups. Each group consisted of 10 animals, except for the ND (n=8) and Vehicle (n=9) groups. In the ND group, one animal died during the experimental period and one animal was excluded from the analysis after finding tumor-like lesions in the liver. In the Vehicle group, one animal was excluded from the analysis because its body weight was less two standard deviations below the mean.
Data exclusions	As described above, we excluded one mouse from ND and Vehicle group.
Replication	For in vitro compound binding assay, we performed three independent experiments. We repeated three measurements in a single experiment. For biochemical assay of the materials collected from animals, we repeated three measurements from a single sample.
Randomization	The animals were randomly divided into each experimental group.
Blinding	Blinding was not considered since this study did not involve any behavioral experiments or clinical trials.

Reporting for specific materials, systems and methods

We require information from authors about some types of materials, experimental systems and methods used in many studies. Here, indicate whether each material, system or method listed is relevant to your study. If you are not sure if a list item applies to your research, read the appropriate section before selecting a response.

Materials & experimental systems

n/a	Involved in the study	
☒	☐	Antibodies
☐	☒	Eukaryotic cell lines
☒	☐	Palaeontology and archaeology
☐	☒	Animals and other organisms
☒	☐	Clinical data
☒	☐	Dual use research of concern

Methods

n/a	Involved in the study	
☒	☐	ChIP-seq
☒	☐	Flow cytometry
☒	☐	MRI-based neuroimaging

Eukaryotic cell lines

Policy information about cell lines and Sex and Gender in Research

Cell line source(s)	GloResponse™ 9XGAL4UAS-luc2P HEK293 Cell Line (E8530, Promega)

Authentication	The instructions provided by the manufacturer did not include any details regarding the authentication process.
Mycoplasma contamination	The manufacturer's instructions did not contain any description on the Mycoplasma contamination.
Commonly misidentified lines (See ICLAC register)	N/A

Animals and other research organisms

Policy information about studies involving animals; ARRIVE guidelines recommended for reporting animal research, and Sex and Gender in Research

Laboratory animals	C57BL/6J mice
Wild animals	N/A
Reporting on sex	Male
Field-collected samples	N/A
Ethics oversight	Animal Experiment Committee, Nagoya University

Note that full information on the approval of the study protocol must also be provided in the manuscript.
